# Recent advances in oxidative radical difunctionalization of *N*-arylacrylamides enabled by carbon radical reagents

**DOI:** 10.3762/bjoc.21.98

**Published:** 2025-06-24

**Authors:** Jiangfei Chen, Yi-Lin Qu, Ming Yuan, Xiang-Mei Wu, Heng-Pei Jiang, Ying Fu, Shengrong Guo

**Affiliations:** 1 Department of Chemistry, College of Arts and Sciences, Northeast Agricultural University, 600 Changjiang Road, Harbin 150030, China; 2 Department of Chemistry, Lishui University, Lishui City 323000, Zhejiang Province, People’s Republic of China; 3 Zhejiang Lvgu Biopharmaceutical Co., Ltd., No.16 Bailian Road, Nanmingshan Street, Lishui City 323000, Zhejiang Province, China

**Keywords:** carbon radical reagents, intramolecular transformations, *N*-arylacrylamides, oxidative difunctionalization, radical reactions

## Abstract

The field of radical-mediated functionalization of *N*-arylacrylamides has experienced considerable advancements in recent years, particularly in the domain of oxidative radical difunctionalization reactions employing carbon radical reagents. This approach provides a powerful and versatile strategy for the concurrent introduction of two distinct functional groups across the double bond of *N*-arylacrylamides, facilitating the rapid construction of complex molecular architectures. This review aims to summarize the diverse strategies for inducing intramolecular transformations of *N*-arylacrylamides using various carbon radical reagents, including methods initiated by photonic, thermal, or electrochemical processes, which have been extensively investigated by researchers.

## Introduction

Alkenes, as abundant and versatile feedstocks, have been widely employed in organic synthesis, pharmaceutical development, and agrochemical production, representing one of the most significant classes of unsaturated organic compounds. Due to their broad availability and high reactivity, numerous elegant methodologies have been developed for their functionalization [1]. Among these, oxidative radical difunctionalization of *N*-arylacrylamides has emerged as a powerful strategy for the construction of diverse nitrogen-containing heterocycles, particularly oxindoles and related scaffolds. This review provides a comprehensive summary of recent advances in oxidative radical difunctionalization of *N*-arylacrylamides with a specific focus on strategies involving carbon-centered radicals. The reactions are systematically categorized according to their initiation modes and radical sources, including (1) transition-metal-catalyzed radical reactions, (2) peroxide-mediated thermal processes, (3) photoredox-catalyzed transformations, (4) electrochemical approaches, and (5) metal-free or electron donor–acceptor (EDA)-driven systems. The substrate scope, limitations, and mechanistic aspects of these radical cascade cyclization strategies are critically examined.

## Review

### *N*-Arylalkenes: alkyl C(sp^3^)–H radicals

Early investigations primarily focused on substrates containing activated alkenes tethered within the molecular framework. *N*-Arylacrylamides were employed as model substrates, and a diverse range of functionalization reagents, including those with benzylic C–H bonds, C(sp^3^)–H bonds adjacent to heteroatoms, di-/trifluoroalkylation reagents, α-carbonyl alkyl bromides/alcohols, alkyl halides, and alkyl carboxylic acids, have been successfully applied to this transformation to afford 3-substituted indolin-2-ones.

**Scheme 1:**
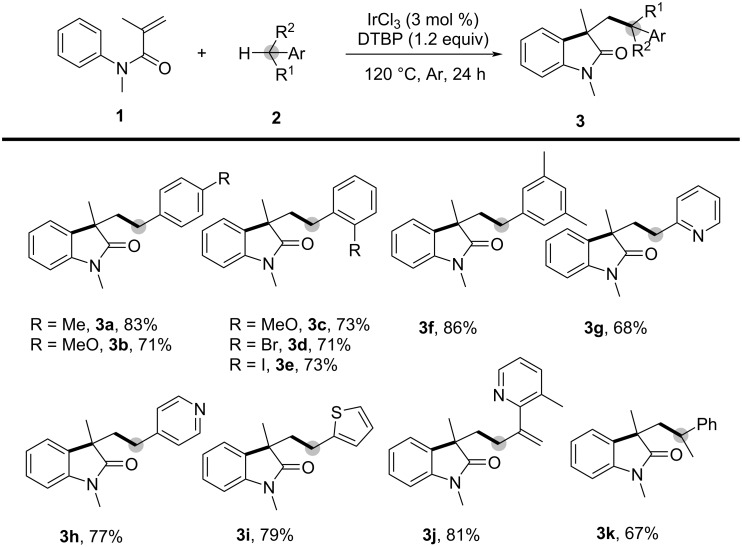
DTBP-mediated oxidative alkylarylation of activated alkenes.

In 2013, Li’s group reported a novel DTBP(di-*tert*-butyl peroxide)-mediated oxidative 1,2-alkylarylation of activated alkenes involving benzylic C(sp^3^)–H bonds through a cascade cyclization process (Scheme 1) [2]. This organomediated approach can be facilitated by a catalytic amount of Lewis acid. Using DTBP as the oxidant and IrCl_3_ as the promoter, a range of benzylic C–H bonds in arylmethanes, heteroarylmethanes, phenylethane, and cumene proved compatible with the reaction conditions, providing oxindoles **3a**–**k** with yields ranging from 67% to 86%.

**Scheme 2:**
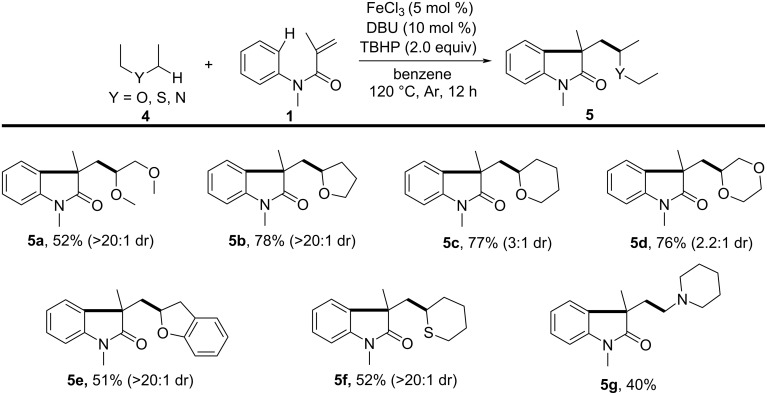
Iron-catalyzed oxidative 1,2-alkylarylation.

As illustrated in Scheme 2, an iron-catalyzed difunctionalization of alkenes involved in intramolecular transformations involving a C(sp^3^)–H bond adjacent to a heteroatom for the synthesis of functionalized oxindoles was also reported in 2013 [3]. In this study, DBU was employed as a ligand and TBHP as an oxidant. A series of ethers, including 1,2-dimethoxyethane, THF, 1,4-dioxane, tetrahydro-2*H*-pyran, 2,3-dihydrobenzofuran, tetrahydro-2*H*-thiopyran, and *N*-methylpiperidine, proved compatible with the reaction conditions, yielding the corresponding 3-(2-oxoethyl)indolin-2-ones **5a**–**g** in 40–78% yields.

**Scheme 3:**
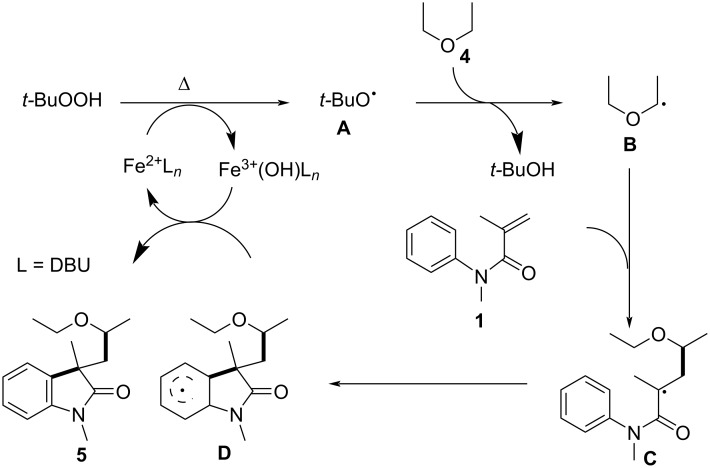
Possible mechanism for the iron-catalyzed oxidative 1,2-alkylation of activated alkenes.

Following a detailed investigation, a plausible mechanism was proposed, as illustrated in Scheme 3. Initially, TBHP undergoes cleavage by Fe^2+^ to generate a *tert*-butoxy radical **A**. This is followed by the conversion of a substrate containing C(sp^3^)–H bonds adjacent to an oxygen atom into an alkyl radical intermediate **B**. The alkyl radical intermediate then adds to the C=C bond of *N*-arylacrylamide, generating a second alkyl radical intermediate **C**, which undergoes intramolecular cyclization to form a benzene ring radical intermediate **D**. Finally, hydrogen abstraction from the radical intermediate by Fe^3+^(OH) leads to the formation of oxindole compounds **5**.

**Scheme 4:**
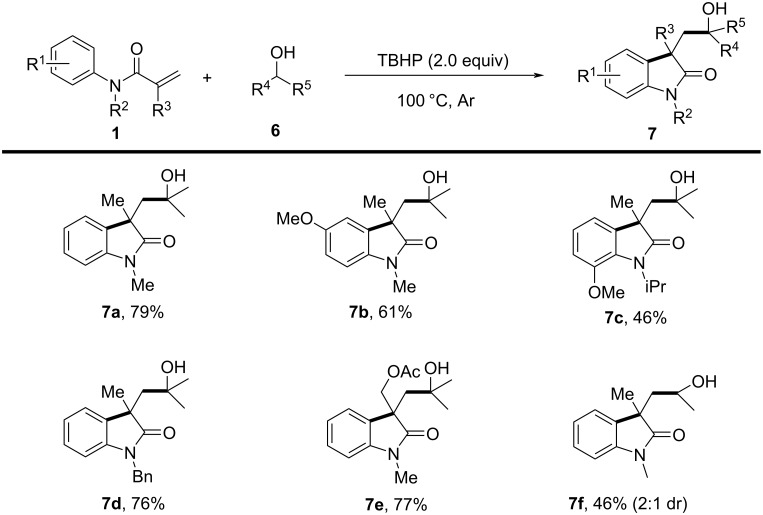
A metal-free strategy for synthesizing 3,3-disubstituted oxindoles.

A similar reaction was reported by Liang’s group in the same year, as shown in Scheme 4. The study introduced a metal-free synthetic method for 3,3-disubstituted oxindoles via 1,2-alkylarylation of activated alkenes with alcohols [4]. *N*-Arylacrylamides and simple alcohols were employed as substrates, proceeding through an oxidative radical cyclization mechanism. The standard reaction conditions involved the use of an equimolar amount of *tert*-butyl hydroperoxide (TBHP) as the oxidant, with the reaction conducted at 100 °C under an argon atmosphere, resulting in a 79% yield of the desired product **7a** without the requirement for any metal catalyst. In terms of substrate scope, the study explored various *N*-arylacrylamides and alcohols. Substrates with different substituents, including both electron-donating and electron-withdrawing groups, provided satisfactory yields (**7a**–**f**). However, steric hindrance significantly influenced the outcome, as *ortho*-substituted substrates yielded lower amounts (**7c**). The study also demonstrated that both primary and secondary alcohols were compatible substrates (**7a**, **7f**).

The proposed reaction mechanism involves oxidative radical cyclization. Initially, TBHP undergoes homolytic cleavage to generate a *tert*-butoxy radical, which then forms an α-hydroxy carbon radical. This radical subsequently adds to the activated alkene of *N*-arylacrylamide, followed by intramolecular cyclization, ultimately leading to the formation of the hydroxy-containing oxindole via the loss of a hydrogen radical.

**Scheme 5:**
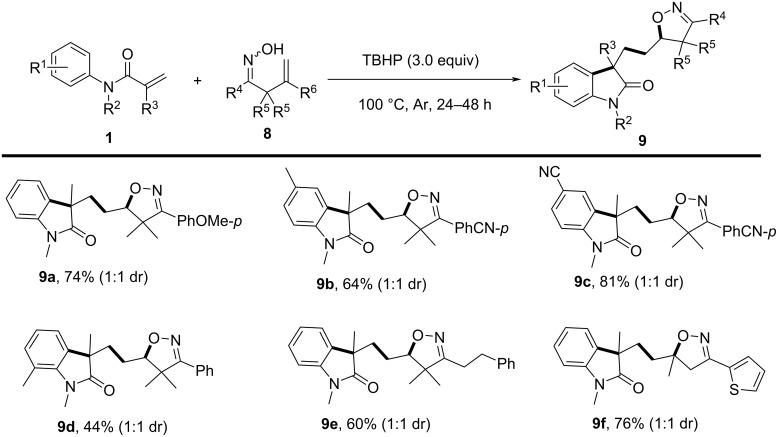
Iminoxyl radical-promoted cascade oxyalkylation/alkylarylation of alkenes.

In 2016, Han and colleagues developed a novel methodology in which TBHP served dual roles as both oxidant and initiator, enabling an efficient intermolecular cascade cyclization process (Scheme 5) [5]. In this strategy, a novel, selective, metal-free synthetic method was introduced for the synthesis of isoxazoline-featured oxindoles through iminoxyl radical-promoted cascade oxyalkylation/alkylarylation of both unactivated and activated alkenes. The reaction employed β,γ-unsaturated ketoximes and *N*-arylacrylamides as substrates, with *tert*-butyl hydroperoxide (TBHP) acting as the oxidant, conducted at 100 °C in a sealed tube under an argon atmosphere for 24–48 hours. Regarding substrate scope, diverse β,γ-unsaturated ketoximes and *N*-arylacrylamides were compatible with the transformation. Various substituents on the phenyl ring of ketoximes, including both electron-donating and electron-withdrawing groups, were well tolerated, affording the desired products in satisfactory to high yields. Additionally, thiophene-substituted ketoxime and aliphatic ketoxime also participated effectively in the reaction to afford products **9f** and **9e**. Notably, *N*-arylacrylamides bearing different substituents, particularly at the *para*- and *ortho*-positions of the phenyl ring, were well tolerated, although *ortho*-substituents induced steric hindrance effects that led to lower yields.

**Scheme 6:**
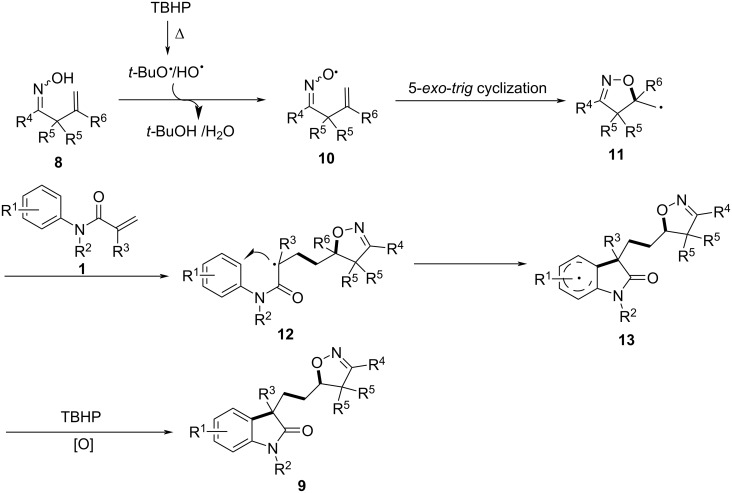
Proposed mechanism for the iminoxyl radical-promoted cascade oxyalkylation/alkylarylation of alkenes.

To gain further insight into the mechanism, several control experiments were performed. The results indicated that an iminoxyl radical is generated as the initiator of the reaction (Scheme 6). Initially, TBHP undergoes homolytic cleavage to generate *t*-BuO and OH radicals. The *t*-BuO radical then abstracts a hydrogen atom from the β,γ-unsaturated ketoxime, forming the iminoxyl radical **10**. This is followed by a 5-*exo-trig* cyclization, yielding a C-centered radical **11**, which then adds to the alkene moiety of the *N*-arylacrylamide, forming intermediate **12**. Subsequent intramolecular cyclization and oxidative aromatization lead to the final isoxazoline-featured oxindole **9**.

**Scheme 7:**
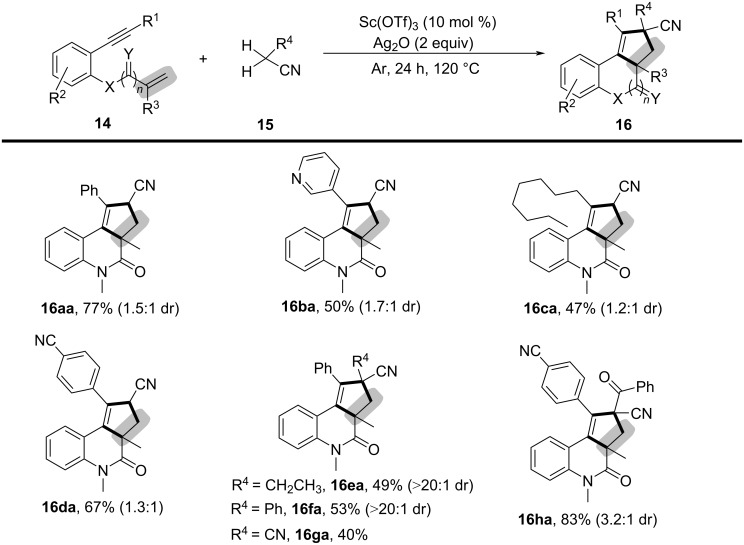
Bicyclization of 1,*n*-enynes with alkyl nitriles.

In 2017, Li’s group reported an oxidative divergent bicyclization of 1,*n*-enynes through α-C(sp^3^)–H functionalization of alkyl nitriles using a Sc(OTf)_3_ and Ag_2_O system (Scheme 7) [6]. This methodology enabled the selective functionalization of one or two C(sp^3^)–H bonds adjacent to the nitrile group, leading to the formation of diverse polycycles with high selectivity. Various reaction parameters were systematically examined, including Lewis acids, oxidants, bases, reaction temperature, and solvents. The optimal conditions were identified as using 10 mol % Sc(OTf)_3_ and 2 equivalents of Ag_2_O at 120 °C under an argon atmosphere. The substrate scope was then evaluated using various *o-*alkynylarylacrylamides and alkyl nitriles under the optimized reaction conditions. Substrates with different substituents, including aryl, heteroaryl, and alkyl groups at the terminal alkyne, were well tolerated, yielding moderate to good results (**16aa**–**da**). Furthermore, variations in the nitrile reaction partners, such as butyronitrile (**16ea**), 2-phenylacetonitrile (**16fa**), malononitrile (**16ga**), and 3-oxo-3-phenylpropanenitrile (**16ha**), were successfully employed to construct the target polycycles.

**Scheme 8:**
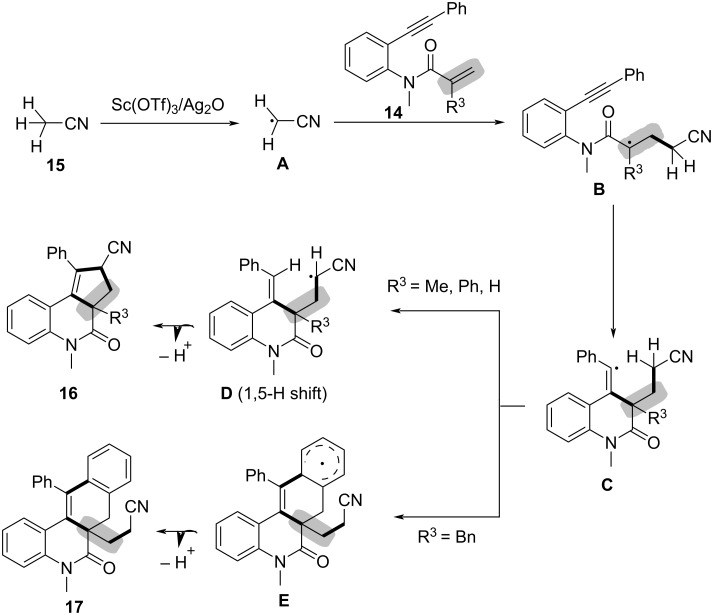
Possible reaction mechanism for the bicyclization of 1,*n*-enynes with alkyl nitriles.

To verify the reaction mechanism, a series of control experiments were conducted. The complete inhibition of the reaction by radical scavengers such as TEMPO, BHT, and hydroquinone suggested a radical-mediated process. Based on the experimental results, a detailed reaction mechanism was proposed (Scheme 8). The reaction begins with the oxidative cleavage of an α-C(sp^3^)–H bond in acetonitrile (**15**) by the Sc(OTf)_3_/Ag_2_O system, generating an alkyl radical **A** through a single-electron-transfer (SET) process. This alkyl radical then adds across the C–C double bond in the enyne, forming an alkyl radical intermediate **B**, which reacts with the C–C triple bond to generate a vinyl radical intermediate **C**. Depending on the substitution effect at the 3-position of the acrylamide moiety, the intermediate undergoes either a 1,5-hydride shift to give **D** or a direct cyclization with the aryl ring via intermediate **E**, which upon deprotonation lead to the final products **16** and **17**.

**Scheme 9:**
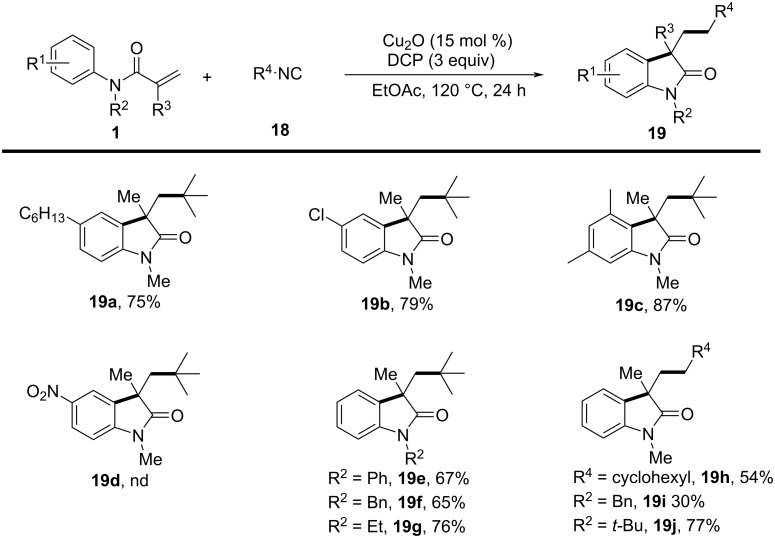
Radical cyclization of *N*-arylacrylamides with isocyanides.

In a 2016 study by Van der Eycken’s group (Scheme 9), an innovative copper-catalyzed alkylarylation of activated alkenes using isocyanides as the alkyl source was presented, providing a novel and efficient route to 3,3-dialkylated oxindoles [7]. In this system, Cu_2_O was used as the catalyst, combined with dicumyl peroxide (DCP) as the oxidant, in ethyl acetate (EtOAc) at 120 °C under an argon atmosphere. This reaction efficiently produced 3,3-dialkylated oxindoles in moderate to high yields. The substrate scope was extensively evaluated, and a variety of *N*-arylacrylamides with different electronic properties and steric demands were successfully converted into the corresponding oxindoles. Substrates with both electron-donating and electron-withdrawing groups on the aromatic ring were well tolerated (**19a**–**c**), although substrates with strong electron-withdrawing groups, such as nitro (–NO₂), resulted in no product (**19d**). Additionally, alkyl, phenyl, and benzyl substituents on the nitrogen atom of *N*-arylacrylamides were compatible (**19e**–**g**), further demonstrating the method’s versatility. Furthermore, a range of isocyanide derivatives, encompassing primary, secondary, and tertiary aliphatic isocyanides, demonstrated compatibility by producing the desired oxindoles **19h**–**j** with moderate to good yields.

**Scheme 10:**
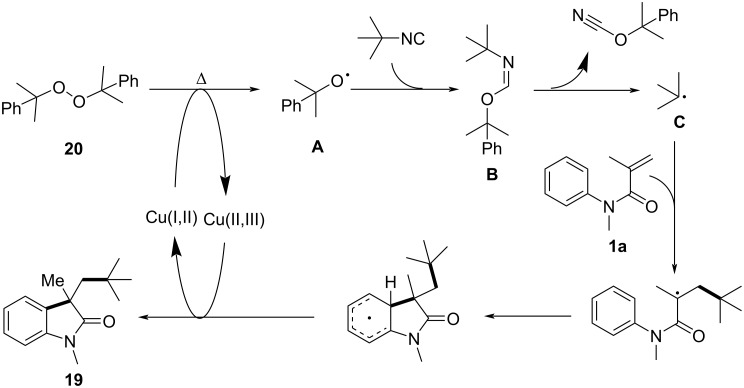
Plausible mechanism for the radical cyclization of *N*-arylacrylamides with isocyanides.

To investigate the reaction mechanism, control experiments were conducted. The addition of radical scavengers such as BHT and TEMPO significantly inhibited the reaction, confirming the involvement of a radical intermediate. Kinetic isotope effect (KIE) studies showed a KIE of 1.0, suggesting that C–H-bond cleavage was not the rate-determining step. Based on these results, a detailed mechanism was proposed (Scheme 10) in which a copper-assisted homolysis of DCP generates a cumyloxyl radical **A**, which initiates the formation of an imidoyl radical **B** from the isocyanide. This radical then undergoes homolytic cleavage to yield an alkyl radical **C**, which adds to the *N*-arylacrylamide, followed by intramolecular cyclization and deprotonation to form the final products **19**.

**Scheme 11:**
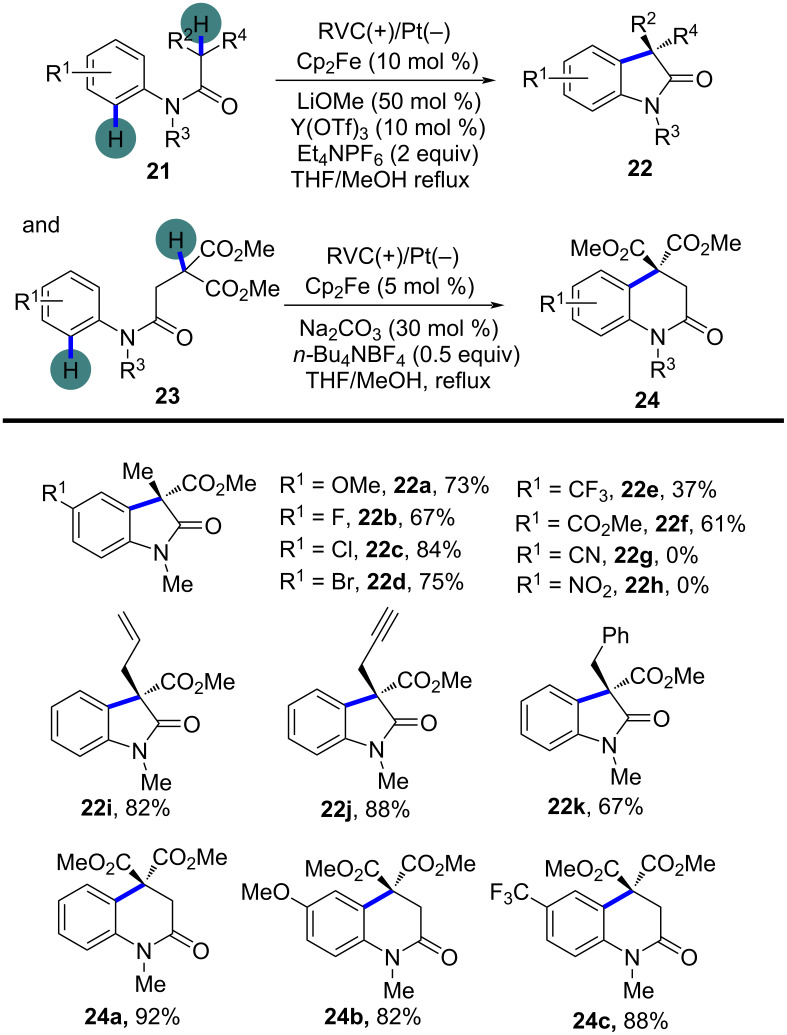
Electrochemical dehydrogenative cyclization of 1,3-dicarbonyl compounds.

To further expand the radical difunctionalization toolbox, electrochemical approaches have emerged as a green and efficient alternative, offering precise redox control without the need for external oxidants. In 2018, Xu’s group reported an electrochemical dehydrogenative cyclization of 1,3-dicarbonyl compounds (Scheme 11) [8]. The study focused on the electrochemical dehydrogenative cyclization of 1,3-dicarbonyl compounds through intramolecular C(sp^3^)–H/C(sp^2^)–H cross-coupling, using Cp_2_Fe-catalyzed electrochemical oxidation. This method leveraged the selective activation of the acidic α-C–H bond within the 1,3-dicarbonyl moiety to generate a carbon-centered radical, which was crucial for the subsequent cyclization. The reaction was carried out under reflux conditions in a THF/MeOH solvent mixture, using a constant current electrolysis setup with a reticulated vitreous carbon (RVC) anode and a platinum cathode. The substrate scope was extensively investigated, and a broad array of *N*-aryl substituents was well tolerated, including electron-donating motifs such as methoxy (-OMe) and halogens (-F, -Cl, -Br), as well as electron-withdrawing functionalities like trifluoromethyl (-CF₃) and ester groups (-CO₂Me). However, highly electron-deficient substrates, such as those bearing cyano (-CN) or nitro (-NO₂) groups, did not react. Additionally, the study explored different alkyl substituents at the α-position of the 1,3-dicarbonyl compounds, including functionalized alkyl chains with alkenyl, alkynyl, and aryl groups, all of which were compatible with the reaction conditions. Interestingly, when α-alkylmalonic esters were employed instead of malonate amide moieties, the 3,4-dihydro-1*H*-quinolin-2-one compounds were obtained in good to excellent yields.

**Scheme 12:**
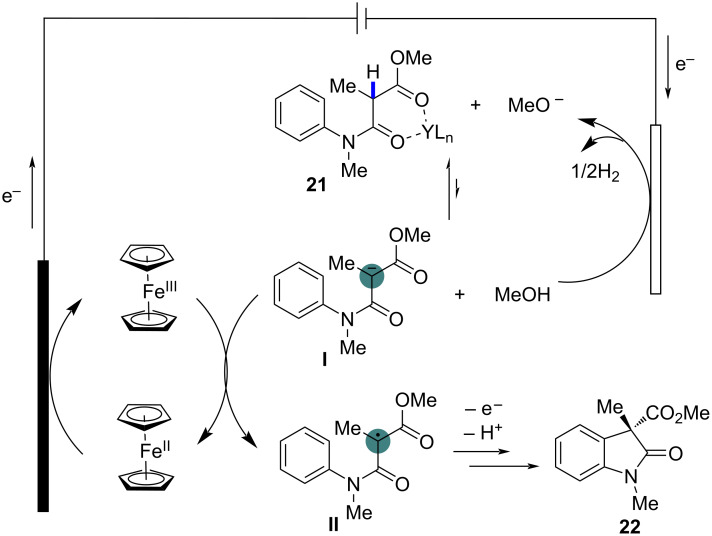
Plausible mechanism for the dehydrogenative cyclization of 1,3-dicarbonyl compounds.

To gain further insights into the reaction mechanism, control experiments were conducted. The reaction was significantly inhibited by radical scavengers such as TEMPO, BHT, and hydroquinone, strongly suggesting a radical-mediated process. The necessity of Cp_2_Fe and Y(OTf)_3_ for the reaction was also confirmed, as the absence of either catalyst led to a dramatic decrease in product yield. These findings highlighted the critical role of electrochemically generated carbon-centered radicals in driving the cyclization. Based on these results and previous studies, a detailed reaction mechanism was proposed as shown in Scheme 12. The process begins with the oxidation of Cp_2_Fe at the anode to form Cp_2_Fe^+^, which then facilitates the deprotonation of the α-C–H bond of the 1,3-dicarbonyl compound by methoxide, generated at the cathode. This deprotonation leads to the formation of a carbanion, which undergoes single-electron transfer (SET) with Cp_2_Fe^+^, resulting in the generation of a carbon-centered radical. This radical subsequently undergoes intramolecular cyclization with the aryl ring to form the final oxindole or quinolinone products.

**Scheme 13:**
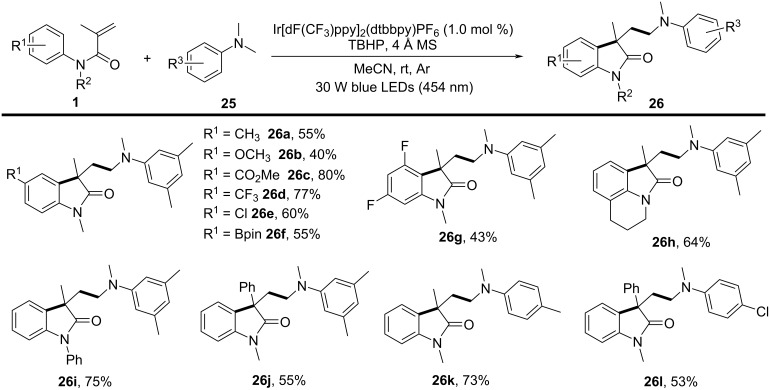
Photocatalyzed cyclization of *N*-arylacrylamide and *N*,*N*-dimethylaniline.

Beyond electrochemical protocols, photochemical activation – particularly visible-light photoredox catalysis – has become a powerful and sustainable strategy for generating carbon radicals under mild conditions. In 2023, Fan’s group discovered a radical cyclization of *N*-arylacrylamides with α-aminoalkyl radicals generated from tertiary arylamines using photoredox catalysis (Scheme 13) [9]. In this system, Ir[dF(CF_3_)ppy]_2_(dtbbpy)PF_6_ was used as a photosensitizer to trigger the α-C–H activation of *N*,*N*-dimethylaniline, generating an alkyl radical under 30 W blue LED (454 nm) irradiation. Notably, an equivalent amount of TBHP was required as an oxidant to regenerate the photocatalyst. Various substituents, including electron-donating and electron-withdrawing groups on the aryl ring of *N*-arylacrylamides, such as *p*-Me, *p*-MeO, *p*-MeO_2_C, *p*-CF₃, *p*-Cl, and *p*-pinB, were compatible with this transformation, yielding the corresponding products **26a**–**f** in moderate to good yields. Moreover, a 3,5-difluoro-substituted arylacrylamide was also converted to the desired product **26g** in 43% yield. The study also explored *N*-linked substituents, and both aryl and alkyl groups were compatible, smoothly providing the target products **26h**, **26i**. Additionally, replacing the methyl group on the acrylamide olefin with a phenyl group resulted in a 55% yield of product **26j**. Focusing on *N*,*N*-dimethylanilines, -CH_3_ and -Cl substituents at the *para*-position of dimethylanilines were amenable to the system, yielding the final products **26k** and **26l** in 73% and 53% yield, respectively.

**Scheme 14:**
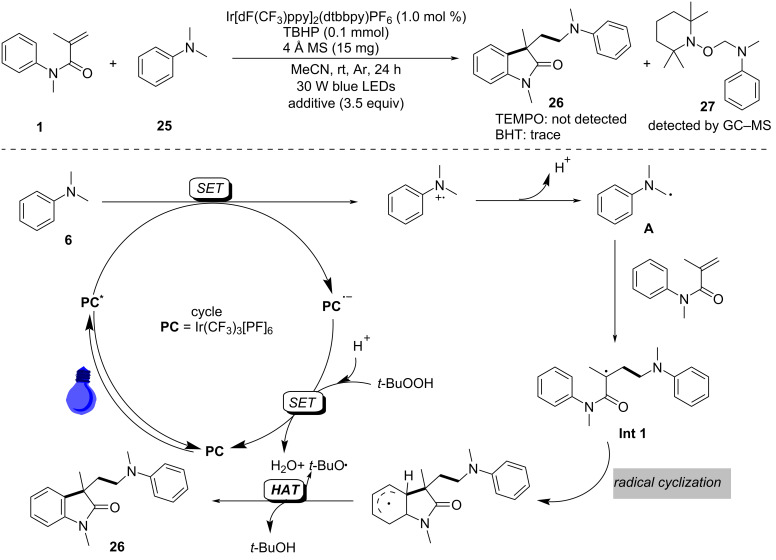
Proposed mechanism for the photocatalyzed cyclization of *N*-arylacrylamides and *N*,*N*-dimethylanilines.

Radical-trapping experiments with TEMPO (2,2,6,6-tetramethylpiperidine-1-oxyl) and BHT (butylated hydroxytoluene) additives confirmed the involvement of α-aminoalkyl radicals in this transformation, as shown in Scheme 14. A single-electron-transfer (SET) process occurred efficiently under blue LED irradiation in the presence of Ir[dF(CF_3_)ppy]_2_(dtbbpy)PF_6_ as the photocatalyst. This was followed by deprotonation and radical migration, yielding α-aminoalkyl radical **A**, which added to the intramolecular C=C bond of *N*,*N*-dimethylaniline to produce alkyl radical intermediate **1**. Subsequently, radical cyclization and deprotonation, assisted by the *ter*t-butoxy radical, led to the desired products **26**. It is important to emphasize that TBHP plays a crucial role as an oxidant in regenerating the photocatalyst for the catalytic cycle.

**Scheme 15:**
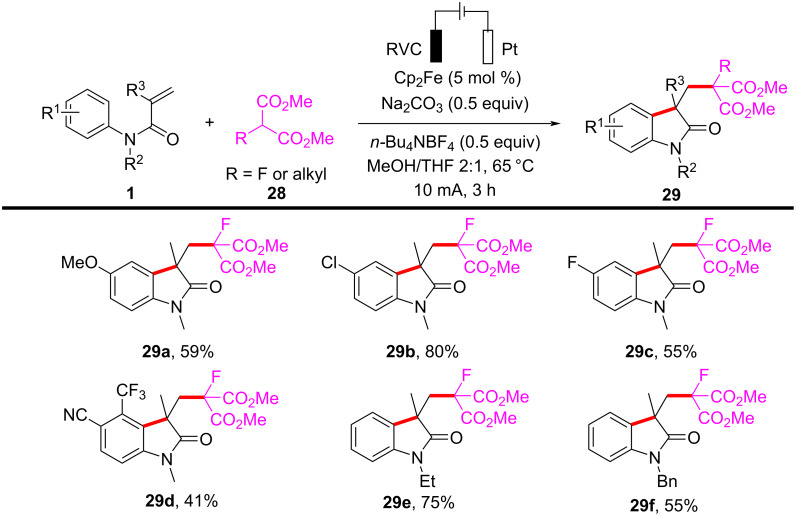
Electrochemical monofluoroalkylation cyclization of *N*-arylacrylamides with dimethyl 2-fluoromalonate.

In the same year, Wang and his group reported an electrochemically induced intramolecular radical cyclization of *N*-arylacrylamides with dimethyl 2-fluoromalonate as a monofluoroalkyl radical precursor, affording fluorinated 2-oxindoles in synthetically useful yields (Scheme 15) [10]. In this system, catalytic Cp_2_Fe was employed as a redox catalyst, Na_2_CO_3_ as a base, and *n*-Bu_4_NBF_4_ as the electrolyte, all participating in an undivided electrolytic cell with a reticulated vitreous carbon (RVC) anode and a platinum (Pt) cathode at a constant current of 10 mA, in a co-solvent mixture of MeOH/THF 2:1 for 3 hours to deliver the target cyclization products. A variety of substituents on the aromatic rings of *N*-arylacrylamides, including -OCH_3_, -F, -Cl, -CN, and -CF_3_, were compatible, yielding the corresponding products **29a–d**. Additionally, *N*-linked alkyl groups, such as -Et and -Bn, were also suitable for this transformation, affording the products **29e** and **29f** in quantitative yields.

**Scheme 16:**
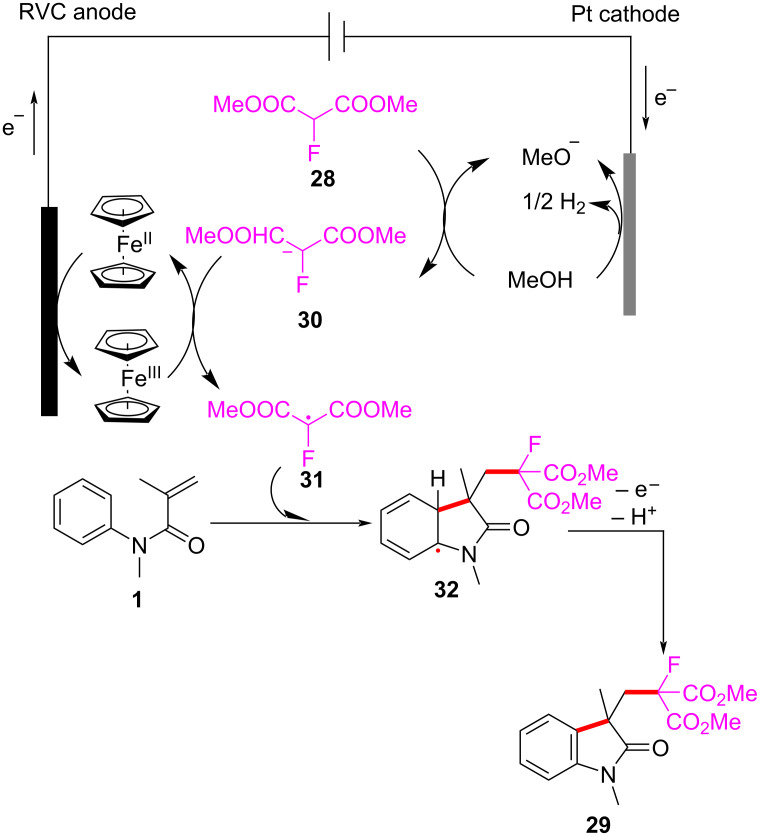
Proposed mechanism for the electrochemical radical cyclization of *N*-arylacrylamides with dimethyl 2-fluoromalonate.

A plausible mechanism for the electrochemically induced radical cyclization is proposed in Scheme 16. Due to its lower oxidation potential, Cp_2_Fe(II) is first oxidized to Cp_2_Fe(III), which then oxidizes the carbanion intermediate **30** generated from the starting material **28** to yield an alkyl radical intermediate **31**. This radical undergoes C=C-bond addition, cyclization, and deprotonation, ultimately leading to the formation of the desired products. Simultaneously, MeOH is reduced at the cathode to release H_2_, maintaining the electronic balance of the system.

**Scheme 17:**
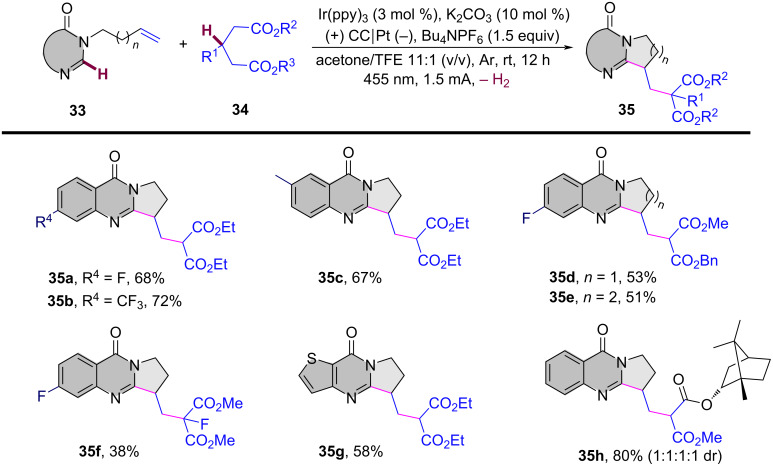
Photoelectrocatalytic carbocyclization of unactivated alkenes using simple malonates.

In 2024, Liang’s group reported the replacement of *N*-arylacrylamides with 7-fluoro-3-homoallylquinazolin-4-ones, which are tethered to an alkenyl group at the nitrogen atom and function as radical acceptors (Scheme 17). This approach effectively facilitated photoelectrocatalytic carbocyclization with diethyl malonate, yielding fluorinated pyrroloquinazolinones in acceptable to high yields [11]. The reaction was conducted in an undivided cell with a carbon cloth anode and a platinum (Pt) cathode, using K_2_CO_3_ (10 mol %) as the base, Ir(ppy)_3_ (3 mol %) as the photocatalyst, and an equivalent amount of Bu_4_NPF_6_ as the supporting electrolyte in a MeOH/TFE 11:1 (v/v) co-solvent mixture under blue LED irradiation at a constant current of 1.5 mA for 12 hours at room temperature. A wide range of substrates exhibited good functional group tolerance, with 3-homoallylquinazolin-4-ones bearing electron-withdrawing or electron-donating substituents at the 6- (**35a**, **35b**) and 7- (**35c**) positions, yielding pyrroloquinazolinones in 67–72% yields. Additionally, another heteroaromatic quinazolinone derivative **35g** was obtained in satisfactory yield. In terms of malonates, asymmetric C–H radical precursors, including benzyl methyl malonates (**35d**, **35e**) and a malonate derived from (−)-borneol (**35h**), were all compatible with the reaction conditions, providing the final products in moderate to good yields. However, using the sterically hindered 2-fluoromalonate as a substrate led to a reduced yield of product **35f**.

**Scheme 18:**
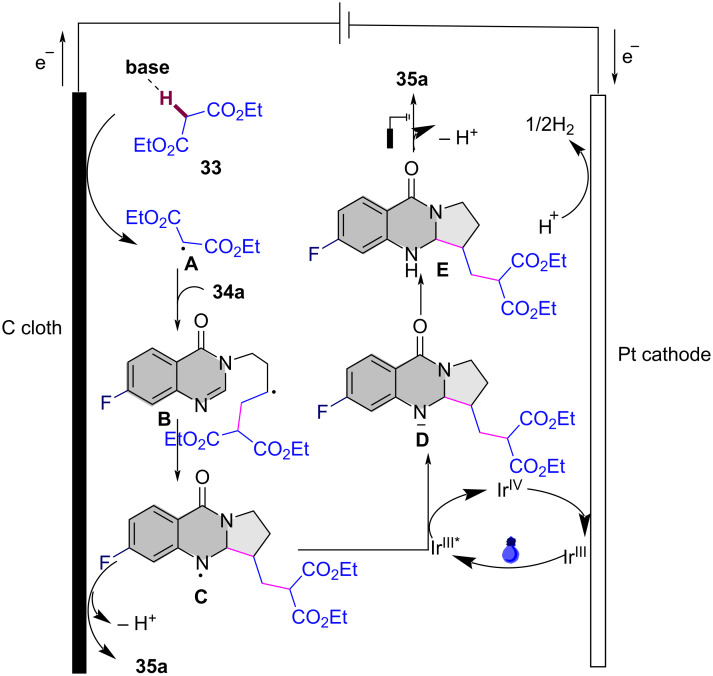
Plausible mechanism for the photoelectrocatalytic carbocyclization of unactivated alkenes with simple malonates.

A plausible mechanism for the electrochemical radical cyclization is proposed in Scheme 18. Initially, a diethyl malonate radical **A** is generated through a proton-coupled electron-transfer (PCET) process at the anode, with the aid of K_2_CO_3_. This radical then adds to the C=C bond, forming alkyl radical intermediate **B**. Intermediate **B** undergoes cyclization to form the *N*-radical intermediate **C**, which can be further oxidized to the final product **35a** by releasing a proton. Alternatively, intermediate **C** can be reduced by excited-state Ir(ppy)_3_, leading to the formation of the nitranion intermediate **D**. The Ir(III) catalyst is then regenerated at the Pt cathode. Protonation of **D** gives intermediate **E**, and subsequent deprotonation at the anode yields the target compound **35a**.

### *N*-Arylalkenes: difluoroalkyl/trifluoroalkyl radicals

**Scheme 19:**
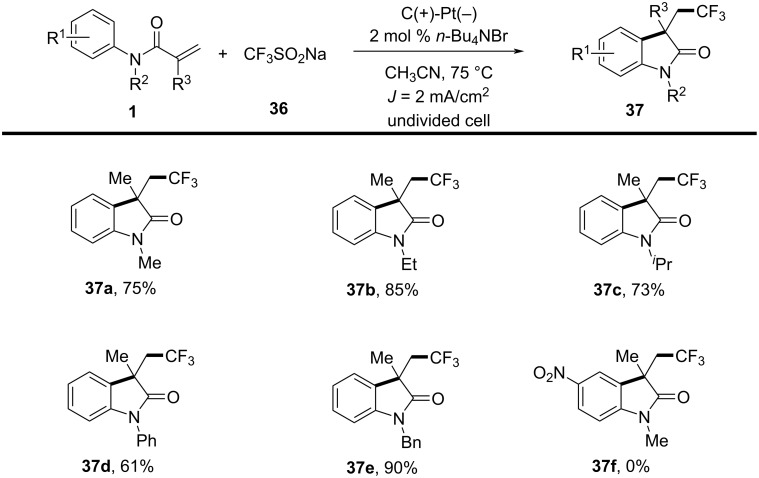
Bromide-catalyzed electrochemical trifluoromethylation/cyclization of *N*-arylacrylamides.

While the majority of radical cyclizations involve hydrocarbon-based radicals, the incorporation of fluorinated motifs such as trifluoromethyl and difluoroalkyl groups offers unique physicochemical properties, and has become a burgeoning area of research. In 2018, Zeng’s group reported a novel electrochemical trifluoromethylation/cyclization reaction of *N*-arylacrylamides catalyzed by bromide ions, making a significant contribution to synthetic methodology (Scheme 19) [12]. This method was conducted in an undivided cell, using *N*-methyl-*N*-phenylacrylamide **1** and sodium trifluoromethanesulfinate (**36**) as model reactants. Through meticulous optimization of conditions, including catalyst selection, solvents, and current density, the best results were obtained using tetrabutylammonium bromide as the catalyst in CH₃CN, with a low catalyst loading of 2 mol %, and palladium wire as the cathode, yielding the target compound in 81% yield. This study capitalized on the use of bromide ions as redox catalysts for generating trifluoromethyl radicals, thereby eliminating the need for expensive transition-metal catalysts and external oxidants, which enhances both the economic and environmental benefits of the process. Furthermore, the research demonstrated the method's broad applicability across various *N*-substituted acrylamides. Compounds such as *N*-methyl, *N*-ethyl, *N*-isopropyl, *N*-phenyl, and *N*-benzyl-substituted acrylamides were efficiently transformed into products **37a**–**e** under the optimized conditions. However, substrates with strong electron-withdrawing groups, such as those bearing nitro substituents (**37f**), failed to react effectively.

**Scheme 20:**
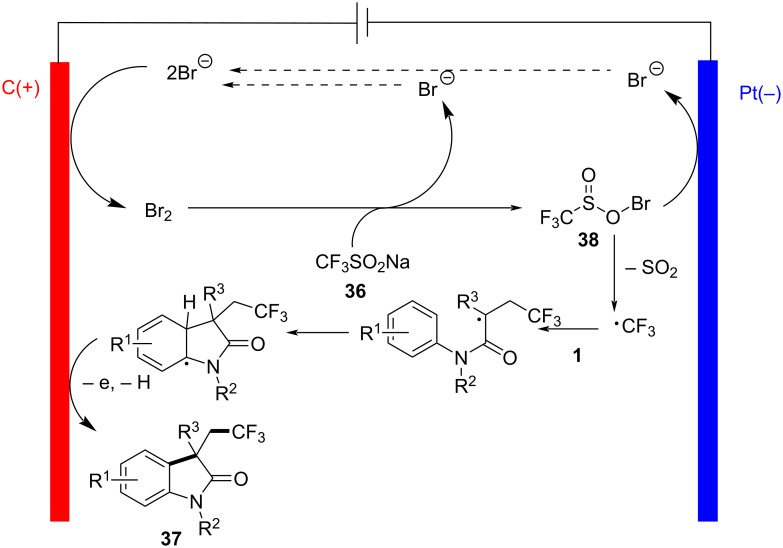
Proposed mechanism for the electrochemical trifluoromethylation/cyclization of *N*-arylacrylamides.

Additionally, the detailed reaction mechanism, explored through control experiments and cyclic voltammetry analysis, revealed a complex series of chemical transformations. The proposed mechanism involves the anodic oxidation of bromide ions to generate molecular Br_2_, which then reacts with sodium trifluoromethanesulfinate (**36**) to form a sulfonyl hypobromite intermediate **38** (Scheme 20). This intermediate undergoes reduction at the cathode, leading to the formation of oxygen-centered and sulfonyl-centered radicals, which subsequently form a trifluoromethyl radical. This radical then reacts with acrylamide to yield the desired product **37**.

**Scheme 21:**
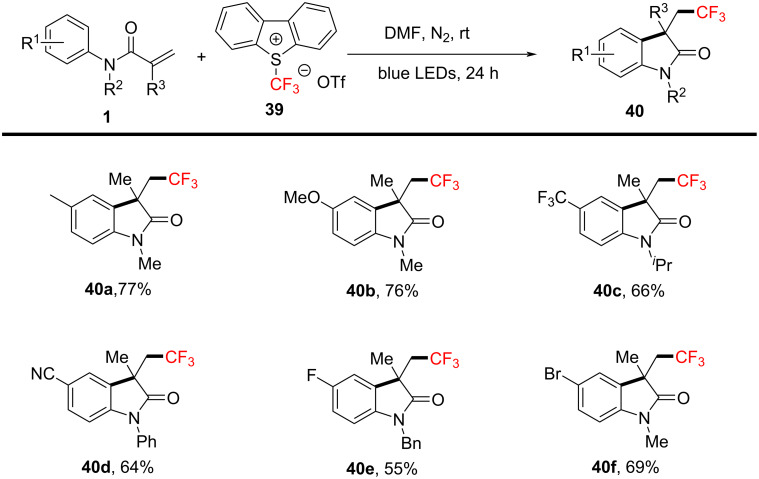
Visible light-mediated trifluoromethylarylation of *N*-arylacrylamides.

As shown in Scheme 21, a visible light-induced trifluoromethylation/arylation using Umemoto’s reagent for the synthesis of trifluoromethylated oxindole derivatives was reported by Huang and Zhang’s group in 2021 [13]. This method is particularly noteworthy for its use at room temperature, requiring no transition metals, photocatalysts, or additives. Notably, Umemoto's reagent served as the trifluoromethyl source, and the reaction was facilitated under blue LED irradiation, achieving good to excellent yields. Moreover, this approach simplified the reaction system, eliminating the need for expensive or toxic materials, and making the methodology environmentally friendly and user-friendly, thus enhancing its practical applicability. Additionally, a variety of *N*-arylacrylamides, featuring either electron-donating or electron-withdrawing groups on the aromatic ring (**40a**–**d**), effectively underwent this transformation. Halogen substituents (**40e**, **40f**) on the substrates also demonstrated excellent reaction efficiency and substrate tolerance, further broadening the potential scope of applications.

**Scheme 22:**
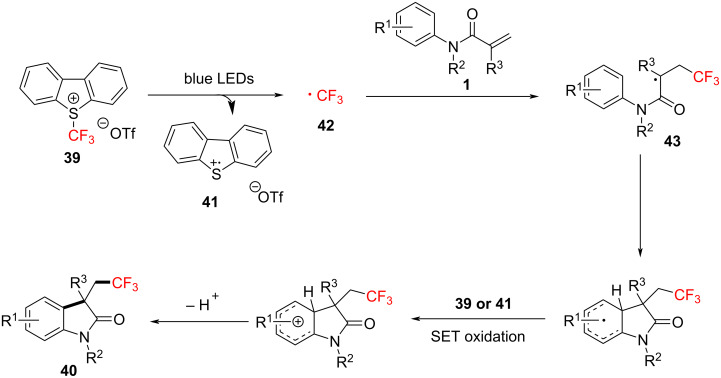
Plausible reaction mechanism for the visible light-mediated trifluoromethylarylation of *N*-arylacrylamides.

Regarding the reaction mechanism (Scheme 22), the process involves the homolytic cleavage of Umemoto's reagent **39** under visible light irradiation, releasing a trifluoromethyl radical **42**. The radical then adds to the double bond of *N*-arylacrylamide, forming intermediate radical **43**. Subsequently, this intermediate undergoes intramolecular cyclization and deprotonation to yield the desired cyclic product **40**, with Umemoto’s reagent serving both as the trifluoromethyl source and the oxidant.

**Scheme 23:**
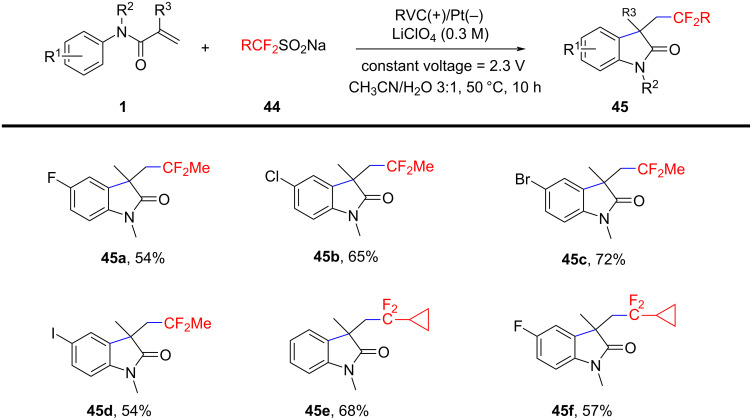
Electrochemical difluoroethylation cyclization of *N*-arylacrylamides with sodium difluoroethylsulfinate.

**Scheme 24:**
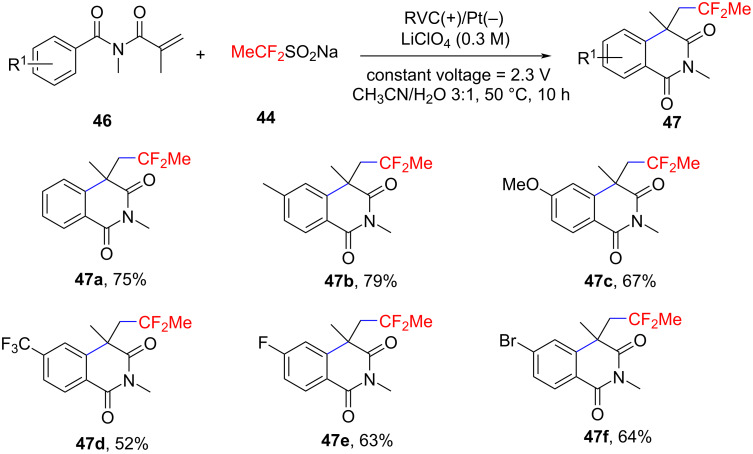
Electrochemical difluoroethylation cyclization of *N*-methyacryloyl-*N*-alkylbenzamides with sodium difluoroethylsulfinate.

In 2023, Li and Fu’s group reported a novel metal-free electrochemical oxidative difluoroethylation method for constructing azaheterocycles, using sodium difluoroethylsulfinate (DFES-Na) to generate difluoroethyl radicals via anodic single-electron oxidation (Scheme 23) [14]. The method was evaluated with various *N*-arylacrylamides, revealing that substrates with different substituents, including fluorine, chlorine, bromine, and iodine, participated well in the reaction, with yields of products **45a–d** ranging from 54% to 72%. Additionally, the method was successfully applied to synthesize cyclopropyldifluoromethylated oxindoles **45e**, **45f** and difluoroethylated isoquinoline-1,3-diones **47a**–**f** (Scheme 24), demonstrating high functional group compatibility. Moreover, control experiments and cyclic voltammetry (CV) indicated that the reaction mechanism proceeds via a radical pathway. DFES-Na undergoes single-electron oxidation at the anode, generating a radical that reacts with the olefinic amide, followed by intramolecular cyclization to form the target difluoroalkylated azaheterocycles.

**Scheme 25:**
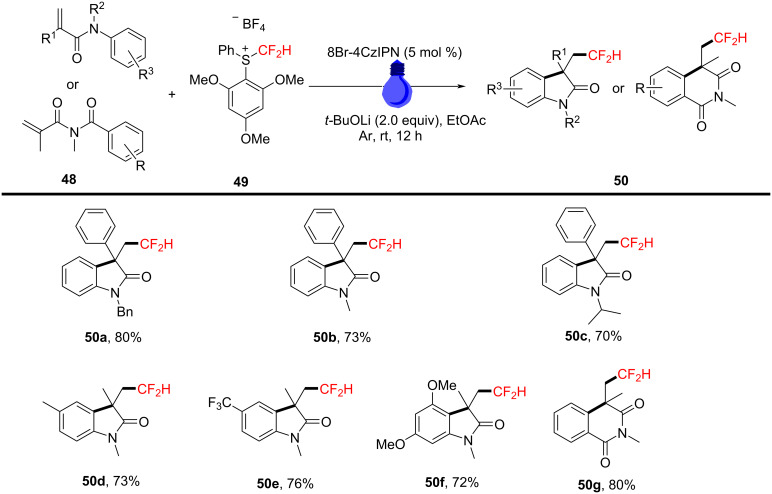
Photoredox-catalyzed radical aryldifluoromethylation of *N*-arylacrylamides with *S*-(difluoromethyl)sulfonium salt.

In 2023, Liu’s group reported a photoredox-catalyzed reaction under visible light using S-(difluoromethyl)sulfonium salt **49** as an effective difluoromethyl radical precursor (Scheme 25). The reaction was mediated by blue LED light and various organic photosensitizers, such as 4CzIPN and 8Br-4CzIPN, to facilitate the aryldifluoromethylation of *N*-arylacrylamides. Experiments demonstrated that using 8Br-4CzIPN as the photocatalyst and tetrabutylammonium hydroxide as the base in ethyl acetate yielded the best results [15]. A variety of substrates were tested to assess the generality of the reaction. The results indicated that the *N*-protecting group had little impact on the reaction’s efficiency, and various substrates (e.g., methyl, benzyl, isopropyl) were effectively converted to their respective target products **50a**–**c**. Moreover, benzene rings bearing different substituents, whether electron-withdrawing or electron-donating, and polysubstituted substrates, showed good tolerance under the standard conditions (**50d**–**f**). When an *N*-acryloyl-*N*-methylbenzamide was employed as substrate, the desired compound **50g** was obtained in 80% yield.

**Scheme 26:**
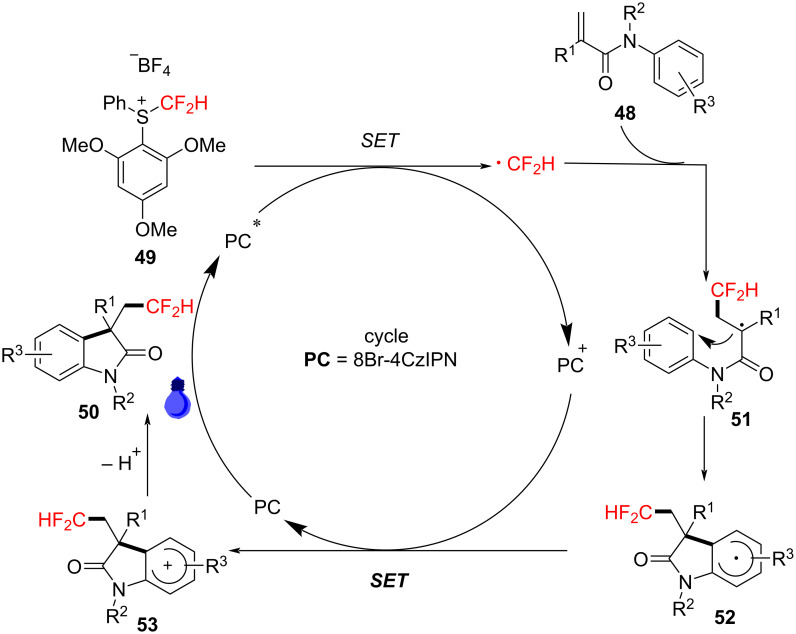
Proposed mechanism for the photoredox-catalyzed radical aryldifluoromethylation of *N*-arylacrylamides with *S*-(difluoromethyl)sulfonium salt.

Through control experiments and mechanistic studies, a plausible reaction mechanism was proposed (Scheme 26). The process begins with the generation of a difluoromethyl radical from S-(difluoromethyl)sulfonium salt **49** under the influence of an excited-state photocatalyst. This radical then adds to the double bond of *N*-arylacrylamide, forming intermediate radical **51**. Subsequently, this intermediate undergoes intramolecular cyclization and deprotonation, yielding the final product **50**.

**Scheme 27:**
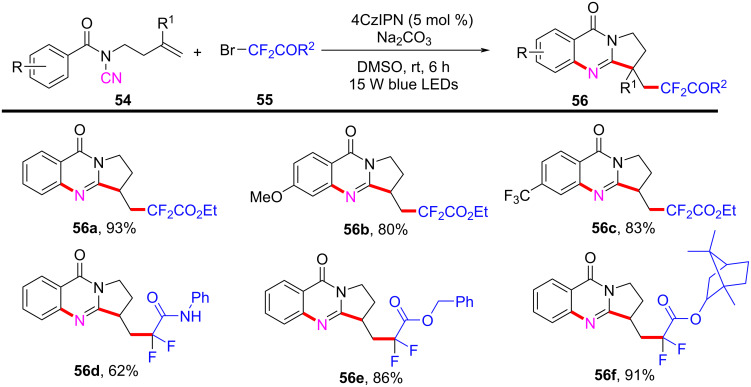
Visible-light-induced domino difluoroalkylation/cyclization of *N***-**cyanamide alkenes.

In 2024, Cui’s group introduced a novel visible-light-induced domino difluoroalkylation/cyclization reaction, providing an efficient method for synthesizing CF₂COR-containing quinazolinones from *N*-cyanamide alkenes (Scheme 27) [16]. This approach is notable for its alignment with the principles of green chemistry, utilizing the metal-free photocatalyst 4CzIPN under visible light conditions. In this system, *N*-cyanamide alkene **54** and BrCF_2_CO_2_Et **55** were used as model substrates, with 4CzIPN as the photocatalyst (Scheme 27). The reaction was carried out under blue LED irradiation in DMSO as the solvent, yielding valuable polycyclic quinazolinones in satisfactory yields. Furthermore, a variety of substituents on the phenyl ring of *N*-cyanamide alkenes, as well as a series of difluorinated reagents, were tested, ranging from electron-donating to electron-withdrawing groups. These substrates consistently achieved moderate to excellent yields, demonstrating high reaction efficiency of the catalytic system under the standard conditions.

**Scheme 28:**
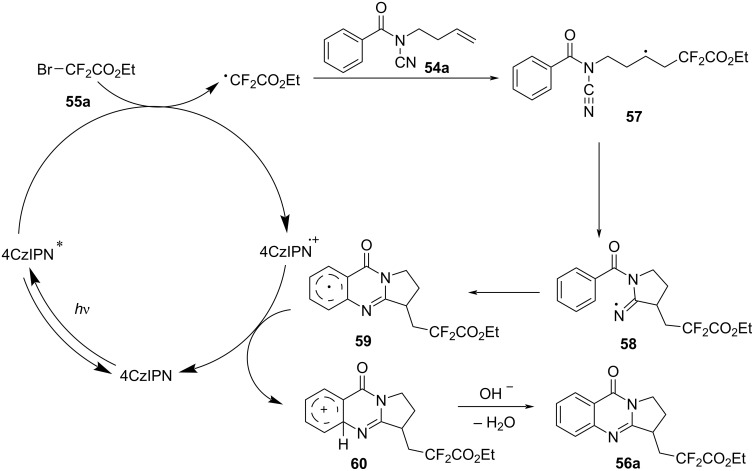
Proposed mechanism of photoredox-catalyzed radical domino difluoroalkylation/cyclization of *N*-cyanamide alkenes.

The reaction mechanism was investigated through controlled experiments, which suggested that the transformation proceeds via a radical pathway. The proposed mechanism begins with the excitation of 4CzIPN by visible light, followed by a single-electron transfer that generates a radical intermediate (Scheme 28). A cascade of bond-forming reactions then occurs, leading to the formation of radical annulation species **59**. Single-electron oxidation and deprotonation then take place, resulting in the final cyclized products **56a**.

### *N*-Arylalkenes: α-carbonyl alkyl C(sp^3^)-centered radicals

**Scheme 29:**
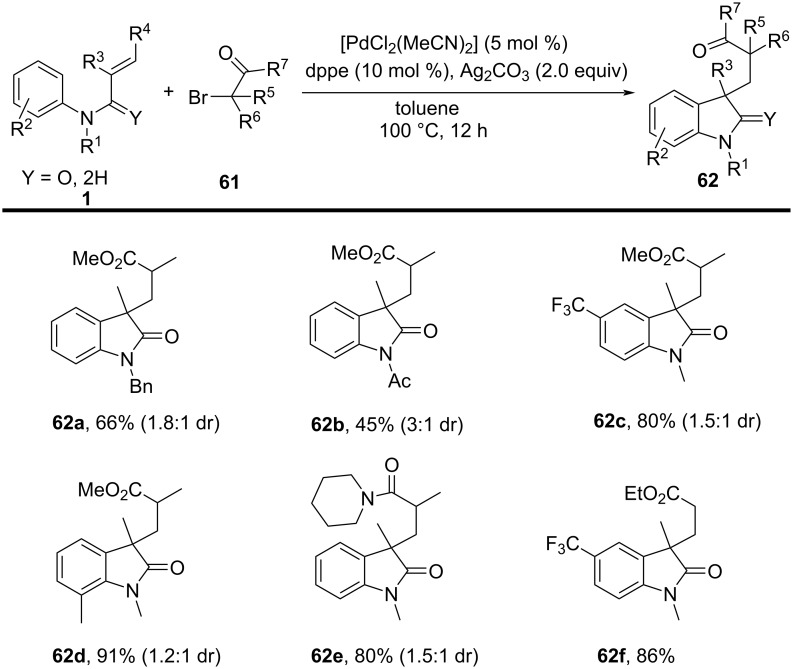
Palladium-catalyzed oxidative difunctionalization of alkenes.

In 2014, Li’s group developed an oxidative difunctionalization of *N*-arylacrylamides with α-carbonylalkyl bromides using palladium (Scheme 29) [17]. This reaction was initiated by a Heck insertion and required Ag_2_CO_3_ as an oxidant. It proceeded via a tandem C–Br/C–H functionalization and cyclization steps, ultimately realizing an oxidative radical pathway. The process exhibited a broad substrate scope and excellent functional-group tolerance (**62a**–**f**).

**Scheme 30:**
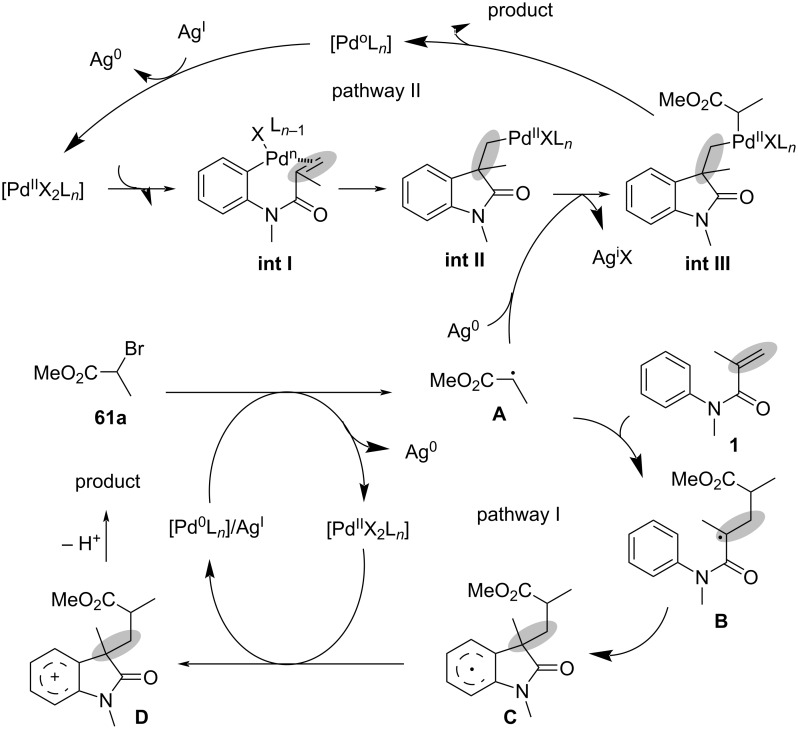
Two possible mechanisms of palladium-catalyzed oxidative difunctionalization.

Based on the experimental results, two possible mechanisms were proposed for this transformation, as illustrated in Scheme 30. In the first mechanism, an alkyl radical **A** is generated in the presence of PdCl_2_(MeCN)_2_ and Ag_2_CO_3_, and then undergoes a tandem sequence of addition to the C=C bond, cyclization, oxidation, and hydrogen abstraction to yield the final product. In the second pathway, a σ-arylpalladium(II) intermediate **I** is formed, initiated by the active [Pd^II^X_2_L*_n_*] species. This intermediate then undergoes a carbopalladation process to generate σ-alkylpalladium(II) intermediate **II**, which is trapped by the α-carbonylalkyl radical **A** in the presence of Ag^0^ species [18]. Finally, reductive elimination occurs to yield the desired product.

**Scheme 31:**
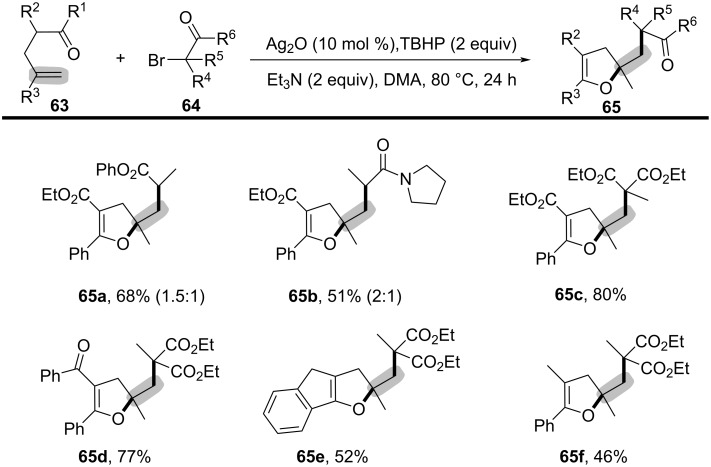
Silver-catalyzed oxidative 1,2-alkyletherification of unactivated alkenes with α-bromoalkylcarbonyl compounds.

Inspired by the above work, a novel, general silver-catalyzed oxidative alkyletherification of olefinic carbonyls with α-bromoalkylcarbonyls promoted by *tert*-butyl hydroperoxide (TBHP) and Et_3_N was developed by Li’s group in 2019 (Scheme 31) [19]. The reaction relied on a Ag-catalyzed concomitant intramolecular annulation process, and presented a broad substrate scope, excellent levels of selectivity, and high functional group compatibility (**65a**–**f**).

**Scheme 32:**
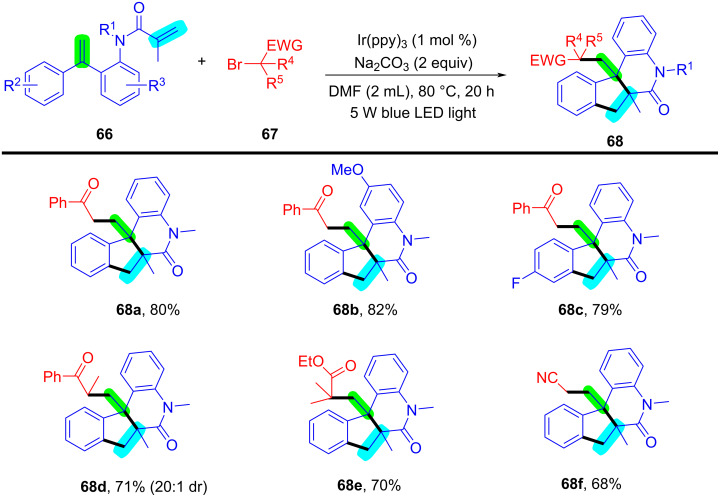
Photochemical radical cascade cyclization of dienes.

In 2024, Huang, Liu, and Li’s group introduced a novel approach for the photochemical radical cascade 6-*endo* cyclization of dienes using α-carbonyl bromides, as illustrated in Scheme 32 [20]. This method is characterized by a visible-light-induced radical cascade cyclization, where the α-carbonyl bromides serve as alkyl radical precursors, enabling the synthesis of complex lactam structures with excellent chemo- and regioselectivity. The system demonstrated a broad substrate scope, encompassing various dienes and α-carbonyl bromides. It showed compatibility with primary, secondary, and tertiary bromides, yielding benzo-fused lactams with good functional group tolerance, thereby highlighting the method’s versatility for the synthesis of diverse bioactive molecules.

**Scheme 33:**
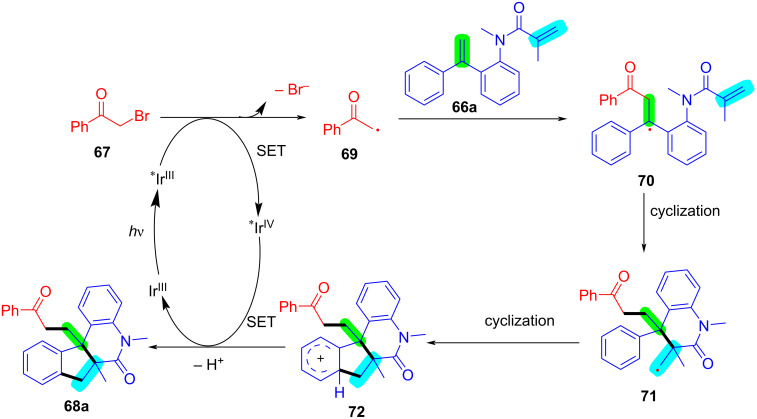
Proposed mechanism for the photochemical radical cascade 6-*endo* cyclization of dienes with α-carbonyl bromides.

The proposed mechanism involves the initial generation of alkyl radical **69** from α-carbonyl bromide **67** through photoreduction by the excited state of the photocatalyst [Ir^III^*] (Scheme 33). The generated radical then undergoes addition to the diene, followed by 6-*endo* cyclization and deprotonation, ultimately forming the lactam product **68a**. Notably, this process highlights the photocatalyst's efficiency in mediating both the initiation and propagation steps of the radical cascade annulation.

**Scheme 34:**
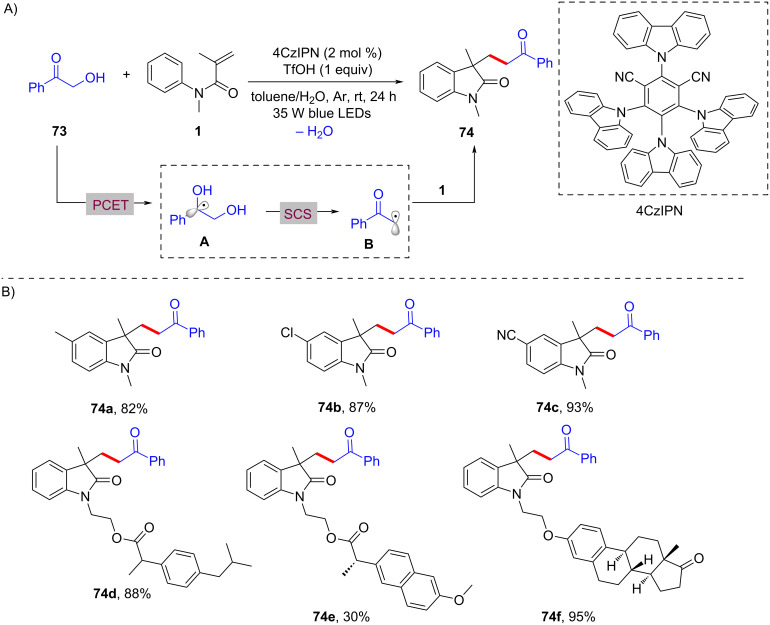
Photocatalyzed radical coupling/cyclization of *N*-arylacrylamides and.

In 2023, Huang and his research group documented a visible-light-induced photoredox PCET/SCS cascade cyclization process to deliver functionalized acyloxindoles with good to excellent yields. In this process, 4CzIPN was employed as the photocatalyst and TfOH as the proton source to ensure the formation of the desired products (Scheme 34) [21]. A range of substituents, including -CH_3_, -Cl, and -CN, on the benzene ring of *N*-arylacrylamides (**74a**–**c**), were compatible with the reaction conditions, exhibiting excellent reaction performance. Moreover, drug-derived *N*-arylacrylamides, such as ibuprofen (**74d**), naproxen (**74e**), and estrone (**74f**), were also effectively applicable to the protocol.

The mechanistic investigation revealed that hydroxyketones **73** are converted into a hydroxyalkyl radical intermediate **A** through a proton-coupled electron-transfer (PCET) process. This is followed by a spin-center shift (SCS) process, leading to the formation of α-carbonyl radical **B**, which adds to the C=C bond of *N*-arylacrylamide **1**. Radical cyclization and deprotonation then occurs to yield the final target products **74**.

**Scheme 35:**
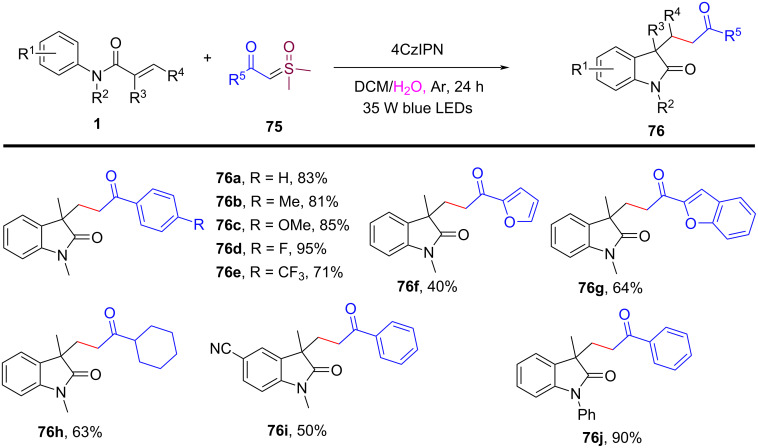
Photocatalyzed radical-type couplings/cyclization of *N*-arylacrylamides with sulfoxonium ylides.

In 2023, Huang’s group demonstrated the visible-light-induced formation of carbon radicals from aqueous sulfoxonium ylides, as shown in Scheme 35 [22]. These ylides, traditionally used in polar chemistry, were found to undergo photochemical redox reactions in the presence of a photocatalyst (4CzIPN), leading to either the reduction of sulfoxonium ylides to hydrocarbons or their participation in radical coupling with alkenes for carboarylation reactions. The reactions were carried out under mild, room temperature conditions using a DCM/H_2_O 1:2 solvent system. Various reaction conditions were optimized, demonstrating broad substrate tolerance. Both electron-donating (e.g., methyl, methoxy) and electron-withdrawing (e.g., halides, trifluoromethyl) groups on the benzoyl sulfoxonium ylides were tolerated, leading to the formation of oxindoles **76a–e** in good to excellent yields of up to 95%. Heteroaryl and aliphatic sulfoxonium ylides also participated in the reaction, though with slightly lower yields of product **76f**–**h**. Furthermore, the scope was extended to other *N*-arylacrylamide derivatives, affording products **76i** and **76j** with yields of 50% and 90%, respectively.

**Scheme 36:**
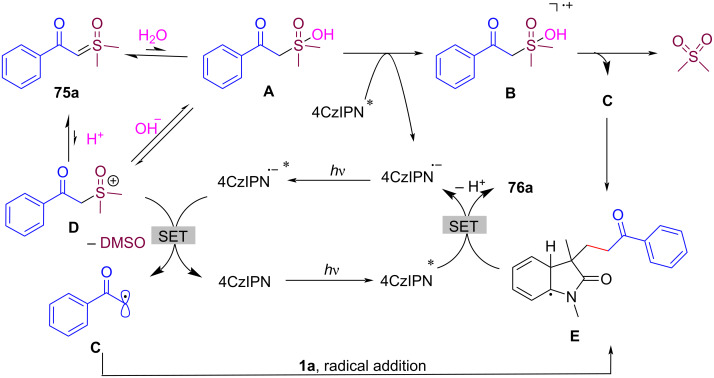
Possible mechanism of visible-light-induced radical-type couplings/cyclization of *N*-arylacrylamides with sulfoxonium ylides.

As shown in Scheme 36, the proposed mechanism involves the excitation of the photocatalyst (4CzIPN), which undergoes a single-electron-transfer (SET) process with sulfoxonium ylides to generate radical intermediate **C**. The generated radical **C** then adds to the C=C bond of *N*-arylacrylamide **1**, followed by radical cyclization and oxidation, ultimately forming the final oxindole product **76a**. Interestingly, the reaction proceeds via two distinct pathways depending on the presence of an alkene radical acceptor, with DMSO or dimethyl sulfone as the byproduct.

**Scheme 37:**
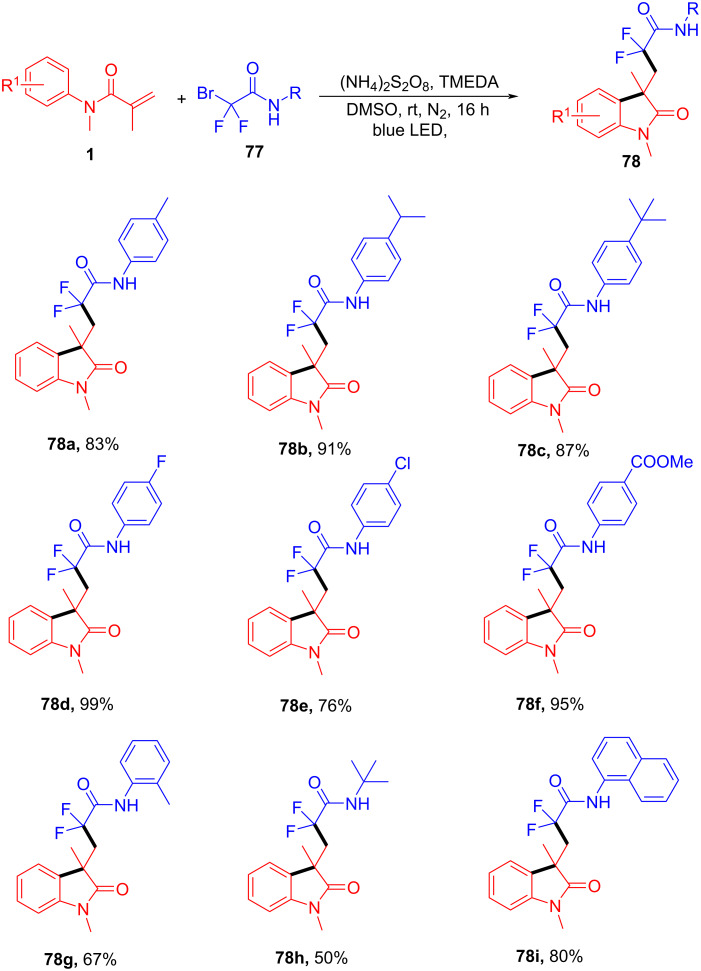
Visible-light-promoted difluoroalkylated oxindoles systhesis via EDA complexes.

In addition to transition-metal and photocatalyzed systems, several metal-free methods utilizing electron donor–acceptor (EDA) complexes and peroxide initiators have recently gained attention due to their operational simplicity and environmental friendliness. In 2024, Song’s group and co-workers presented a novel metal-free, visible-light-promoted method for synthesizing difluoroamidated oxindoles via electron donor–acceptor (EDA) complexes (Scheme 37) [23]. The method involved the use of *N*-phenylacrylamides and bromodifluoroacetamides as starting materials, with *N*,*N,N’,N’-t*etramethylethylenediamine (TMEDA) acting as the electron donor. The reaction proceeded efficiently under mild conditions, utilizing blue LED light (440–450 nm) as the light source, and achieved good yields ranging from 44% to 99%. The substrate scope was thoroughly explored, demonstrating broad compatibility with various functional groups. Both electron-donating (e.g., methyl, isopropyl, *tert*-butyl) (**78a**–**c**) and electron-withdrawing (e.g., fluorine, chlorine, ester groups) (**78d**–**f**) substituents on bromodifluoroacetamides were well tolerated, yielding high reaction efficiency. Interestingly, the reaction was less sensitive to electronic effects on the aromatic ring, as yields remained high regardless of the substituent type or position. Additionally, substrates with aliphatic groups and naphthyl rings also gave satisfactory results (**78g**–**i**).

**Scheme 38:**
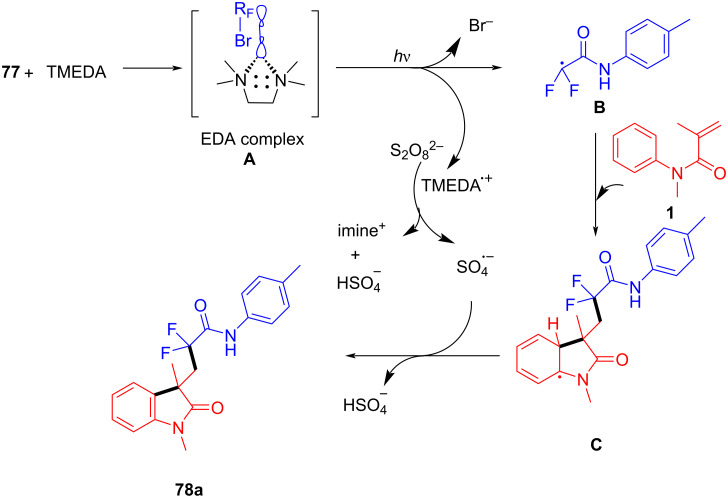
Possible mechanism for the visible-light-promoted radical cyclization of *N*-arylacrylamides with bromodifluoroacetamides via EDA complexes.

The proposed mechanism begins with the formation of an electron donor–acceptor (EDA) complex between TMEDA and bromodifluoroacetamide, leading to the generation of a difluoroacetamide radical **B** through single-electron transfer (SET) (Scheme 38). This radical **B** then adds to *N*-phenylacrylamide, forming a new carbon-centered radical intermediate **C** that undergoes intramolecular cyclization. Ammonium persulfate serves as the oxidant in this system, facilitating dehydrogenation and stabilizing the intermediate. Finally, rearomatization occurs, resulting in the formation of the difluoroamidated oxindole product **78a**.

### *N*-Arylalkenes: alkyl halides C(sp^3^)-centered radicals

**Scheme 39:**
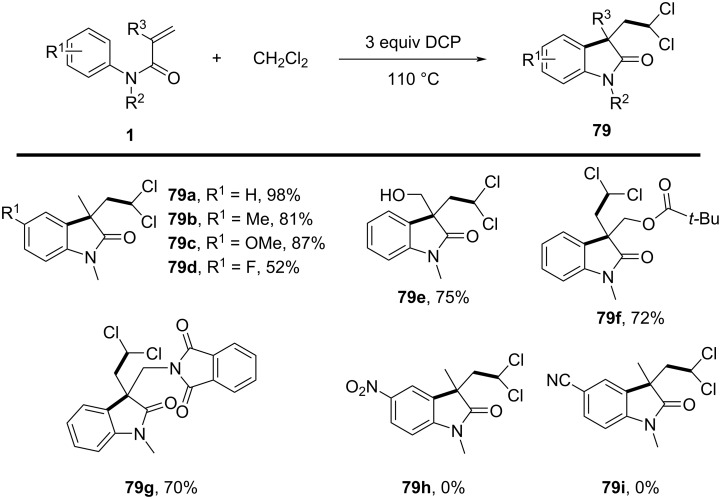
A dicumyl peroxide-initiated radical cascade reaction of *N*-arylacrylamide with DCM.

In 2014, Liu’s group developed a novel method for the selective activation of the C–H bond in dichloromethane (DCM) under metal-free conditions, enabling the efficient synthesis of dichloromethylated oxindoles **79** via a radical cascade process (Scheme 39) [24]. The reaction was initiated by dicumyl peroxide (DCP), which served as the optimal radical initiator. DCM was employed both as a reagent and solvent, and the reaction conditions were optimized to include a temperature of 110 °C in a sealed tube environment. The substrate scope was thoroughly examined, revealing good to excellent yields for a wide range of *N*-arylacrylamides with both electron-donating and electron-withdrawing substituents on the aromatic ring (**79a**–**d**). Functional groups such as hydroxy, ester, and lactam were tolerated, demonstrating the method’s broad applicability (**79e**–**g**). However, strongly electron-withdrawing substituents, such as nitro and cyano groups (**79h,i**), failed to undergo the reaction, likely due to the instability of the intermediate radicals.

**Scheme 40:**
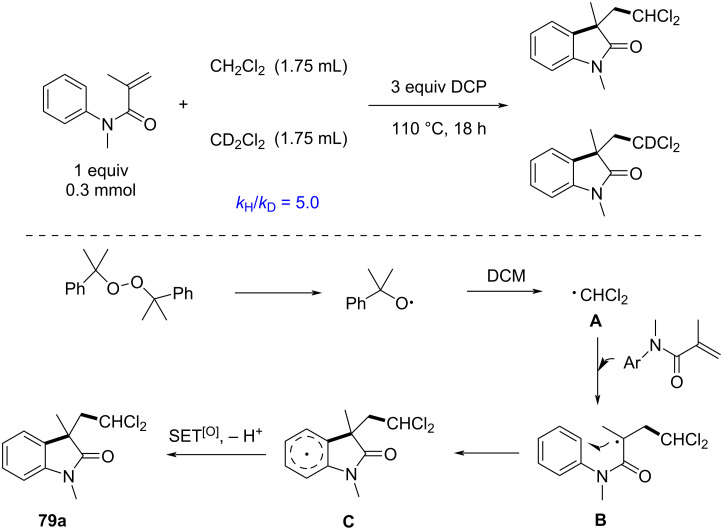
Possible mechanism of radical cyclization of *N*-arylacrylamides with DCM.

Control experiments were conducted to elucidate the reaction mechanism. A kinetic isotope effect (KIE) experiment demonstrated that C–H bond cleavage in DCM was the rate-determining step. Further investigations confirmed that the reaction relies on the generation of dichloromethyl radicals through the homolytic cleavage of DCP, followed by hydrogen abstraction from DCM (Scheme 40). These radicals then undergo addition to *N*-arylacrylamides, followed by cyclization and either hydrogen abstraction or single-electron oxidation to afford the final products.

**Scheme 41:**
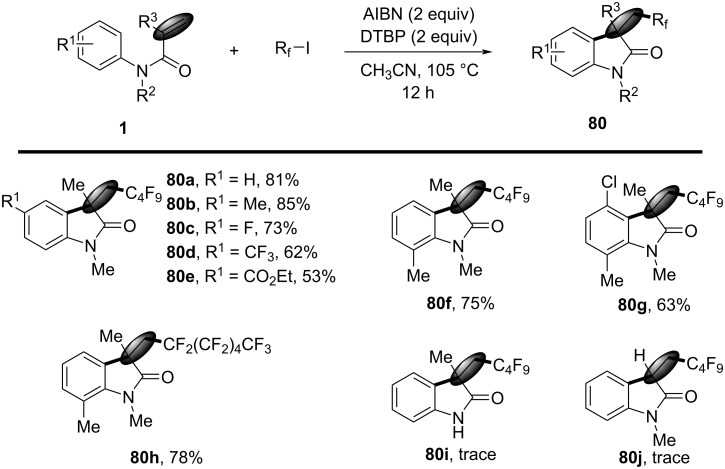
An AIBN-mediated radical cascade reaction of *N*-arylacrylamides with perfluoroalkyl iodides.

In 2015, a metal-free cascade cyclization reaction for synthesizing perfluorinated oxindoles via an AIBN-mediated process was introduced. The reaction utilized *N*-arylacrylamides and commercially available perfluoroalkyl iodides as substrates, achieving high yields of products **80** under mild conditions (Scheme 41) [25]. The reaction was optimized by varying initiators, oxidants, solvents, and temperatures, revealing that AIBN and di-*tert*-butyl peroxide (DTBP) were essential for efficient radical generation and subsequent cyclization. Optimal results were obtained at 105 °C in acetonitrile, with yields reaching up to 81%. The substrate scope was extensively explored, demonstrating compatibility with various *N*-arylacrylamides and perfluoroalkyl iodides. *N*-Arylacrylamides with electron-donating and electron-withdrawing substituents (**80a**–**e**), as well as different substitution patterns on the aromatic ring, gave good to excellent yields (**80f**,**g**). Perfluoroalkyl iodides, ranging from trifluoromethyl to longer perfluorinated chains, also performed well (**80h**). However, substrates with unprotected N–H bonds (**80i**) and monosubstituted olefins (**80j**) exhibited limited reactivity.

**Scheme 42:**
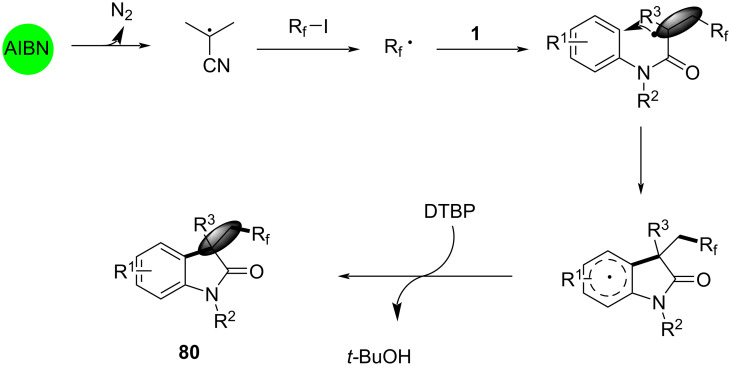
Possible mechanism for the reaction with perfluoroalkyl iodides.

Mechanistic investigations suggested a radical-mediated pathway, as shown in Scheme 42. AIBN initiated the formation of perfluorinated radicals from perfluoroalkyl iodides, which then added to the double bond of *N*-arylacrylamides. The resulting radical intermediate undergoes intramolecular cyclization and hydrogen abstraction to yield the final products **80**. Control experiments confirmed the necessity of both AIBN and DTBP, with higher temperatures favoring the desired cyclization over side reactions.

**Scheme 43:**
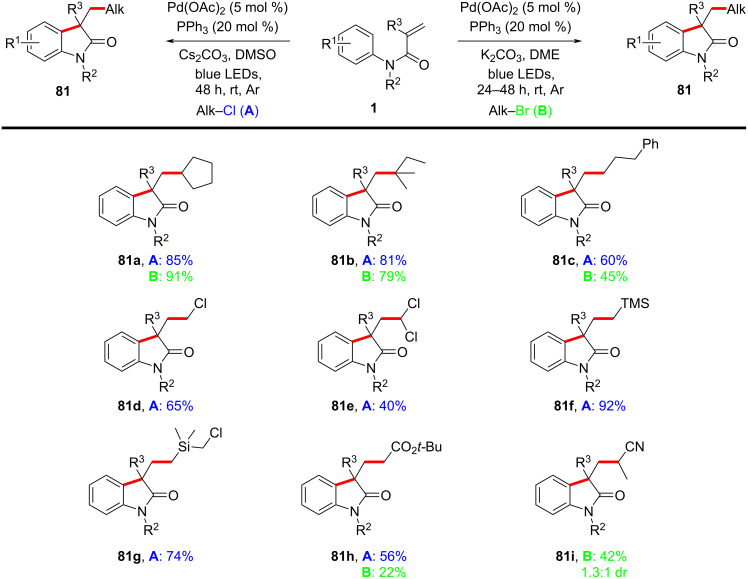
Photoinduced palladium-catalyzed radical annulation of *N*-arylacrylamides with alkyl halides.

**Scheme 44:**
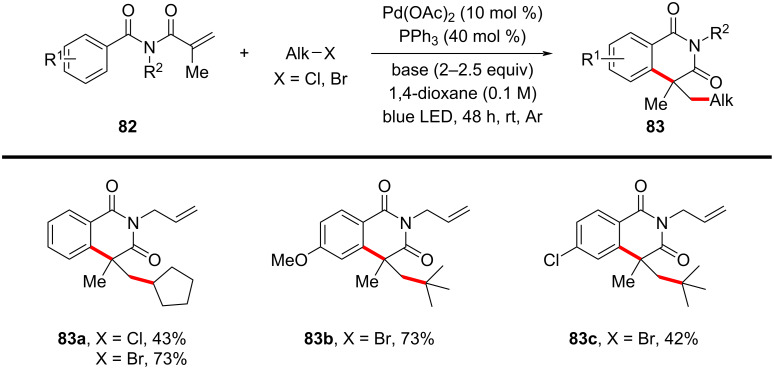
Radical alkylation/cyclization of *N*-Alkyl-*N*-methacryloylbenzamides with alkyl halides.

In 2021, a novel excited-state palladium-catalyzed alkylation/annulation reaction was developed to achieve reaction of unactivated alkyl chlorides, facilitating the synthesis of oxindoles (Scheme 43) and isoquinolinediones (Scheme 44) [26]. In this work, visible light was used to promote a single-electron-transfer (SET) process, overcoming the inherent challenges associated with activating the strong C(sp³)–Cl bond, which typically requires high activation energy (327 kJ/mol) and is less reactive compared to alkyl bromides or iodides. Previous methodologies predominantly relied on alkyl bromides and iodides due to their lower bond dissociation energies. By leveraging the excited-state reactivity of Pd(0) complexes under blue LED irradiation, this method enables the generation of alkyl radicals from alkyl chlorides, thus broadening the scope of substrates available for cross-coupling reactions. The substrate scope was thoroughly investigated, demonstrating broad compatibility with various *N*-methyl-*N*-phenylmethacrylamides and unactivated alkyl chlorides. Primary, secondary, and tertiary alkyl chlorides participated efficiently in the reaction, yielding 3,3-disubstituted oxindoles **81a**–**h** in moderate to excellent yields of 40–92%. Notably, multichlorinated compounds, such as dichloromethane (DCM) and chloroform (CHCl₃), also served as effective alkylating agents, indicating the feasibility of the protocol (**81d**,**e**). The method was further extended to substrates bearing functional groups, such as esters, nitriles, and silanes, all of which were well-tolerated under the optimized conditions (**81f**–**i**). Expanding the substrates to include *N*-allyl-*N*-methacryloylbenzamide derivatives (Scheme 44), both alkyl chlorides and bromides gave the expected six-membered annulated products **83a**–**c** in satisfactory yields.

**Scheme 45:**
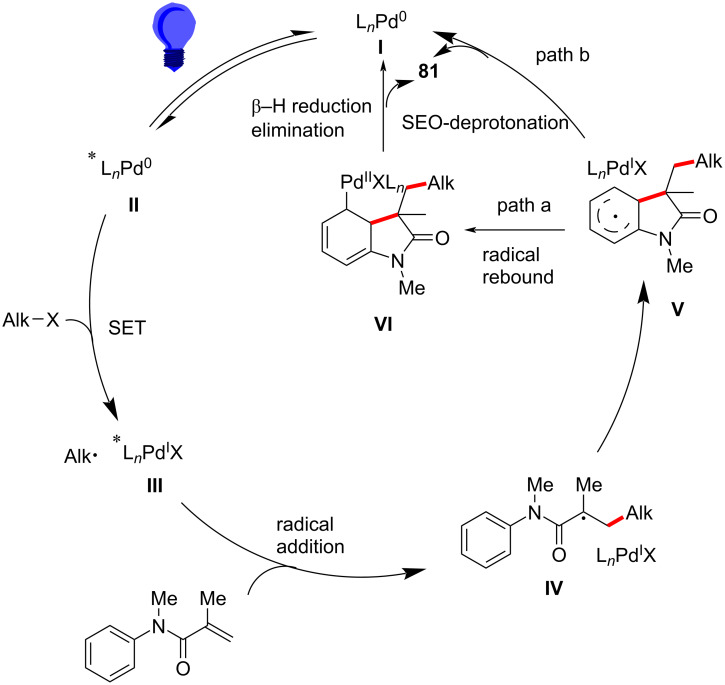
Possible mechanism for the alkylation/cyclization with unactivated alkyl chlorides.

A proposed mechanism, as shown in Scheme 45, involves the photoexcitation of Pd(0) to its excited state **II**, facilitating the single-electron reduction of the alkyl chloride and generating an alkyl–Pd^I^–Cl radical hybrid species **III**. The alkyl radical then adds to the acrylamide double bond, forming a quaternary carbon radical intermediate **IV**. This intermediate undergoes intramolecular radical cyclization onto the aromatic ring, followed by either β-hydride elimination or single-electron oxidation and deprotonation, yielding the final oxindole products **81** and regenerating the Pd(0) catalyst **I**.

**Scheme 46:**
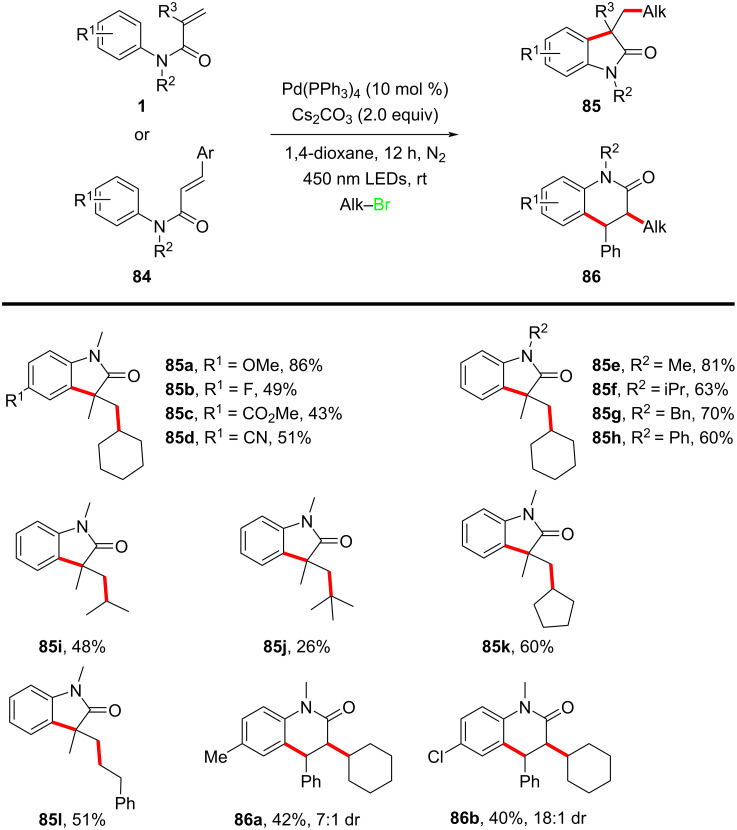
Visible-light-driven palladium-catalyzed radical cascade cyclization of *N*-arylacrylamides with unactivated alkyl bromides.

In the same year, Zhang’s group developed a novel visible-light-induced palladium-catalyzed intermolecular radical cascade cyclization of *N*-arylacrylamides with unactivated alkyl bromides, enabling the efficient construction of functionalized oxindoles and 3,4-dihydroquinolinones under mild conditions, as shown in Scheme 46 [27]. The reaction was catalyzed by Pd(PPh_3_)_4_, without the need for additional photosensitizers, and the alkyl bromides were activated via a single-electron-transfer (SET) process, leading to the formation of hybrid alkyl–Pd radical intermediates. The optimized conditions employed cesium carbonate as the base in 1,4-dioxane, under blue LED irradiation at room temperature, affording the desired products in moderate to good yields.

The substrate scope was extensively explored, demonstrating broad functional group compatibility. Various *N*-arylacrylamides bearing electron-donating and electron-withdrawing substituents were well tolerated, yielding the corresponding oxindoles **85a**–**d** in good yields. Specifically, *N*-substituents such as methyl, isopropyl, benzyl, and phenyl groups had little effect on reaction efficiency (**85e**–**h**). Additionally, unactivated secondary and tertiary alkyl bromides, including both cyclic and acyclic substrates, participated effectively in the reaction (**85i**–**k**). Primary alkyl bromides also reacted, though affording the product **85l** with slightly lower yields. Interestingly, *N*-methyl-*N*-arylcinnamamides proved to be efficient substrates, leading to the smooth formation of the corresponding six-membered-ring products **86a**,**b** with acceptable yields and good diastereomeric ratios (dr).

A key innovation of this work was the direct activation of unactivated alkyl bromides through a visible-light-induced Pd(0)/Pd(I) catalytic cycle, which circumvents the conventional requirement for external photoredox catalysts or high temperatures. This strategy expands the scope of alkyl halide functionalization and highlights the versatility of photoexcited palladium catalysis in radical transformations. Furthermore, the method utilizes the readily available Pd(PPh_3_)_4_ complex as the sole catalyst, making it operationally simple and cost-effective.

**Scheme 47:**
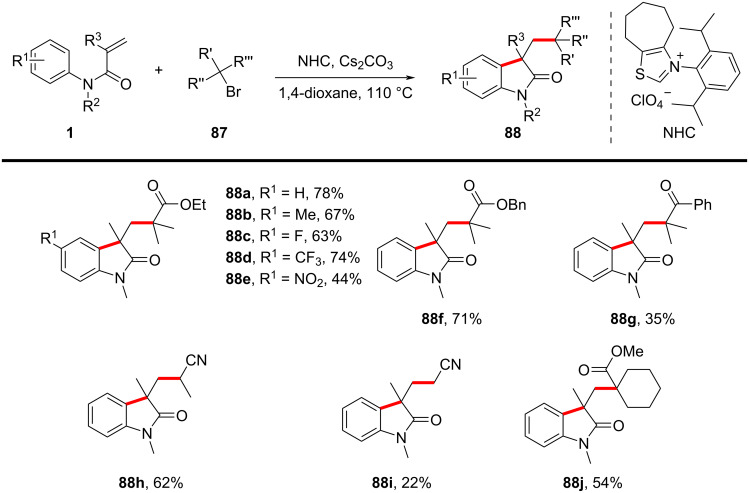
NHC-catalyzed radical cascade cyclization of *N*-arylacrylamides with alkyl bromides.

In 2021, Wang’s group introduced a novel transition-metal-free, aldehyde-free strategy for constructing quaternary carbon centers in oxindoles via an *N*-heterocyclic carbene (NHC)-catalyzed intermolecular Heck-type alkyl radical addition and annulation reaction (Scheme 47) [28]. The reaction proceeds through a redox-neutral mechanism, where the NHC catalyst serves a dual role as both a single-electron reductant and an organocatalyst. The optimized conditions involve using an NHC precursor, cesium carbonate as the base, and 1,4-dioxane as the solvent at 110 °C, yielding structurally diverse oxindoles **88** with satisfactory efficiency. The substrate scope was systematically investigated, revealing broad functional group tolerance. A series of *N*-arylacrylamides bearing electron-donating and electron-withdrawing substituents on the aryl ring were well tolerated, affording the desired oxindoles **88a**–**e** in moderate to high yields. Additionally, various radical precursors, including α-bromo esters, α-bromo ketones, and α-bromo nitriles, effectively participated in the reaction (**88f**–**i**). Even a more sterically hindered substrate also underwent successful cyclization affording **88j**, thus further underscoring the robustness of this transformation. However, attempts using benzyl bromide, allyl bromide, and secondary alkyl bromides failed to yield the desired products, suggesting limitations in radical generation efficiency for certain substrates.

**Scheme 48:**
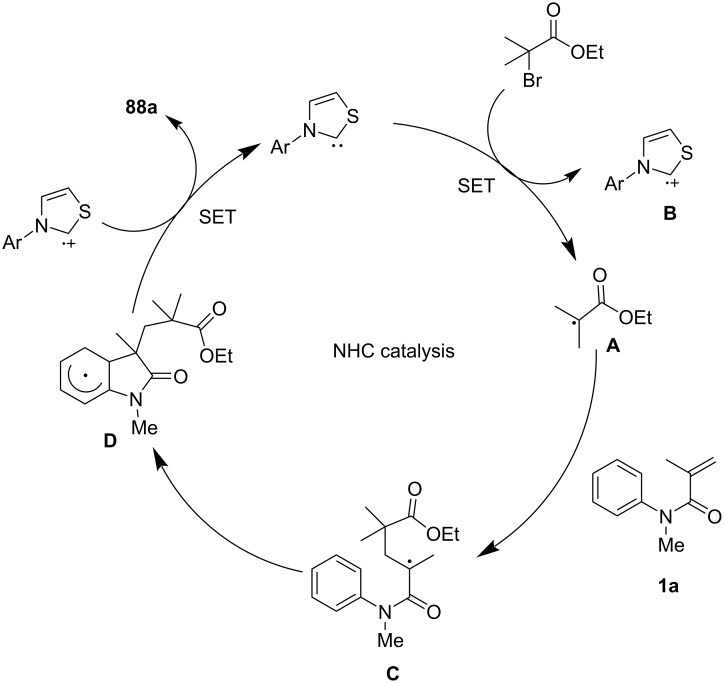
Possible mechanism of NHC-catalyzed radical cascade cyclization.

A plausible mechanism, as shown in Scheme 48, involves the NHC catalyst donating a single electron to the α-bromo substrate, generating an NHC radical cation **B** and an α-carbon radical **A**. The α-carbon radical **A** then undergoes intermolecular radical addition to the acrylamide, forming a new carbon-centered radical intermediate **C**, which subsequently undergoes intramolecular cyclization to yield the oxindole product **88a** via homolytic aromatic substitution (HAS) and deprotonation.

**Scheme 49:**
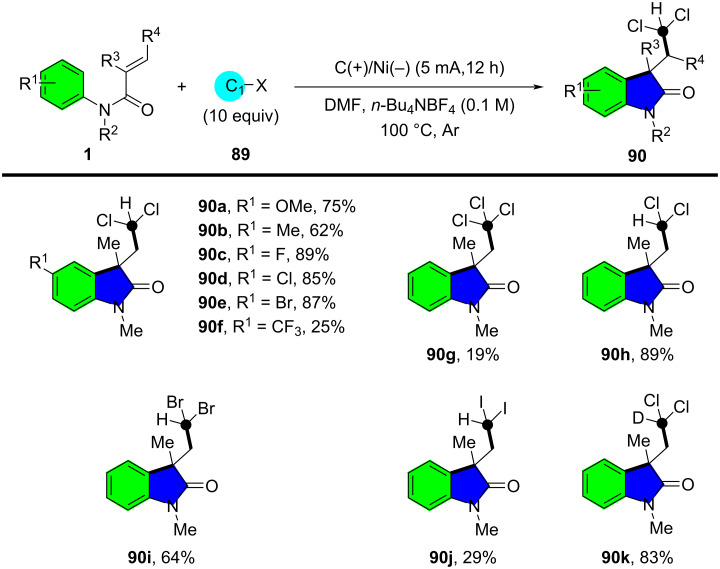
Electrochemically mediated radical cyclization reaction of *N*-arylacrylamides with freon-type methanes.

In 2022, Zhang’s group introduced an innovative electrochemical approach for the collective synthesis of labeled pyrroloindoline alkaloids, utilizing Freon-type methanes as functional one-carbon (C_1_) synthons (Scheme 49) [29]. This methodology employed an electroreductive C–X-bond cleavage to generate halomethyl radicals, which were then captured by acrylamides, leading to the formation of various halogenated oxindoles **90** via radical cyclization. The optimized reaction conditions involved an undivided electrochemical cell with a carbon felt anode and a foam nickel cathode, operated at a constant current of 5 mA in *N*,*N*-dimethylformamide (DMF) containing *n*-Bu_4_NBF_4_ as the electrolyte. The reaction proceeded efficiently at 100 °C under an inert atmosphere, yielding halogenated oxindoles in good to excellent yields. The substrate scope was systematically explored, revealing broad functional group tolerance. Various *N*-arylacrylamides bearing electron-donating and electron-withdrawing groups on the aryl ring reacted smoothly, affording the corresponding halogenated oxindoles **90a**–**e** in moderate to high yields. Substituents such as alkyl, methoxy, halogens (Cl, F, Br), and esters were well tolerated. However, substrates with strongly electron-withdrawing CF_3_ groups exhibited significantly lower reactivity (**90f**), suggesting that electron density on the aromatic ring influences the reaction outcome. A variety of halomethanes were also evaluated, with bond-cleavage reactivity following the trend C–I > C–Br > C–Cl, consistent with bond dissociation energies (**90g**–**j**). Notably, deuterated chloroform was successfully employed to obtain deuterated oxindole **90k** in high yield, demonstrating the method's potential for isotope labeling applications.

**Scheme 50:**
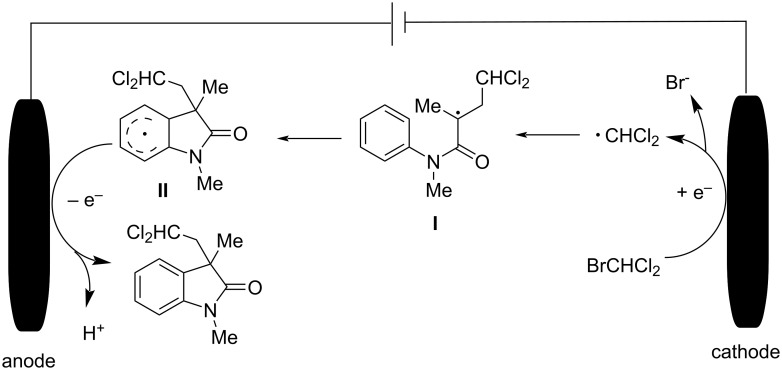
Proposed mechanistic pathway of electrochemically induced radical cyclization reaction.

A plausible mechanism, as outlined in Scheme 50, involves single-electron reduction of the halomethane at the cathode, generating a halomethyl radical and a corresponding halide ion. The halomethyl radical is then captured by the acrylamide, leading to intramolecular radical cyclization. Final oxidation and deprotonation steps afford the halogenated oxindole product.

**Scheme 51:**
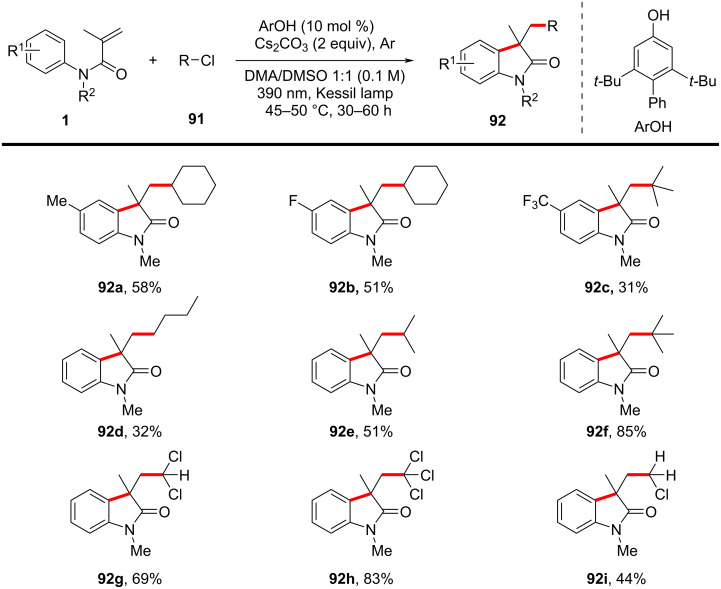
Redox-neutral photoinduced radical cascade cylization of *N-*arylacrylamides with unactivated alkyl chlorides and aryl chlorides.

In 2023, Kuniyil and Yatham’s group reported a redox-neutral, metal-free radical cascade cyclization strategy for the functionalization of *N*-arylacrylamides using unactivated alkyl and aryl chlorides, enabling the efficient construction of 3,3-disubstituted oxindoles **92** under mild conditions (Scheme 51) [30]. The transformation was mediated by a phenolate anion photocatalyst and proceeded via a single-electron transfer (SET) mechanism. A comprehensive investigation of the substrate scope demonstrated its wide applicability. Various *N*-arylacrylamides bearing electron-donating and electron-withdrawing substituents reacted efficiently, affording the corresponding oxindoles **92a**–**c** in moderate to good yields of 30–60%. Functionalized primary, secondary, and tertiary alkyl chlorides, including cyclic and acyclic substrates, were successfully employed, with tertiary alkyl chlorides exhibiting the highest reactivity (up to 90% yield). Furthermore, simple chlorinated solvents such as CCl_4_, CHCl_3_, CDCl_3_, and CH_2_Cl_2_ were utilized as one-carbon (C_1_) synthons, yielding moderate to good results for products **92g**–**i** (44–83%). The method was also extended to aryl chlorides, with electron-deficient arenes performing well, whereas electron-rich aryl chlorides exhibited lower reactivity.

**Scheme 52:**
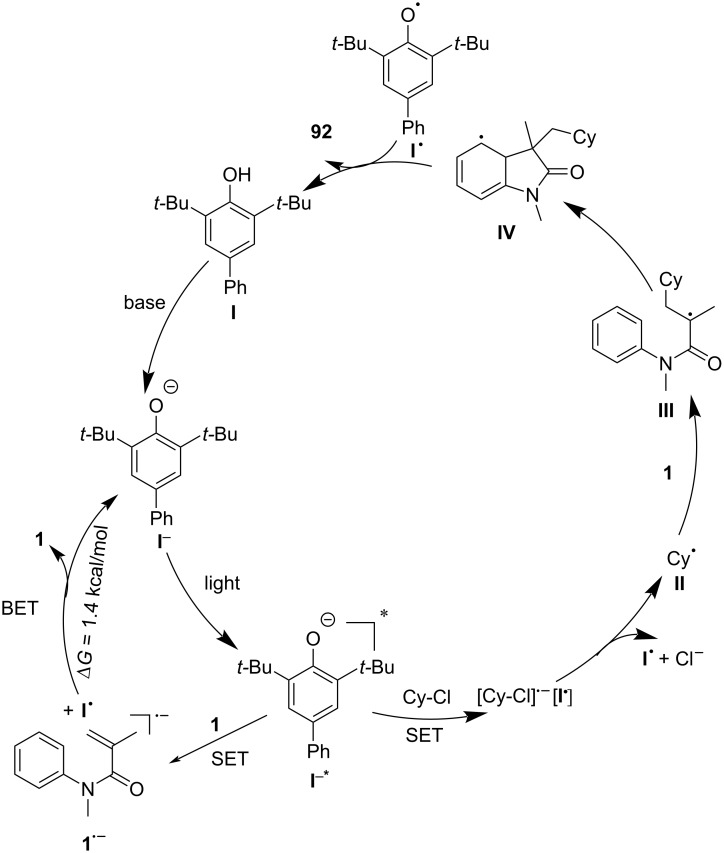
Proposed mechanistic hypothesis of redox-neutral radical cascade cyclization.

A plausible reaction mechanism, as outlined in Scheme 52, involves the photoexcited phenolate anion I^−*^ (generated through excitation of phenolate **I**^−^) undergoing a SET with the alkyl chloride, generating an alkyl radical **II** and a phenoxide radical **I^·^**. The alkyl radical **II** then adds to the *N*-arylacrylamide substrate **1**, forming a new carbon-centered radical **III**, which undergoes intramolecular cyclization to generate an aryl radical intermediate **IV**. The final product is formed through either hydrogen atom transfer (HAT) or oxidative deprotonation, regenerating the phenolate photocatalyst. Density functional theory (DFT) calculations further supported the reaction mechanism, revealing that the back-electron transfer process is more favorable for *N*-arylacrylamides than for alkyl chlorides, thus explaining the selective radical generation and subsequent cyclization.

**Scheme 53:**
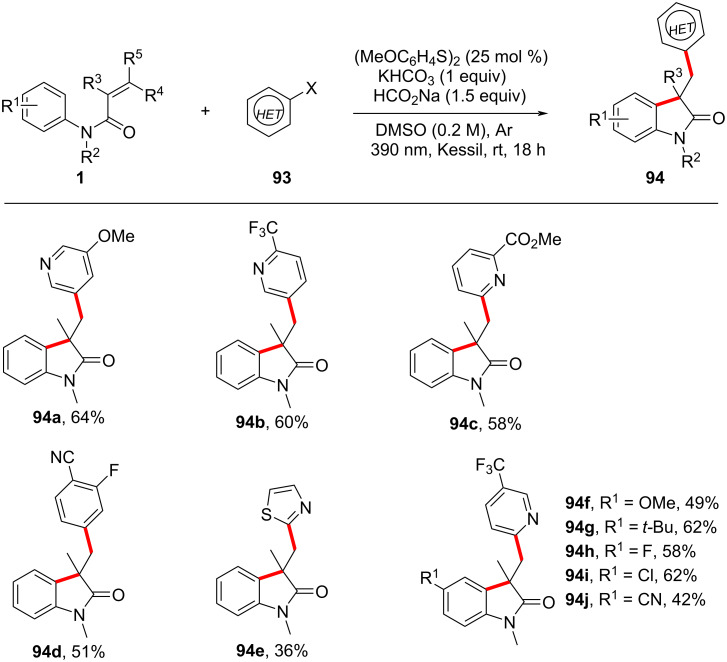
Thiol-mediated photochemical radical cascade cylization of *N-*arylacrylamides with aryl halides.

The development of a visible-light-induced, transition-metal-free heteroarylation strategy for constructing 3,3'-disubstituted oxindoles has provided a sustainable alternative to traditional metal-catalyzed cross-coupling reactions (Scheme 53) [31]. By employing a thiol-mediated photochemical approach, this transformation eliminates the need for Pd-, Ni-, or Cu-based catalysts, facilitating the single-electron transfer (SET) activation of aryl halides under 390 nm LED irradiation. A broad substrate scope was observed, with various electron-donating (OMe, Me) and electron-withdrawing (CF₃, CN, CO₂Me) substituents on aryl halides or *N*-arylacrylamides exhibiting high reactivity. Furthermore, heteroaryl halides were well tolerated.

**Scheme 54:**
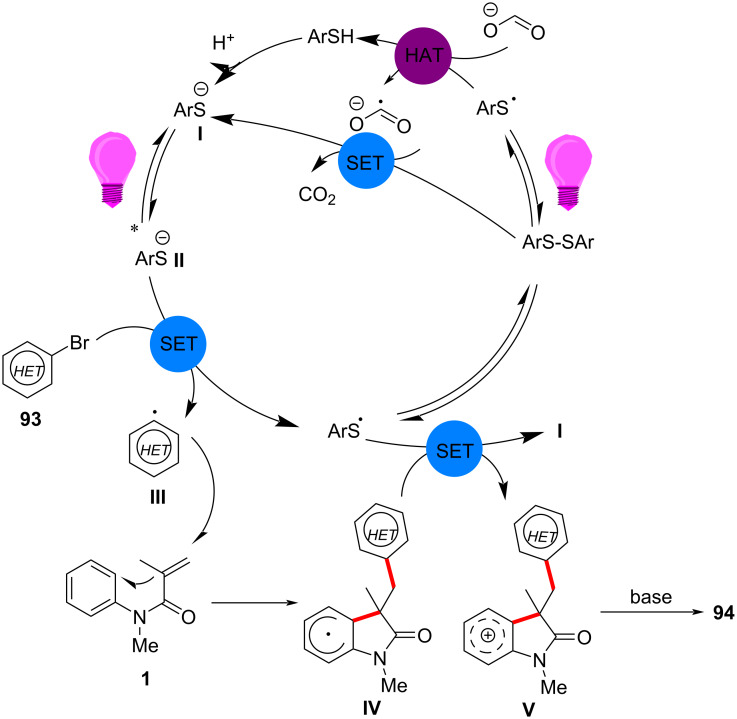
Proposed possible mechanism of thiol-mediated photochemical radical cascade cyclization.

Mechanistic investigations provided substantial evidence for a radical-mediated pathway. Control experiments with radical scavengers, such as TEMPO, completely suppressed product formation, while fluorescence quenching studies confirmed that thiol activation was crucial for initiating the SET process. The transformation was initiated by the photoexcitation of the thiol catalyst, which generated aryl thiolate anions **I** and thiyl radicals (Scheme 54). These radicals then reduce aryl halides via a SET, producing reactive aryl radicals **III** that add to the acrylamide double bond, forming a new carbon-centered radical **IV**. The reaction concludes with intramolecular radical cyclization, followed by rearomatization to afford the final oxindole products **94**.

**Scheme 55:**
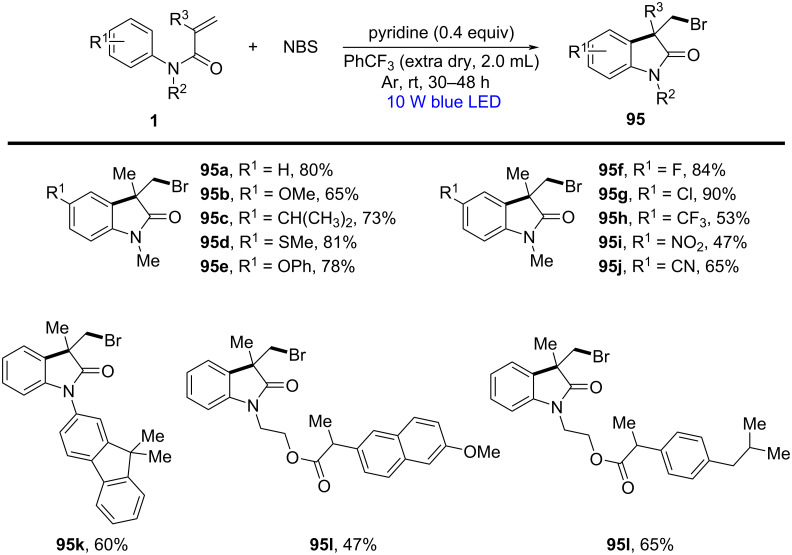
Visible-light-induced radical cascade bromocyclization of *N-*arylacrylamides with NBS.

In 2024, Zhang and co-workers developed a highly chemoselective radical bromocyclization strategy that enabled the efficient synthesis of 3-bromomethyloxindoles **95** under mild, metal-free conditions (Scheme 55) [32]. Traditional bromination methods often suffer from overbromination of the benzene ring, leading to undesired regioisomeric products. This study successfully mitigated these challenges by employing *N*-bromosuccinimide (NBS) and pyridine in an anhydrous medium under blue LED irradiation (10 W, room temperature, 30 h), where pyridine was found to suppress in situ Br_2_ formation, thereby preventing unwanted aromatic bromination. The substrate scope was extensively explored, demonstrating excellent selectivity across various *N*-arylacrylamides. Both electron-donating (-OMe, -PhO, -MeS, -alkyl) and electron-withdrawing (-F, -Cl, -CF_3_, -NO_2_, -CN) substituents were compatible, highlighting the versatility of this protocol.

**Scheme 56:**
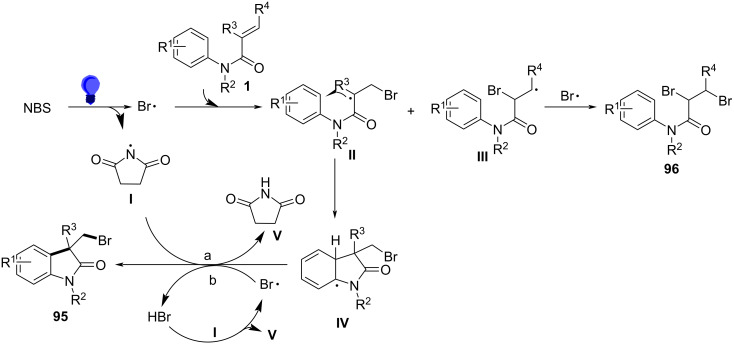
Possible mechanism of visible-light-induced radical cascade cyclization.

Mechanistic investigations provided substantial evidence for a radical-mediated bromination process. Radical trapping experiments using TEMPO completely inhibited product formation, confirming the involvement of radical intermediates. Kinetic isotope effect (KIE) studies further indicated that the initial radical formation was not rate-limiting, while Hammett analysis revealed that electron-rich acrylamides enhanced radical addition rates. The reaction is initiated by the photoexcitation of NBS under blue LED irradiation, generating bromine radicals via homolytic cleavage (Scheme 56). These radicals selectively add to the terminal carbon of the acrylamide double bond, producing a carbon-centered radical **II** that undergoes intramolecular radical cyclization onto the aryl ring. The final step involved re-aromatization via hydrogen atom transfer (HAT), yielding the desired bromomethyloxindole product with high chemoselectivity.

### *N*-Arylalkenes: alkyl carboxylic acids C(sp^3^)-centered radicals

**Scheme 57:**
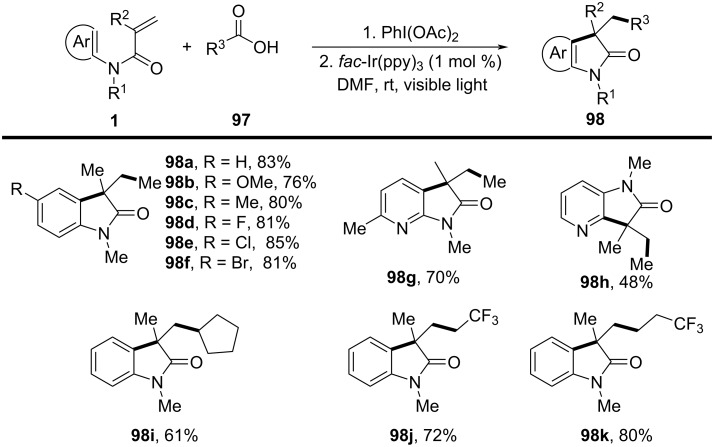
Decarboxylation/radical C–H functionalization by visible-light photoredox catalysis.

Apart from halide-based radical precursors, alkyl carboxylic acids also serve as efficient and accessible sources of alkyl radicals, especially in visible-light-induced decarboxylative cyclizations. In 2013, a visible-light-mediated decarboxylative tandem reaction was developed for the efficient synthesis of 3,3-disubstituted oxindoles under mild conditions (Scheme 57) [33]. By employing phenyliodine(III) dicarboxylate (DIB) as both a decarboxylation reagent and a radical initiator, and fac-Ir(ppy)_3_ as a photocatalyst, this method enabled radical-mediated C–H functionalization and multiple C–C-bond formations at room temperature, overcoming the need for high-energy UV irradiation or elevated temperatures. The reaction proceeded under 35 W fluorescent light irradiation in DMF, where DIB facilitated the selective decarboxylation of aliphatic carboxylic acids to generate alkyl radicals. The reaction demonstrated a broad substrate scope, tolerating a variety of *N*-arylacrylamides with electron-donating (-Me, -OMe) and electron-withdrawing (-Cl, -Br, -F) substituents, affording 3,3-disubstituted oxindoles **98a–f** in yields of 76–85%. Notably, the method also exhibited excellent chemoselectivity, with selective C–H functionalization observed in the presence of multiple reactive sites (**98h**). Additionally, the incorporation of trifluoropropanoic and trifluorobutanoic acids demonstrated the method’s potential for introducing bioactive trifluoromethyl functionalities into oxindole scaffolds.

**Scheme 58:**
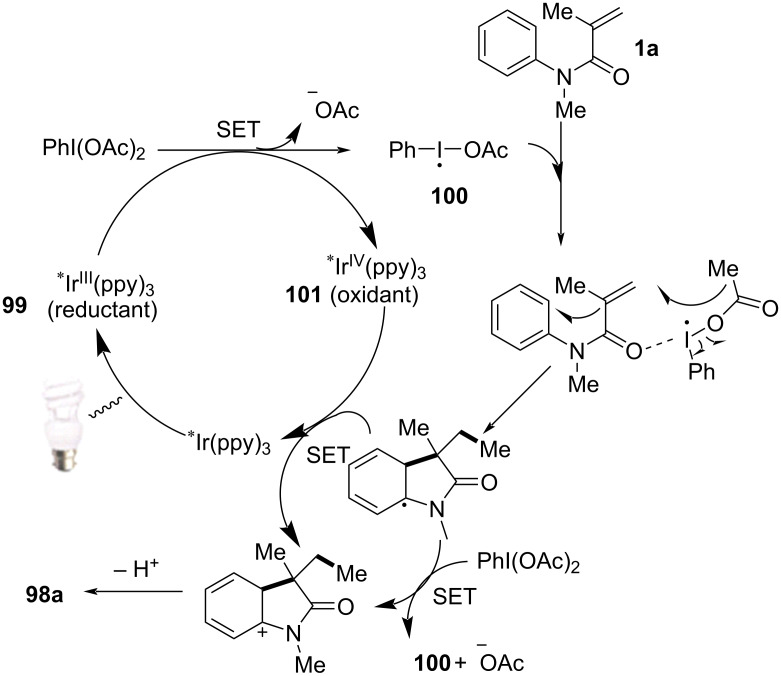
Plausible mechanism of visible-light photoredox-catalyzed radical cascade cyclization.

Mechanistic studies confirmed a radical-mediated process. The reaction did not proceed in the absence of light or photocatalyst, and alternative photocatalysts such as Ru(bpy)_3_Cl_2_ and Ir(ppy)_2_(dtbbpy)BF_4_ resulted in lower yields, highlighting the unique efficiency of fac-Ir(ppy)_3_. Radical trapping experiments with TEMPO completely suppressed the reaction, further supporting the radical pathway. The mechanism involves photoexcitation of fac-Ir(ppy)_3_, which undergoes single-electron transfer (SET) with DIB, generating an iodine radical **100** that induces decarboxylation to form alkyl radicals. These radicals then add to the acrylamide double bond, followed by intramolecular radical cyclization and oxidation, leading to the final oxindole product **98a** (Scheme 58).

**Scheme 59:**
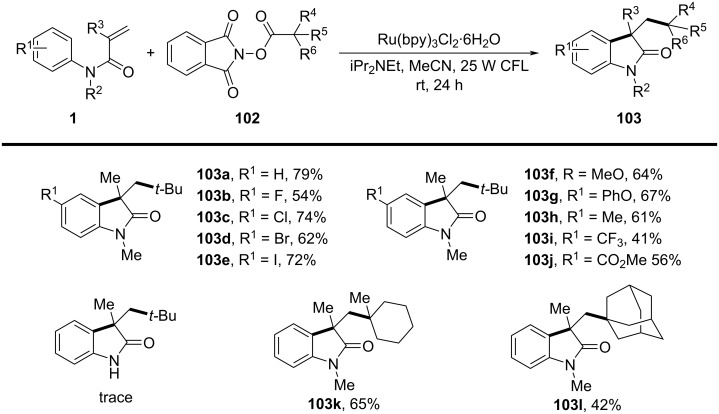
Visible-light-promoted tandem radical cyclization of *N*-arylacrylamides with *N*-(acyloxy)phthalimides.

In 2015, a visible-light-induced tandem radical cyclization was developed for the efficient synthesis of 3,3-dialkyl-substituted oxindoles from *N*-arylacrylamides and *N*-(acyloxy)phthalimides, which serve as tertiary alkyl radical precursors (Scheme 59) [34]. Using Ru(bpy)_3_Cl_2_·6H_2_O as a photocatalyst and iPr_2_NEt as a base, this reaction proceeded under mild conditions with a 25 W compact fluorescent bulb at room temperature, avoiding the need for peroxides or strong oxidants typically required for radical generation. A comprehensive evaluation of the substrate scope revealed that various *N*-arylacrylamides bearing halogen (-F, -Cl, -Br, -I), electron-donating (-MeO, -PhO, -Me), and electron-withdrawing (-CF_3_, -CO_2_Me) groups were well tolerated, affording the corresponding 3,3-dialkyl-substituted oxindoles **98a–f** in yields of 54–79%. However, when *N*-unsubstituted acrylamides were tested, no cyclization occurred, highlighting the essential role of the *N*-substituent in stabilizing radical intermediates.

**Scheme 60:**
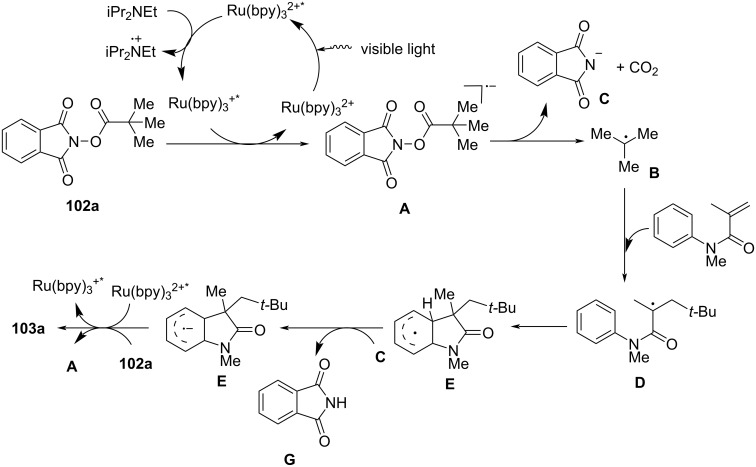
Plausible mechanism for the tandem radical cyclization reaction.

Mechanistic investigations confirmed the involvement of a radical pathway (Scheme 60). The reaction was completely inhibited in the absence of light or photocatalyst, demonstrating the necessity of photoredox activation. Additionally, replacing iPr_2_NEt with inorganic bases such as K_2_CO_3_ resulted in no product formation, indicating that the organic base plays a crucial role in facilitating SET processes. Radical trapping experiments using TEMPO completely suppressed the reaction, further supporting the radical mechanism. The proposed pathway begins with the photoexcitation of Ru(bpy)_3_^2+^, which undergoes a single-electron transfer (SET) with iPr_2_NEt, generating Ru(bpy)_3_^+^ and a strongly reducing species. This intermediate then reduces *N*-(acyloxy)phthalimide, forming a radical anion **A** that undergoes decarboxylation to generate a tertiary alkyl radical **B**. The resulting radical subsequently adds to the electron-deficient acrylamide, followed by intramolecular cyclization and oxidation, ultimately yielding the oxindole product **103a**.

**Scheme 61:**
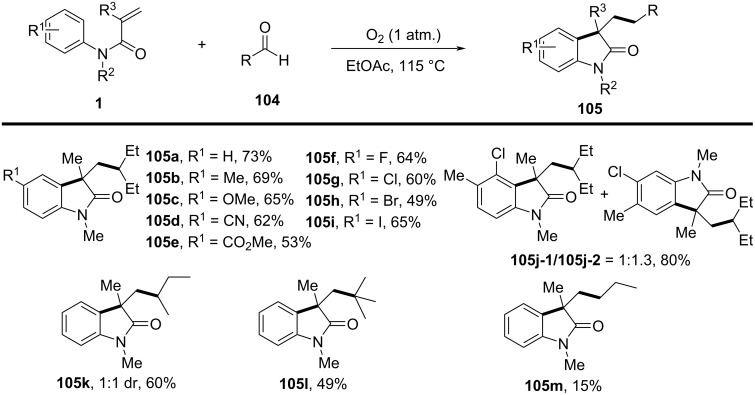
Visible-light-induced aerobic radical cascade alkylation/cyclization of *N*-arylacrylamides with aldehydes.

In 2016, a visible-light-induced aerobic radical cascade alkylation/cyclization was developed for the efficient synthesis of 3,3-disubstituted oxindoles using aldehydes as alkyl radical precursors (Scheme 61) [35]. This transition-metal-free transformation operated under ambient oxygen (1 atm) in ethyl acetate, leveraging an oxygen-mediated auto-oxidation/decarbonylation process to generate alkyl radicals directly from aldehydes. The reaction demonstrated broad substrate tolerance, affording the oxindoles in moderate to excellent yields (73–90%) across a range of α-branched aldehydes and *N-*arylacrylamides **105a**–**i** bearing electron-donating (-Me, -OMe) and electron-withdrawing (-CN, -CO_2_Me) groups. While sterically hindered *tert*-butyl-derived oxindoles were efficiently synthesized, linear aldehydes exhibited lower reactivity, likely due to less favorable radical formation. Halogenated arenes remained intact, allowing for further derivatization, whereas *meta*-substituted arenes led to regioisomeric mixtures.

**Scheme 62:**
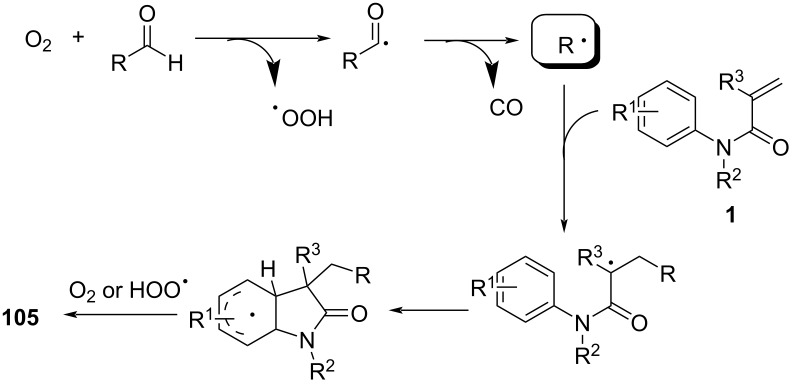
Plausible mechanism for the aerobic radical alkylarylation of electron-deficient amides.

Mechanistic studies confirmed a radical-mediated process, as the reaction was completely suppressed by TEMPO and proceeded only under oxygen. The reaction pathway involves an auto-oxidation of aldehydes to generate acyl radicals, which undergo decarbonylation to form alkyl radicals (Scheme 62). These radicals then add to the acrylamides, followed by intramolecular cyclization and oxidative rearomatization, ultimately affording the final oxindole product.

**Scheme 63:**
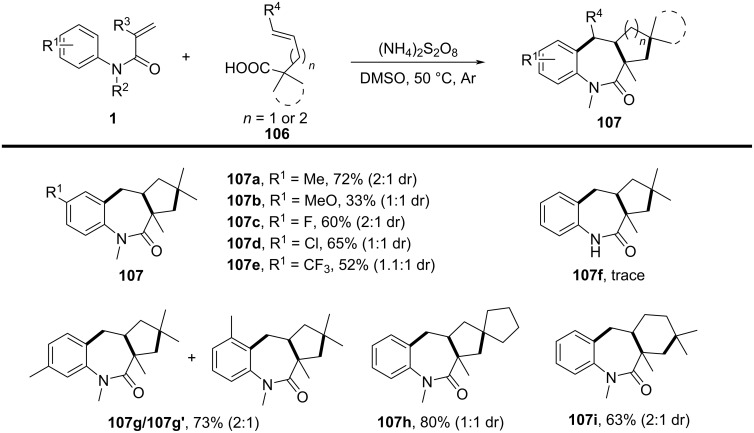
Oxidative decarbonylative [3 + 2]/[5 + 2] annulation of *N*-arylacrylamide with vinyl acids.

In 2020, a metal-free decarboxylative cyclization reaction was developed for the synthesis of benzo[*b*]azepin-2-ones from *N*-arylacrylamides and vinyl acids under oxidative conditions (Scheme 63) [36]. This transformation enabled the formation of three new C–C bonds in a single step via a (3 + 2)/(5 + 2) cyclization, using (NH_4_)_2_S_2_O_8_ as an oxidant in DMSO at 50 °C. The reaction was carried out under an argon atmosphere, providing a transition-metal-free approach to seven-membered *N*-heterocycles with excellent functional group tolerance. Unlike prior strategies relying on transition-metal catalysis (Ag, Cu) or photoredox conditions, this method achieved efficient annulation without the need for external photocatalysts or expensive ligands. The reaction demonstrated a broad substrate scope, tolerating various *N*-arylacrylamides substituted with electron-donating (-Me, -OMe) and electron-withdrawing (-CF_3_) groups, giving the product **107a–f** with yields ranging from 33% to 72%. Additionally, halogenated acrylamides (-F, -Cl, -Br, -I) were compatible, while sterically hindered *ortho*-substituted substrates reacted with moderate efficiency. However, attempts to use *N*–H acrylamides resulted in no product formation, highlighting the importance of *N*-substitution in stabilizing radical intermediates.

**Scheme 64:**
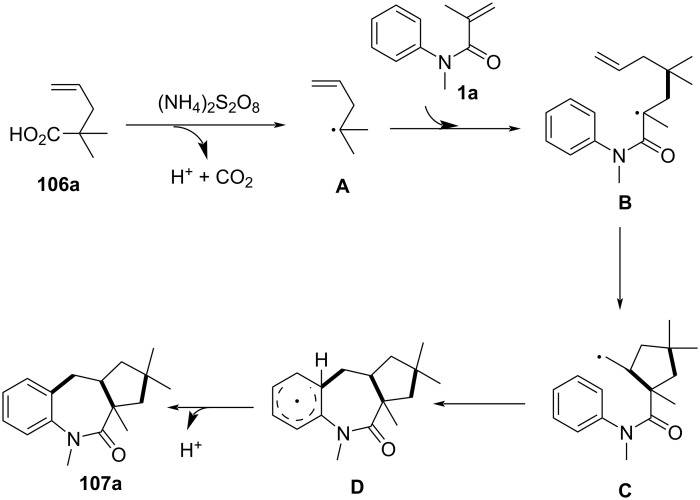
Plausible mechanism for the decarboxylative (3 + 2)/(5 + 2) annulation between *N*-arylacrylamides and vinyl acids.

Mechanistic investigations confirmed the radical nature of this transformation. The reaction was completely suppressed upon addition of radical scavengers such as TEMPO, BHT, and hydroquinone, indicating the involvement of alkyl radical intermediates. Furthermore, reactions performed under an O_2_ atmosphere led to significantly lower yields, suggesting that excess oxygen inhibited radical propagation. A stepwise radical pathway was proposed (Scheme 64), wherein oxidative decarboxylation of the vinyl acid generates an alkyl radical **A**, which adds to the acrylamide C=C bond, forming a carbon-centered radical intermediate **B**. Subsequent intramolecular cyclization onto the aryl ring, followed by oxidation and rearomatization, ultimately affords the desired benzo[*b*]azepin-2-one product **107a**.

**Scheme 65:**
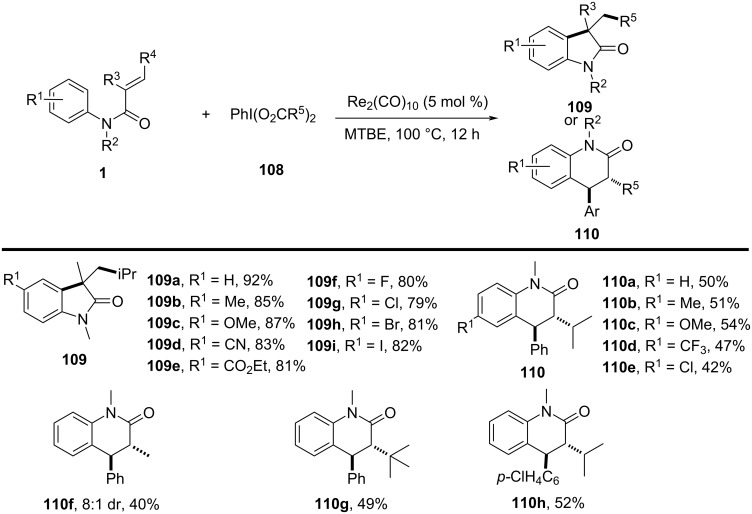
Rhenium-catalyzed alkylarylation of alkenes with PhI(O_2_CR)_2_.

In the same year, Wang’s group presented a rhenium-catalyzed alkylarylation of alkenes with PhI(O_2_CR)_2_ via decarboxylation, providing a novel and efficient method for synthesizing indolinones and dihydroquinolinones (Scheme 65) [37]. This method utilized arylperfluorobenzoates as both electrophilic alkylating agents and decarboxylating reagents, generating highly reactive alkyl radicals upon decarboxylation. The reaction proceeded under mild, metal-free conditions, employing a simple rhenium catalyst, making it a green and sustainable synthetic route to functionalized heterocycles. The substrate scope was broad, accommodating various alkenes, including electron-rich and electron-deficient types, with good tolerance for functional groups such as halogens, methyl, methoxy, cyano, trifluoromethyl, and ester groups (**109a**–**i** and **110a**–**h**).

**Scheme 66:**
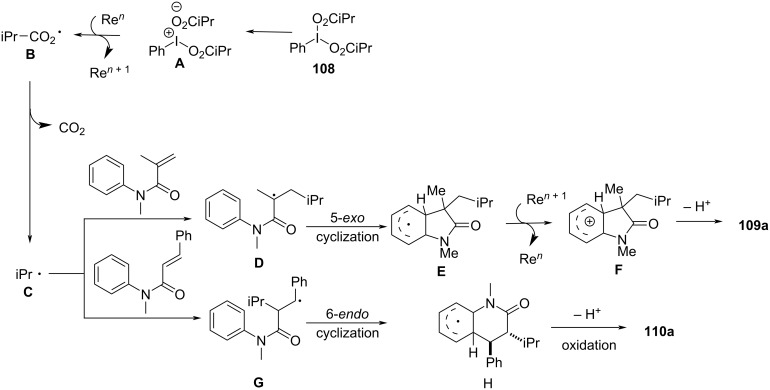
Plausible mechanism for the rhenium-catalyzed decarboxylative annulation of *N*-arylacrylamides with PhI(O_2_CR)_2_.

Control experiments confirmed the importance of both the rhenium catalyst and the decarboxylative reagent, as well as the involvement of radical intermediates. This was further validated by radical scavenger studies and isotope labeling experiments. Mechanistically, the reaction begins with activation of PhI(O_2_CR)_2_ by the rhenium catalyst, followed by decarboxylation to generate an isopropyl radical **C** that adds to the alkene, forming a carbon-centered radical intermediate **D** (**G**) (Scheme 66). This intermediate undergoes intramolecular cyclization onto the aromatic ring, ultimately producing the desired oxindole product **109a** (**110a**) via protonation and rearomatization.

**Scheme 67:**
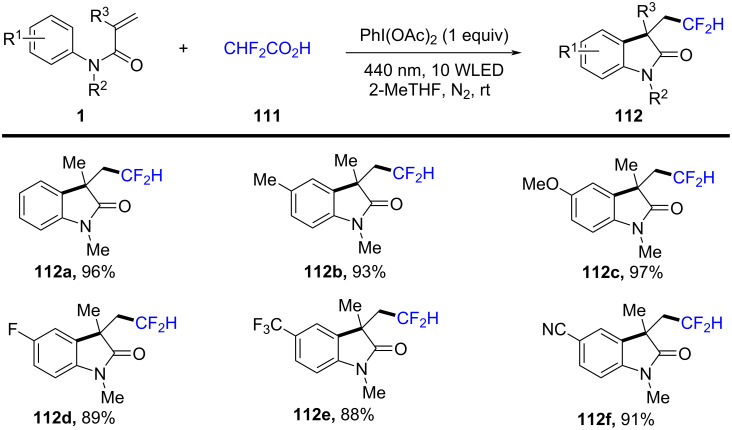
Visible-light-induced one-pot tandem reaction of *N*-arylacrylamides.

In 2021, He and co-workers introduced a visible-light-initiated tandem synthesis for difluoromethylated oxindoles, conducted under additive-, metal catalyst-, and external photosensitizer-free conditions in 2-methyltetrahydrofuran (2-MeTHF) (Scheme 67) [38]. This reaction represented a significant innovation by using visible light as the sole activation source, eliminating the need for metal catalysts or toxic reagents, thereby offering a greener and more sustainable alternative for functionalizing oxindoles.

The reaction exhibited a broad substrate scope, tolerating a wide range of electron-rich and electron-deficient substituents on the aromatic rings, with moderate to good yields for various oxindole derivatives and difluoromethylation reagents (**112a–f**). The use of 2-MeTHF as a solvent was crucial for enhancing reactivity and solubility, while maintaining environmental compatibility.

**Scheme 68:**
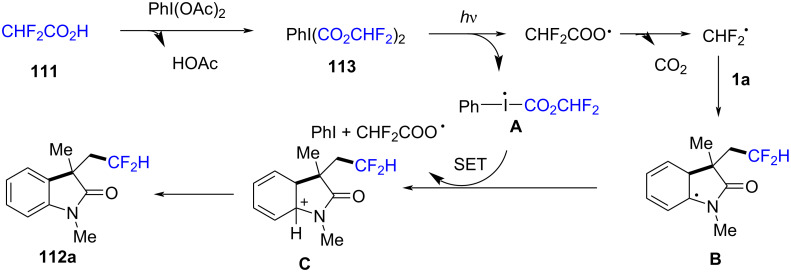
Plausible mechanism for the visible-light-initiated tandem synthesis of difluoromethylated oxindoles.

Control experiments provided insight into the radical nature of the reaction, with TEMPO completely suppressing product formation. Moreover, the reaction did not proceed under argon or in the absence of light, confirming the essential role of visible light in initiating the process. The mechanistic pathway begins with the photoexcitation of the difluoromethylation reagent **113** under visible light, generating a reactive radical intermediate (Scheme 68). This radical adds to the oxindole C=C bond, forming a carbon-centered radical intermediate. The intermediate then undergoes intramolecular cyclization, followed by radical recombination and rearomatization, ultimately leading to the difluoromethylated oxindole product **112a**.

**Scheme 69:**
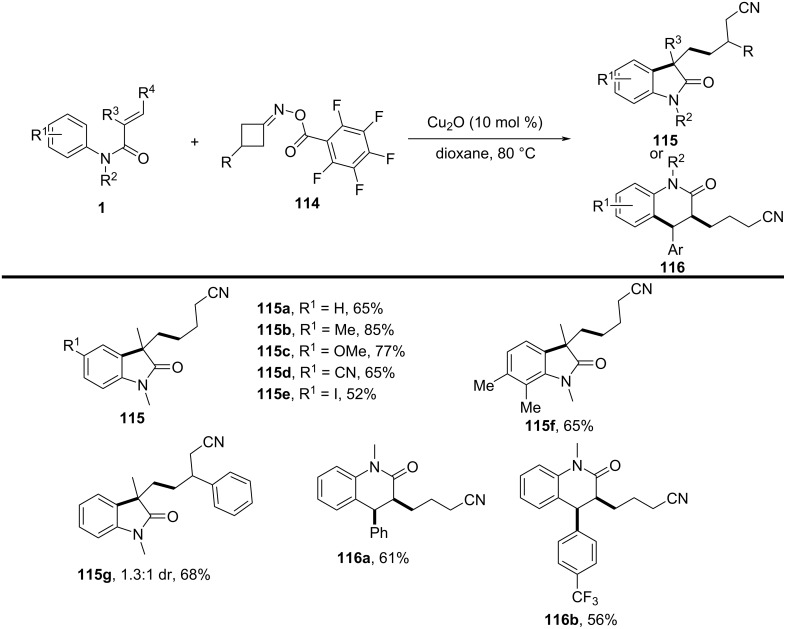
Copper-catalyzed redox-neutral cyanoalkylarylation of activated alkenes with cyclobutanone oxime esters.

In 2017, Guo’s group introduced a copper-catalyzed, redox-neutral cyanoalkylarylation of activated alkenes with cyclobutanone oxime esters to form cyanoalkylated aryl compounds (Scheme 69) [39]. This reaction was notable for its mild conditions, excellent functional group tolerance, and efficient formation of complex molecules in a single step, without requiring additional oxidants or reducing agents.

The substrate scope of the reaction was broad, as various activated alkenes and cyclobutanone oxime esters with different substituents were efficiently cyanoalkylated under the reaction conditions. The method was compatible with electron-rich and electron-deficient alkenes, demonstrating high tolerance to a variety of functional groups, such as halogens, methoxy groups, and cyano groups. Both, monosubstituted and disubstituted alkenes reacted well, with the reaction proceeding smoothly at room temperature in most cases. The cyclobutanone oxime esters could also be derived from a variety of aromatic and aliphatic ketones, showing good flexibility in the choice of substrates.

**Scheme 70:**
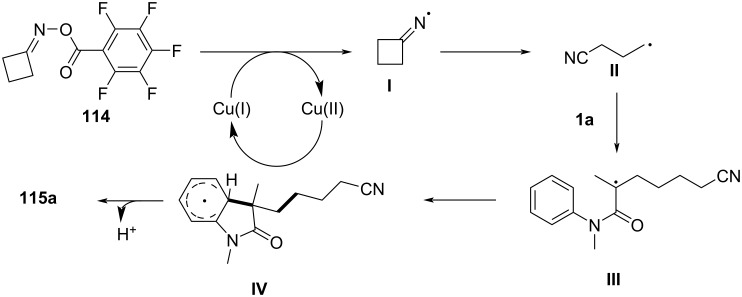
Plausible mechanism for the copper-catalyzed cyanoalkylarylation of activated alkenes.

Mechanistic studies confirmed the radical nature of the transformation (Scheme 70). TEMPO radical trapping experiments revealed the involvement of a cyanoalkyl radical, and the reaction did not proceed without the copper catalyst or light, underscoring the importance of photochemical activation. The proposed mechanism initiates with a single-electron-transfer (SET) event from Cu(I) to the cyclobutanone oxime ester, leading to the formation of an iminyl radical species **I**. This radical subsequently undergoes ring-opening via C–C bond scission, followed by structural rearrangement to produce a γ-cyanoalkyl radical intermediate **II**. The resulting carbon-centered radical then adds across the olefin moiety of the acrylamide, furnishing a tertiary alkyl radical **III**. This intermediate undergoes intramolecular cyclization, generating radical species **IV**. A second SET process, mediated by Cu(II), oxidizes this radical to form a carbocation **V**, which, upon deprotonation, delivers the final product **115a** and simultaneously regenerates the Cu(I) catalyst, thus completing the catalytic cycle.

**Scheme 71:**
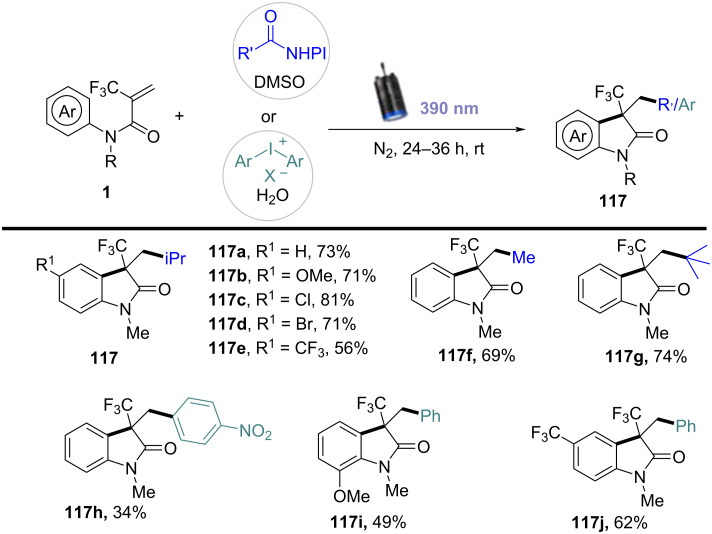
Photoinduced alkyl/aryl radical cascade for the synthesis of quaternary CF_3_-attached oxindoles.

In 2023, a study described a photoinduced alkyl/aryl radical cascade for the synthesis of quaternary CF_3_-containing oxindoles and indoline alkaloids (Scheme 71) [40]. This reaction was carried out under mild photocatalytic conditions, utilizing light to generate reactive alkyl or aryl radicals, which then underwent a cascade reaction to form the desired products. Notably, this method represented a significant advancement in the incorporation of the CF_3_ group into bioactive molecules, achieved without the need for harsh reagents or extreme conditions. The substrate scope was thoroughly explored, demonstrating that a wide variety of alkyl and aryl groups, including both electron-rich and electron-deficient aromatic rings, could be incorporated into the reaction. The CF_3_ group was successfully introduced with high regioselectivity, and the method exhibited excellent tolerance to various functional groups, such as halogens, methoxy, and nitro groups. These results highlight the broad applicability of the approach for synthesizing diverse oxindole and indoline derivatives.

**Scheme 72:**
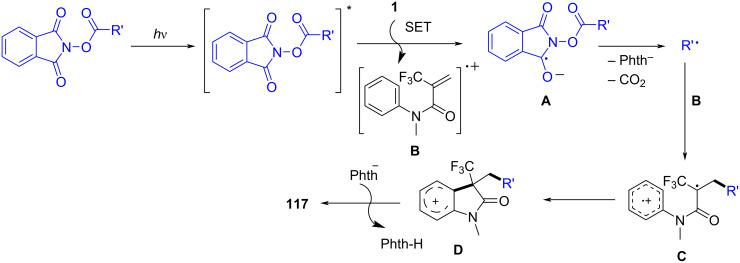
Plausible photoinduced electron-transfer (PET) mechanism.

Furthermore, the reaction mechanism was carefully studied and proposed to involve the photoexcitation of a catalyst or substrate under UV or visible light, leading to the generation of alkyl or aryl radicals (Scheme 72). These radicals then undergo a cascade process, including hydrogen-atom transfer and carbon–carbon-bond formation, ultimately leading to the incorporation of the CF_3_ group into the product. To confirm the radical nature of the reaction, isotopic labeling and radical trapping experiments were conducted, providing strong evidence for the detailed steps of the mechanism.

**Scheme 73:**
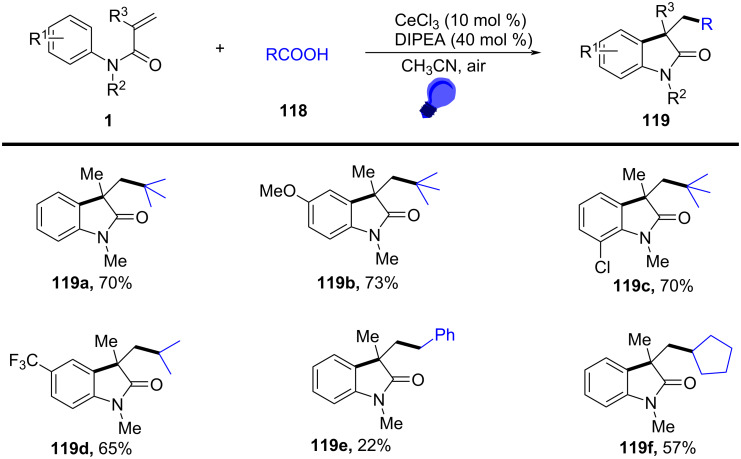
Photoinduced cerium-mediated decarboxylative alkylation cascade cyclization.

In 2023, Roy and co-workers presented a novel photoinduced cerium-mediated decarboxylative alkylation cascade cyclization method for the efficient synthesis of *N*-arylacrylamides and *N*-acryl-2-arylbenzimidazole derivatives (Scheme 73) [41]. This method utilized cerium salts activated by UV light to generate reactive radicals, which initiated the decarboxylation and subsequent cascade cyclization. The reaction efficiently formed complex heterocyclic compounds in a single step, providing a versatile approach for synthesizing a wide range of substituted aromatic systems. A broad substrate scope was demonstrated, with the reaction tolerating various electron-rich and electron-deficient aromatic systems, as well as functional groups such as halides and methoxy, offering flexibility for late-stage functionalization in organic synthesis.

**Scheme 74:**
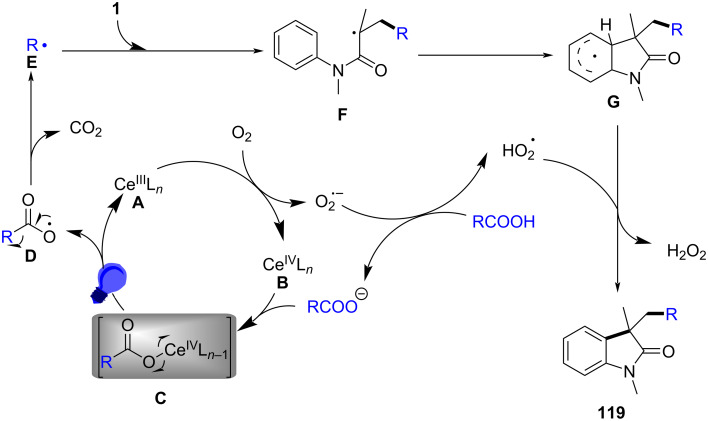
Plausible reaction mechanism for the decarboxylative radical-cascade alkylation/cyclization.

Control experiments confirmed the critical roles of both cerium salts and UV light in activating the reaction, with radical intermediates playing a key role in the mechanism. The proposed mechanism, shown in Scheme 74, involves a photoinduced cerium reduction to generate cerium(III) **A**, which facilitates decarboxylation and produces alkyl radicals **E** that undergoes chain propagation and cascade cyclization to deliver the final products **119**.

### *N*-Arylalkenes: carbonyl, aryl C(sp^2^)-centered radicals

**Scheme 75:**
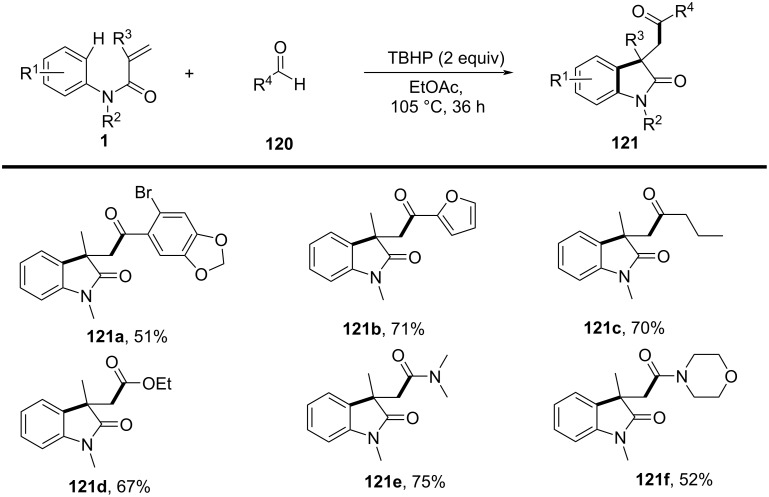
Metal-free oxidative tandem coupling of activated alkenes.

In 2013, a metal-free TBHP-mediated oxidative tandem coupling of activated alkenes with carbonyl C(sp^2^)–H bonds and aryl C(sp^2^)–H bonds for the selective synthesis of 3-(2-oxoethyl)indolin-2-ones was investigated (Scheme 75) [42]. A variety of aldehydes with a carbonyl C(sp^2^)–H bond, including aldehydes, formates, formamides, and *N*-arylacrylamides, were suitable for these conditions, with acceptable isolated yields ranging from 51% to 75%.

**Scheme 76:**
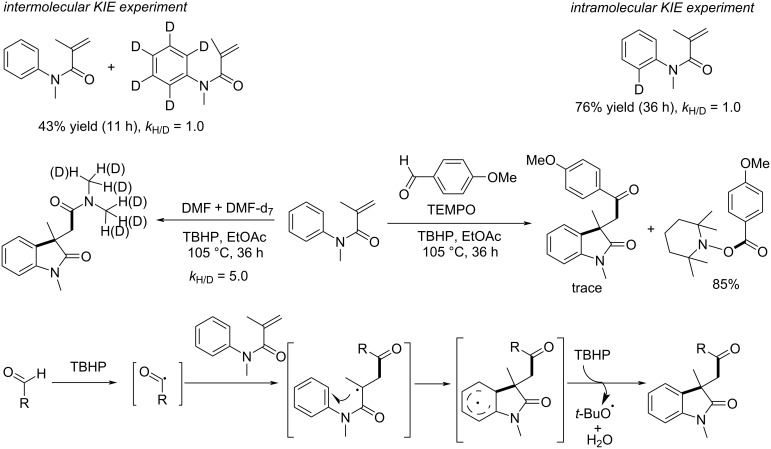
Control experiments and possible mechanism for 1,2-carbonylarylation of alkenes with carbonyl C(sp^2^)–H bonds.

Control experiments, including intramolecular kinetic isotope effect (*k*_H_/*k*_D_ = 1.0) and radical inhibitor studies, revealed that the arylation step might proceed via a free radical mechanism. Additionally, a significant kinetic isotope effect (*k*_H_/*k*_D_ = 5.0) was observed in the reaction of amide **1a** with DMF and DMF-d_7_, suggesting that cleavage of the carbonyl C–H bond was the rate-limiting step. Based on these control experiments, a plausible mechanism was proposed, as outlined in Scheme 76. Initially, in the presence of TBHP, the aldehydic hydrogen is abstracted by alkoxy and/or hydroxy radicals to yield an aldehydic radical, which then adds to the carbon–carbon double bond of the amide, forming an alkyl radical intermediate. This intermediate undergoes intramolecular cyclization to give an aryl ring radical. Finally, abstraction of an aryl hydrogen by TBHP furnishes 3-(2-oxoethyl)indolin-2-one **121**.

**Scheme 77:**
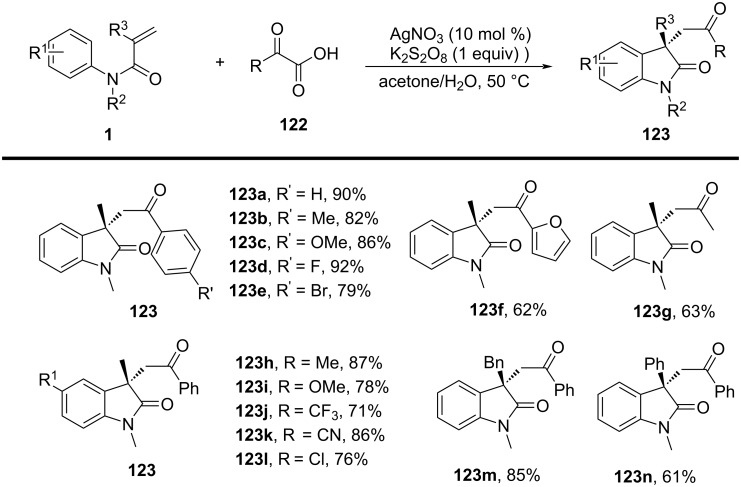
Silver-catalyzed acyl-arylation of activated alkenes with α-oxocarboxylic acids.

In 2013, Guo and Duan developed a silver-catalyzed acylarylation of activated alkenes with easily available α-oxocarboxylic acids (Scheme 77) [43]. This reaction was conducted under mild conditions and proved efficient in providing a variety of functionalized oxindoles. The method was characterized by its broad functional group tolerance, enabling the synthesis of the desired oxindoles in good to excellent yields. Specifically, substrates such as α-oxocarboxylic acids and activated alkenes used in this process could be substituted with various electron-donating and electron-withdrawing groups, demonstrating the method’s broad substrate scope (**123a**–**n**). This feature made the method suitable for the synthesis of complex molecules, including biologically active compounds.

**Scheme 78:**
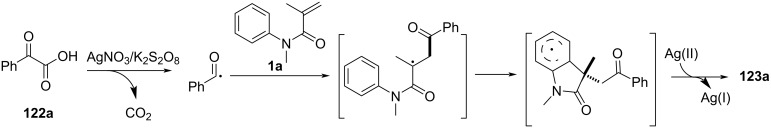
Proposed mechanism for the decarboxylative acylarylation of acrylamides.

Regarding the reaction mechanism, the process was believed to proceed via a radical pathway (Scheme 78). Initially, the silver catalyst facilitates the generation of a decarboxylative radical from the α-oxocarboxylic acid. This radical then adds to the activated alkene, triggering a cyclization step that leads to the formation of the oxindole ring. The mechanism involves key steps such as radical formation, radical addition, and subsequent cyclization, culminating in the formation of the functionalized oxindole product.

**Scheme 79:**
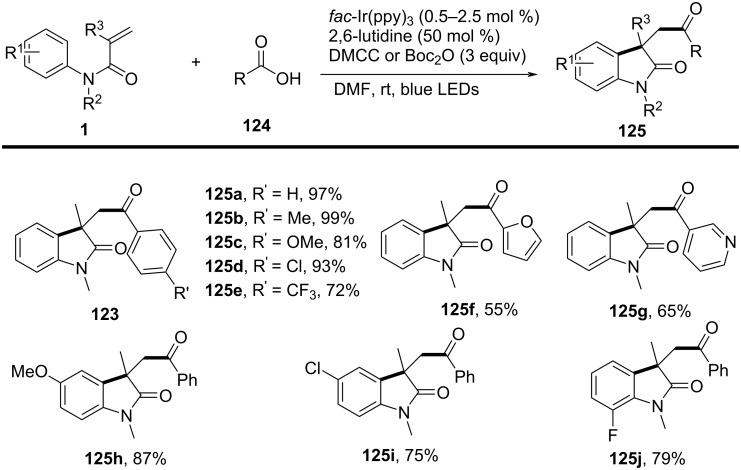
Visible-light-mediated tandem acylarylation of olefines with carboxylic acids.

In 2015, Wallentin and co-workers presented a visible-light photoredox catalytic method for generating acyl radicals from simple and abundant carboxylic acids (Scheme 79) [44]. The reaction proceeds via a redox-neutral process, with the transient formation of a reactive anhydride intermediate playing a central role. This approach enables the efficient synthesis of high-value heterocyclic compounds under mild conditions, eliminating the need for UV irradiation, high temperatures, elevated CO pressures, tin reagents, or peroxides. Regarding substrate applicability, a wide variety of carboxylic acids, including both aromatic and heteroaromatic derivatives, were successfully employed. The method demonstrated tolerance to various functional groups, such as electron-donating and electron-withdrawing substituents, halides, and alkyl groups, thus offering significant flexibility in substrate selection (**125a**–**g**). This broad substrate scope enabled the synthesis of a diverse range of acyl radicals and, subsequently, functionalized heterocyclic compounds, without requiring major adjustments to the reaction conditions.

**Scheme 80:**
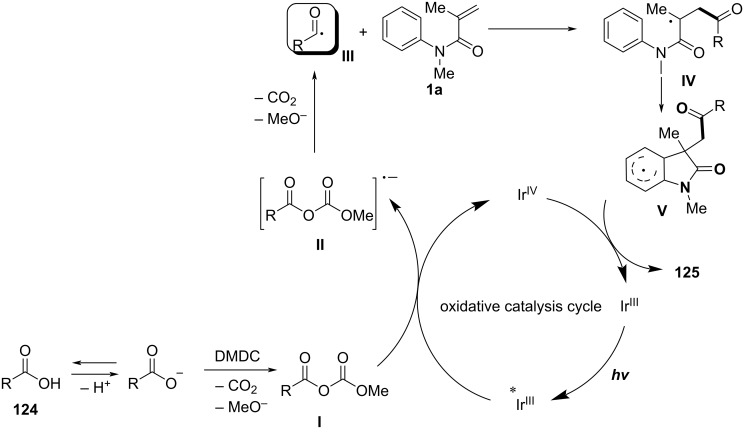
Proposed mechanism for the radical cascade cyclization with acyl radical via visible-light photoredox catalysis.

The reaction mechanism involves photoredox activation of the carboxylic acid, generating a reactive anhydride intermediate **II** (Scheme 80). This intermediate undergoes homolytic cleavage to yield the acyl radical **III**, which then reacts with *N*-arylacrylamides to form the desired heterocyclic product **125**. Stern–Volmer studies confirmed that the mixed anhydride was the key quencher of the excited photocatalyst, supporting the proposed mechanism.

**Scheme 81:**
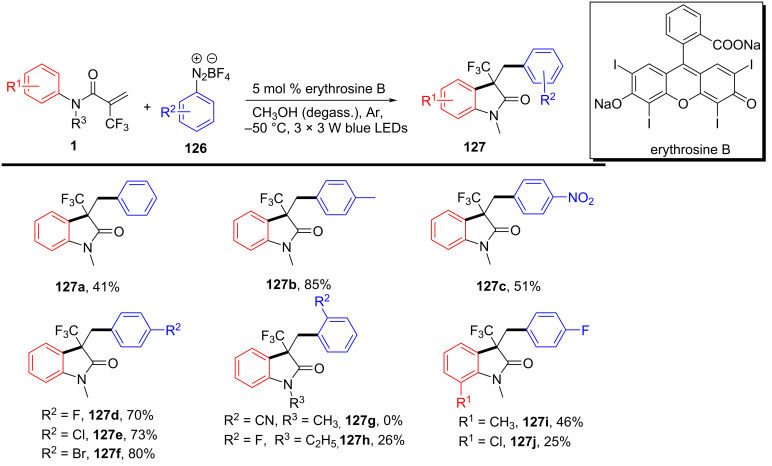
Erythrosine B-catalyzed visible-light photoredox arylation-cyclization of *N*-arylacrylamides with aryldiazonium tetrafluoroborate salts.

In 2016, Tóth and colleagues developed a visible-light-induced photoredox arylation-cyclization process for *N*-alkyl-*N*-aryl-2-(trifluoromethyl)acrylamides, employing erythrosine B as a cost-effective organic photocatalyst under metal-free conditions (Scheme 81) [45]. This reaction utilized readily available aryldiazonium tetrafluoroborate salts as aryl radical precursors, enabling the formation of 3-(trifluoromethyl)indolin-2-one derivatives under blue LED irradiation at low temperatures (−50 °C). This method offers a sustainable and practical approach for constructing fluorinated heterocycles without relying on transition metals or harsh reagents. Regarding substrate compatibility, a wide range of aryldiazonium salts, including electron-rich and halogen-substituted variants (e.g., -Me, -F, -Cl, -Br), were successfully converted to the corresponding products **127b**, **127d**–**f**) in moderate to excellent yields. Nonetheless, the transformation showed sensitivity to electronic and steric effects; *ortho*-substituted and strongly electron-deficient diazonium salts typically led to reduced yields or failed conversions (e.g., **127c**, **127g**, and **127h**). Additionally, various substituted *N*-acrylamides were tested, maintaining consistent reactivity patterns across different amide scaffolds (**127a**, **127i**, and **127j**), underscoring the method’s robustness.

**Scheme 82:**
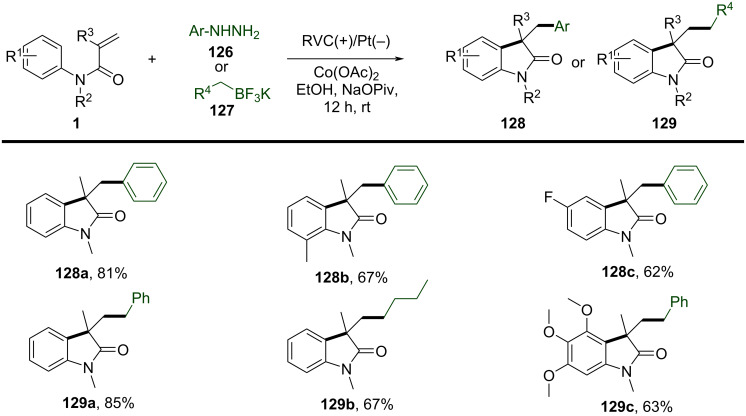
Electrochemical cobalt-catalyzed radical cyclization of *N*-arylacrylamides with arylhydrazines or potassium alkyltrifluoroborates.

In 2018, Yu’s group introduced an efficient electrochemical cobalt-catalyzed method for C–H and N–H oxidation, providing a straightforward and sustainable route for the synthesis of substituted oxindoles (Scheme 82) [46]. The reaction was conducted under mild, environmentally friendly conditions, utilizing the electrochemical process to generate reactive species in situ, thereby avoiding the use of hazardous reagents or extreme reaction conditions. The method demonstrated remarkable versatility in terms of substrate applicability. Various substrates, including both aliphatic and aromatic compounds, were successfully oxidized to yield substituted oxindoles. Additionally, the reaction tolerated a wide range of functional groups, such as halides, alkyl groups, and both electron-donating and electron-withdrawing substituents. This broad functional group tolerance enabled the synthesis of a diverse array of oxindole derivatives, making the method particularly valuable for the synthesis of complex molecules.

**Scheme 83:**
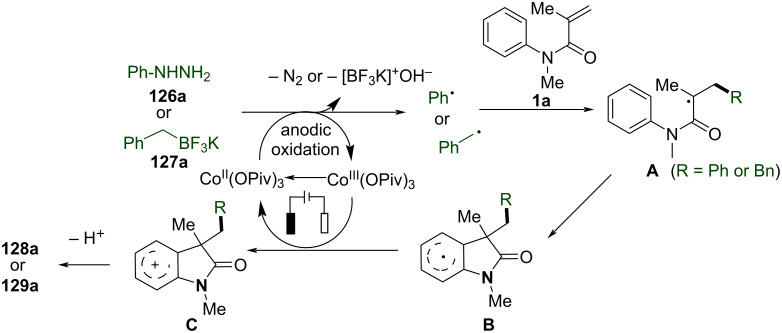
Proposed mechanism of radical cascade cyclization via electrochemical cobalt catalysis.

The proposed reaction mechanism, shown in Scheme 83, involves the electrochemical activation of the cobalt catalyst, which facilitates the oxidation of C–H or N–H bonds. Initially, the cobalt catalyst is electrochemically activated, enabling it to interact with the substrate and generate a metal-oxidized intermediate. This intermediate undergoes further oxidation, ultimately leading to the formation of the desired product.

**Scheme 84:**
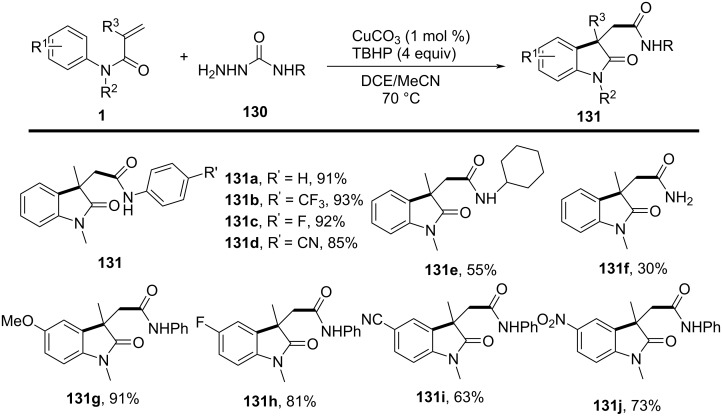
Copper-catalyzed oxidative tandem carbamoylation/cyclization of *N*-arylacrylamides with hydrazinecarboxamides.

A copper-catalyzed oxidative tandem carbamoylation/cyclization of *N*-arylacrylamides with hydrazinecarboxamides to synthesize 2-(oxindol-3-yl)acetamides was reported by Tian’s group in 2018 (Scheme 84) [47]. The system involved the use of 1 mol % CuCO_3_ as the catalyst and 4 equivalents of aqueous TBHP as the oxidant in a 1:1 mixture of 1,2-dichloroethane (DCE) and acetonitrile (MeCN) at 70 °C under nitrogen. Regarding the substrate scope, *N*-arylacrylamides bearing diverse electron-withdrawing (-F, -CN, -NO_2_) and electron-donating (-OMe) substituents on the aryl ring were well-tolerated, delivering products in moderate to excellent yields (63–91%). Additionally, hydrazinecarboxamides with secondary alkyl, aryl, or pyridinyl groups performed effectively, yielding secondary acetamides, though primary acetamides were obtained in lower yields.

**Scheme 85:**
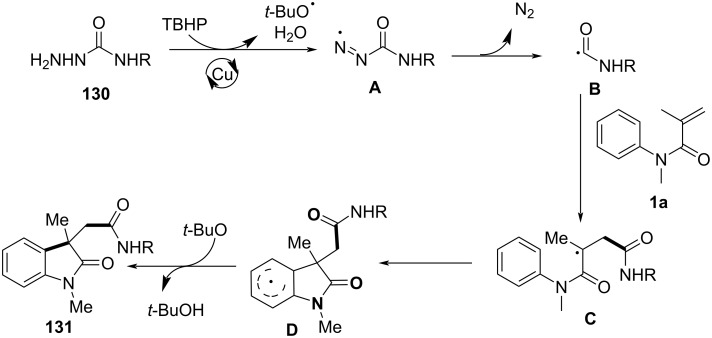
Proposed reaction mechanism for the radical cascade cyclization by copper catalysis.

The proposed mechanism, shown in Scheme 85, involves a radical pathway initiated by Cu(II)-mediated oxidation of hydrazinecarboxamide with TBHP, generating a diazene radical intermediate **A**. Subsequent nitrogen extrusion forms a carbamoyl radical **B**, which undergoes regioselective addition to the *N*-arylacrylamide alkene. The resulting alkyl radical undergoes 5-*exo*-*trig* cyclization, followed by hydrogen abstraction by the *tert*-butoxy radical to yield the oxindole product **131**.

**Scheme 86:**
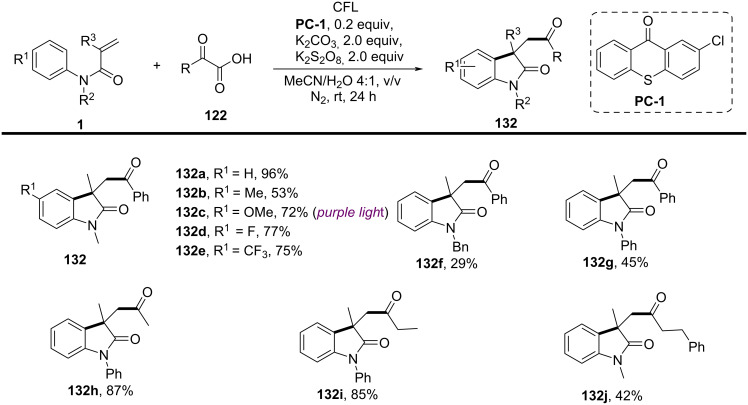
Visible-light-driven radical cascade cyclization reaction of *N*-arylacrylamides with α-keto acids.

In 2022, Sun, Yu, and Wu’s group reported a metal-free, visible-light-driven cascade cyclization reaction for synthesizing 3-methyl-3-acetophenone-2-oxindoles via a benzoyl radical (Scheme 86) [48]. This reaction utilized α-keto acids as radical precursors, 2-chlorothioxanthone (**PC-1**) as the organic photocatalyst, and K_2_S_2_O_8_ as an oxidant under irradiation with white or purple light (23 W LED). Regarding substrate scope, *N*-arylacrylamides bearing electron-donating (e.g., -Me, -OMe) and electron-withdrawing groups (e.g., -F, -CF₃) on the aryl ring were compatible, delivering 2-oxindoles in moderate to excellent yields. However, substrates with strong electron-donating groups (-OMe) required higher-energy purple light for conversion. Notably, *N*-alkyl (-Me, -Bn) or *N*-aryl substituents were tolerated, while *N*–H or electron-withdrawing *N*-substituents (-Ts, -Ac) inhibited reactivity.

**Scheme 87:**
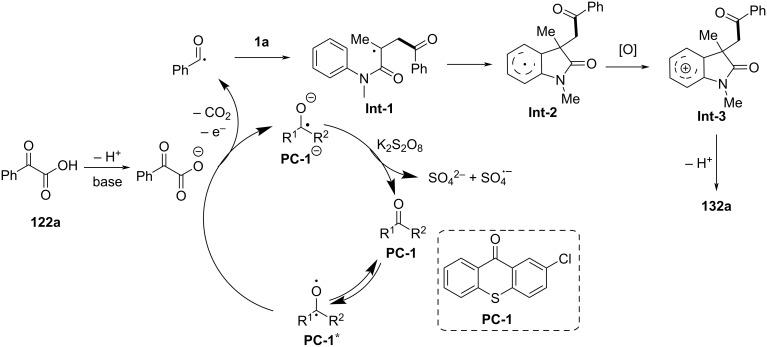
Proposed mechanism of visible-light-driven cascade cyclization reaction.

The proposed mechanism involves a radical pathway initiated by photoexcitation of the photocatalyst, which oxidizes α-keto acids to generate the benzoyl radical (Scheme 87). This radical then undergoes regioselective addition to the acrylamide alkene, forming intermediate (**Int-1**), followed by 5-*exo-trig* cyclization to yield intermediate (**Int-2**). Subsequent oxidation and deprotonation furnish the final 2-oxindoles **132a**. Mechanistic evidence includes complete inhibition by TEMPO (with TEMPO-carbamoyl adduct detection via ESI–MS), radical clock experiments, and kinetic isotope effects (KIE ≈ 1.0), confirming a non-rate-determining C–H cleavage step and electrophilic radical addition.

**Scheme 88:**
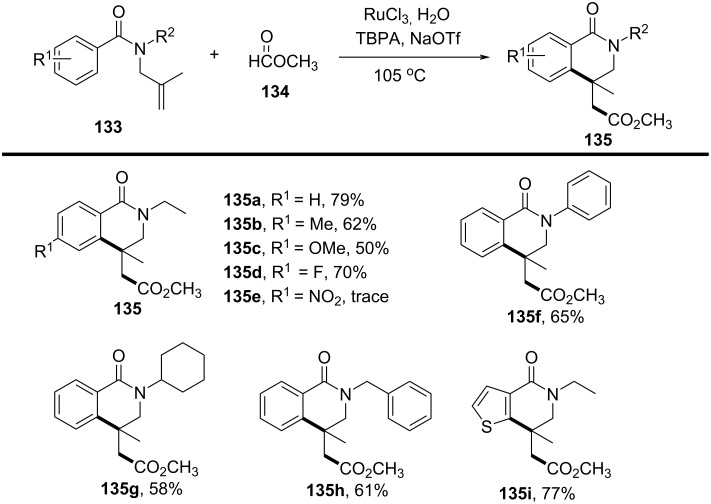
Peroxide-induced radical carbonylation of *N*-(2-methylallyl)benzamides with methyl formate.

In 2022, another research presented a novel method for the radical esterification of unactivated alkenes, using methyl formate as a C_1_ building block, thus providing a sustainable alternative to conventional carbonylation approaches (Scheme 88) [49]. In this system, the reaction is initiated by a peroxide-induced radical carbonylation, where methyl formate acts as the precursor for the methoxycarbonyl radical. This process is catalyzed by RuCl_3_ under mild conditions, allowing for a one-pot synthesis of biologically significant 4-[(methoxycarbonyl)methyl]-3,4-dihydroisoquinolinones. The substrate scope of this method was comprehensively explored, revealing that a variety of *N*-(2-methylallyl)benzamides can be successfully used under the optimized reaction conditions. Substrates bearing both electron-donating and electron-withdrawing groups on the aromatic ring, such as methyl, methoxy, chloro, and fluoro, led to the desired products with yields ranging from 50% to 79%. Furthermore, different *N*-protecting groups, including phenyl, benzyl, and cyclohexyl, showed compatibility with the reaction, resulting in a diverse range of functionalized dihydroisoquinolinones. However, substrates with strong electron-withdrawing groups, such as -NO_2_, showed reduced reactivity, indicating that such groups may destabilize the reaction intermediates.

**Scheme 89:**
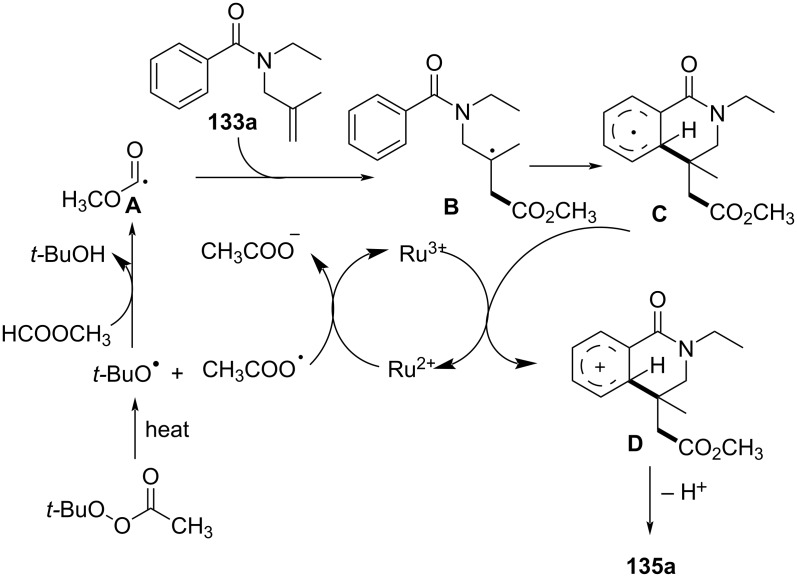
Proposed cyclization mechanism of peroxide-induced radical carbonylation with *N*-(2-methylallyl)benzamides.

The proposed reaction mechanism was thoroughly investigated through radical trapping experiments using BHT and TEMPO, confirming that the transformation proceeded via a radical pathway (Scheme 89). Initially, the peroxide decomposes to generate *tert*-butoxyl radicals, which abstract a hydrogen atom from methyl formate, producing methoxycarbonyl radicals **A**. These radicals then add to the *N*-(2-methylallyl)benzamide, forming a radical intermediate **B**. Subsequently, the aryl group captures this intramolecular radical, resulting in a dearomatized aryl radical intermediate **C**, which undergoes oxidation through a single-electron transfer (SET) to form a cation **D**. Finally, this cation is deprotonated, yielding the final product **135a**. The catalytic cycle is completed with the oxidation of Ru(II) to Ru(III).

**Scheme 90:**
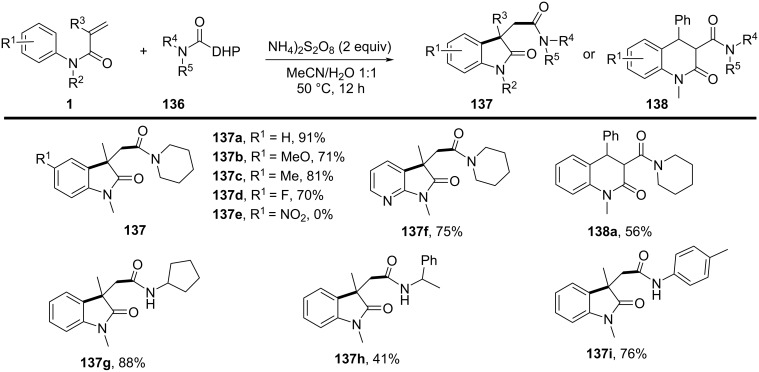
Persulfate promoted carbamoylation of *N*-arylacrylamides and *N*-arylcinnamamides.

In this context, in 2023, Sun, Zhou and colleagues introduced a novel method for the oxidative carbamoylation of *N*-arylacrylamides using 4-carbamoyl-Hantzsch esters as carbamoyl radical precursors, promoted by persulfates (Scheme 90) [50]. This reaction offers a straightforward approach for synthesizing a variety of 3,3-disubstituted oxindoles and 3,4-disubstituted dihydroquinolin-2(1*H*)-ones in moderate to good yields. The scope of the reaction was extensively explored, demonstrating the broad compatibility of various 4-carbamoyl-Hantzsch esters. The substrates included both cyclic and acyclic tertiary amides, secondary amides, and heteroaryl amides, all of which underwent successful carbamoylation to yield the corresponding oxindoles in satisfactory to excellent yields. Additionally, *N*-arylacrylamides with a range of functional groups, including methoxy, methyl, and halides, were also efficiently converted into the desired products. Notably, substrates with electron-withdrawing groups such as F and electron-donating groups such as methyl on the aromatic ring were well tolerated. However, the reaction was less effective with substrates containing a nitro group or a free NH group on the amide.

**Scheme 91:**
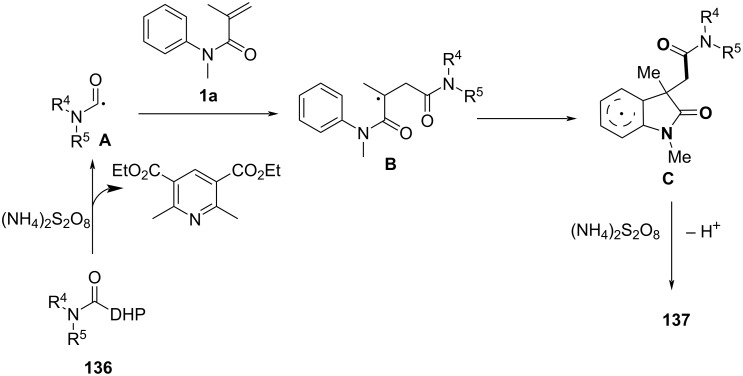
Proposed mechanism for the persulfate promoted radical cascade cyclization reaction of *N-*arylacrylamides and *N*-arylcinnamamides.

A detailed mechanistic study suggested that the transformation proceeded via a radical pathway, as depicted in Scheme 91. Persulfates were found to oxidize the 4-carbamoyl-Hantzsch ester, generating the carbamoyl radical through a single-electron transfer (SET) process. The radical then adds to the *N*-arylacrylamide, forming an alkyl radical, which undergoes intramolecular cyclization. This cyclization results in the formation of an intermediate that is subsequently aromatized to yield the final product. Radical trapping experiments with TEMPO confirmed the involvement of free radicals, further supporting the mechanistic pathway.

**Scheme 92:**
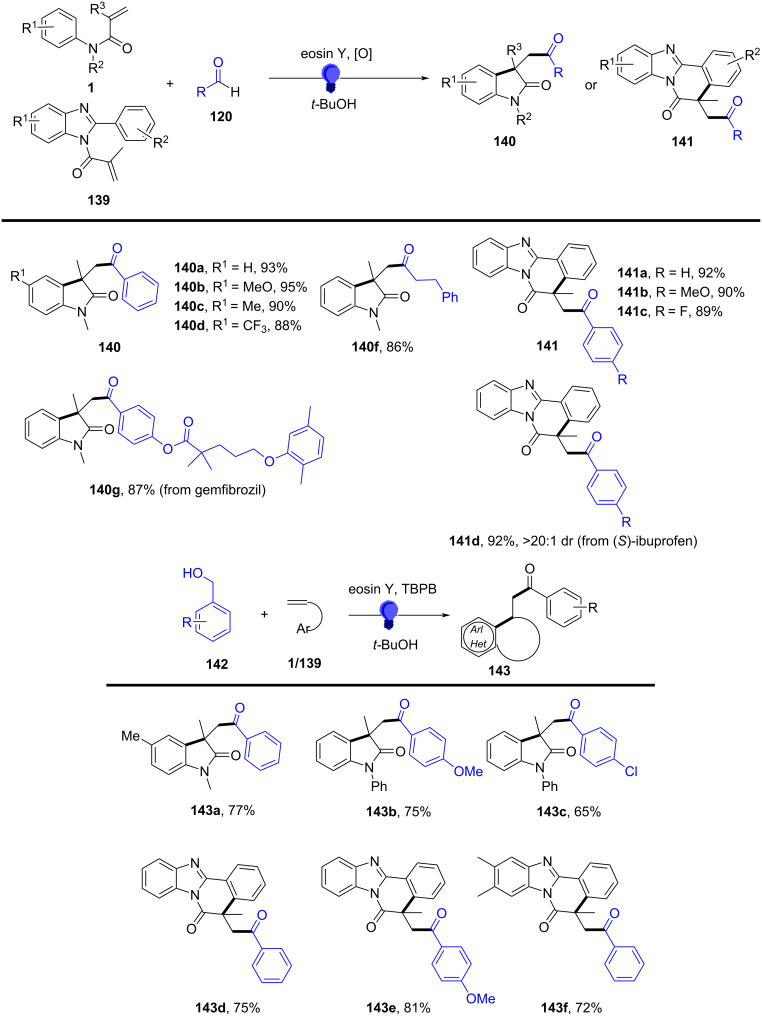
Photocatalyzed carboacylation with *N*-arylpropiolamides*/N-*alkyl acrylamides.

In 2023, Roy and co-workrs reported an organo-photocatalyzed carbacylation reaction using aldehydes and alcohols as acyl precursors under visible-light irradiation (440 nm, 40 W blue LED) with eosin Y (4 mol %) as the catalyst and TBHP or TBPB as the oxidant in *t*-BuOH at ambient temperature (Scheme 92) [51]. Optimized conditions achieved high yields (up to 95%) for various heterocycles.

The substrate scope showed broad compatibility: (1) *N*-methacryloyl-2-phenylbenzimidazoles with both electron-donating and electron-withdrawing substituents on aromatic aldehydes gave benzimidazo[2,1-*a*]isoquinolin-6(5*H*)-ones in 86–95% yields. (2) *N*-Alkyl/*N*-aryl acrylamides reacted with aldehydes to yield 3,3-disubstituted oxindoles in 89–92% yields, accommodating various substituted benzaldehydes and even unprotected hydroxybenzaldehyde. (3) Benzyl alcohols successfully delivered oxindoles and benzimidazo-isoquinolinones (65–81%), while aliphatic alcohols did not react.

**Scheme 93:**
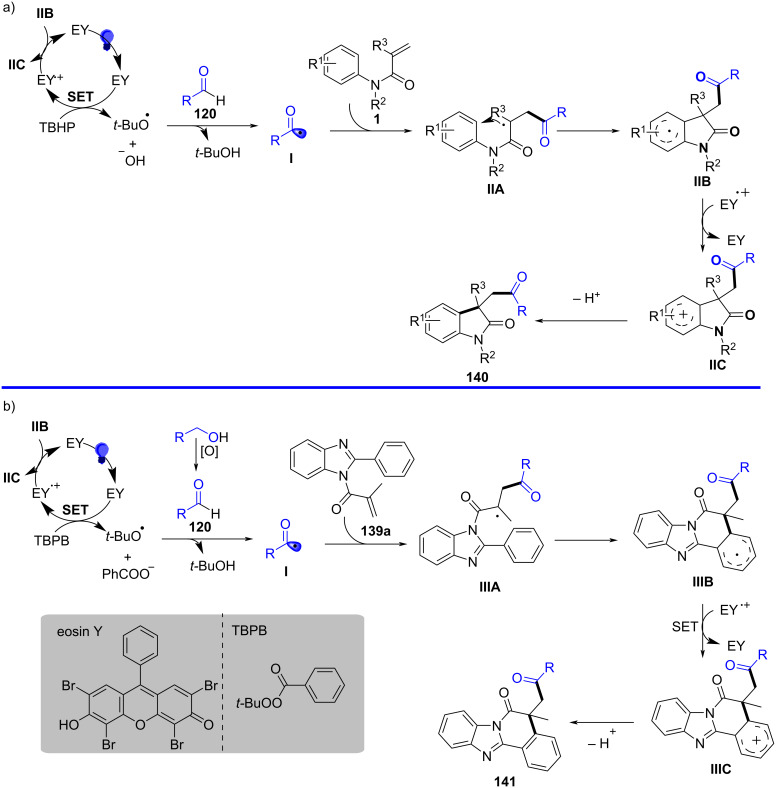
Plausible mechanism for the photoinduced carboacylation of *N*-arylpropiolamides/*N-*alkyl acrylamides.

Mechanistic studies, including radical inhibition (TEMPO/BHT quenching), intermediate trapping (GC–MS detection of acyl-TEMPO adducts), and spectroscopic analyses (UV–vis, fluorescence quenching), supported a radical cascade pathway. Upon irradiation (Scheme 93), eosin Y is excited (EY*) and undergoes single-electron transfer (SET) with TBHP, generating *tert*-butoxy radicals (*t*-BuO**^.^**), which abstracts a hydrogen atom from aldehydes to form acyl radicals. These radicals add to alkenes, triggering cyclization to afford intermediates (**IIA**/**IIIA**), which are oxidized by EY^+·^ to regenerate EY and yield final products via aromatization or further oxidation. For alcohols, a sequential oxidation to aldehydes preceded acyl radical generation. Late-stage functionalization of pharmaceuticals (ibuprofen **141d**, gemfibrozil **140g**) further highlighted the synthetic utility, achieving drug–heterocycle hybrids with retained stereochemistry.

**Scheme 94:**
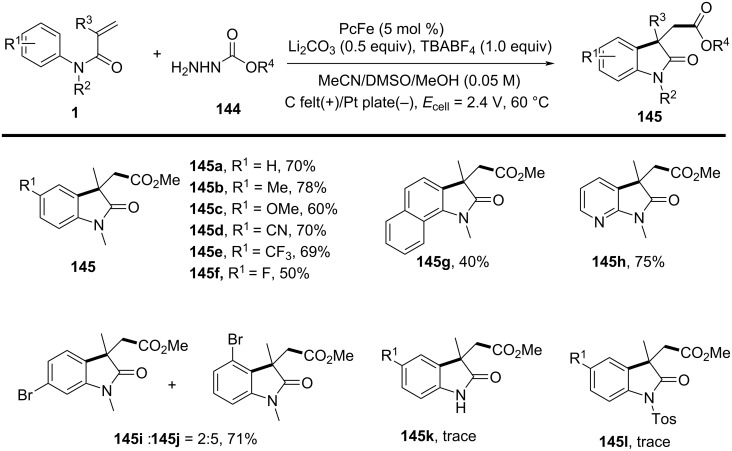
Electrochemical Fe-catalyzed radical cyclization with *N*-arylacrylamides.

In 2023, Song and colleagues disclosed an electrochemical Fe-catalyzed radical cyclization protocol for the synthesis of 3,3-disubstituted 2-oxindoles bearing ester groups from readily accessible *N*-arylacrylamides and carbazates (Scheme 94) [52]. The reaction was conducted in an undivided cell under constant current electrolysis (6.0 mA) using iron(II) phthalocyanine (PcFe) as the catalyst, TBABF as the electrolyte, and a mixed solvent of MeCN/DMSO 2:1 with methanol as the proton source. A carbon felt anode and Pt cathode were employed, enabling efficient radical generation and proton reduction. Substrate scope investigations demonstrated broad compatibility; *N*-arylacrylamides with electron-donating (-OMe, -Me) or electron-withdrawing (-CN, -CF_3_, halides) substituents on the aryl ring were tolerated, affording oxindoles in 40–78% yields. *Meta*-substituted substrates exhibited regioselective cyclization, consistent with radical-mediated pathways. However, *N–*H or *N*–Tos acrylamides failed to react in this environment.

**Scheme 95:**
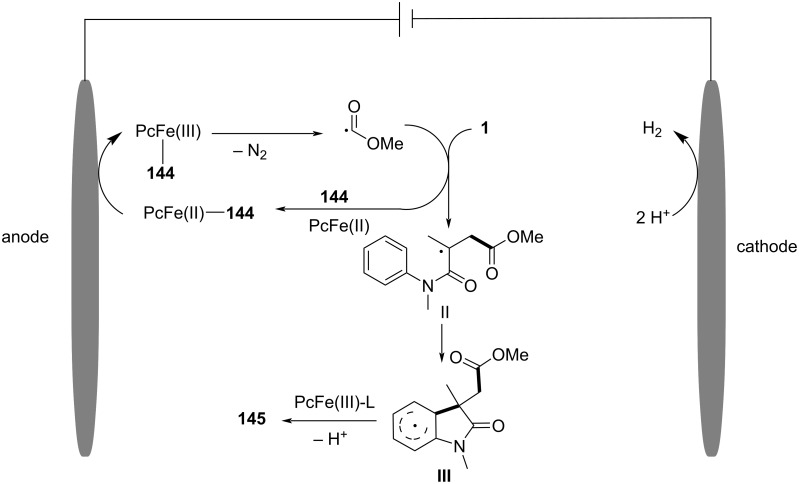
Plausible mechanism for the electrochemical Fe-catalysed radical cyclization of *N*-phenylacrylamide.

Mechanistic studies, including radical trapping with TEMPO and cyclic voltammetry, confirmed a methoxycarbonyl radical **I** generated via Fe-mediated carbazate oxidation as the key intermediate. Coordination of carbazate to Fe(II) lowers the oxidation potential, facilitating radical generation at −150 mV (vs 300 mV for uncatalyzed oxidation) (Scheme 95). The methoxycarbonyl radical undergoes addition to the acrylamide’s electron-deficient alkene, forming a carbon-centered radical **II**, which cyclizes intramolecularly to yield intermediate **III**. Subsequent Fe-catalyzed single-electron oxidation and rearomatization furnishes the oxindole product **145**

**Scheme 96:**
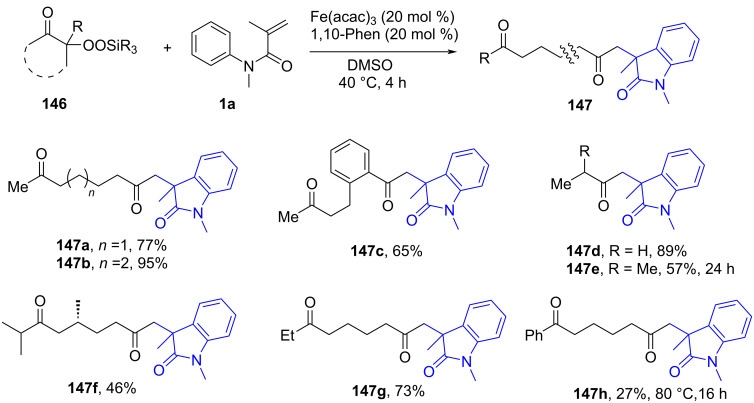
Substrate scope of the selective functionalization of various α-ketoalkylsilyl peroxides with methacrylamide **1a**.

In 2024, Kato, Liu, Maruoka and colleagues presented a novel strategy for Fe-catalyzed radical transformations using functionalized alkylsilyl peroxides, which served as precursors for reactive acyl radicals (Scheme 96) [53]. These radicals were generated through a reductive β-scission mechanism and subsequently reacted with methacrylamides to form valuable carbon–carbon and carbon–heteroatom bonds under mild conditions. Moreover, substrate compatibility was broad, a variety of α-ketoalkylsilyl peroxides, including both cyclic and acyclic, substituted and unsubstituted, aromatic and aliphatic, successfully reacted with methacrylamide under the optimized reaction conditions.

**Scheme 97:**
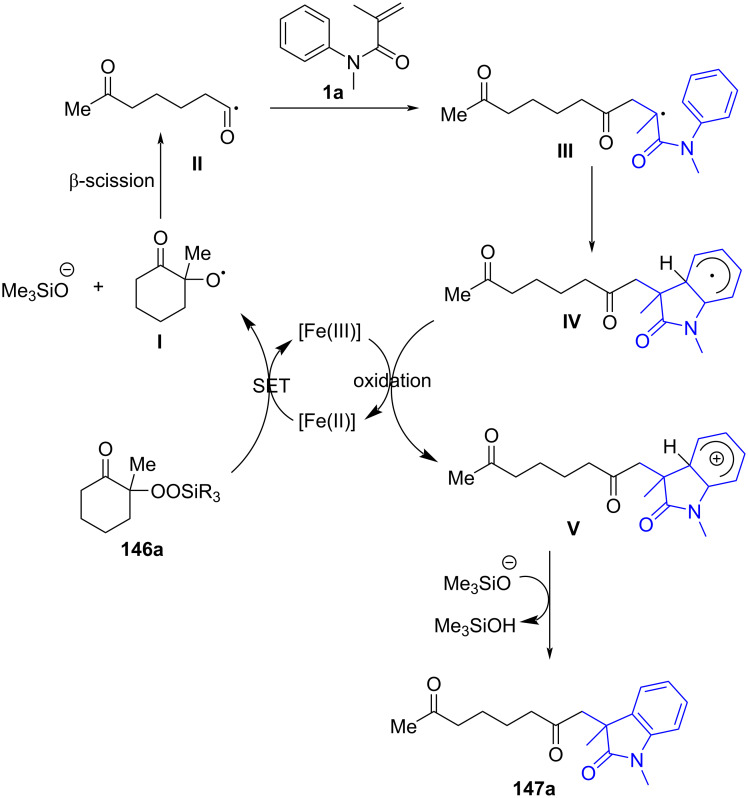
Proposed reaction mechanism for the Fe-catalyzed reaction of alkylsilyl peroxides with methacrylamide **1a**.

Mechanistic investigations indicated that the reaction proceeded through the formation of acyl radicals **I**, generated by single-electron transfer (SET) from Fe(II) species (Scheme 97). These acyl radicals underwent β-scission, leading to the formation of reactive intermediates **II** that either undergo conjugate addition–cyclization or addition–rearomatization, depending on the substrate. Radical trapping experiments with TEMPO further confirmed the involvement of acyl radicals in the process, verifying the proposed mechanism.

**Scheme 98:**
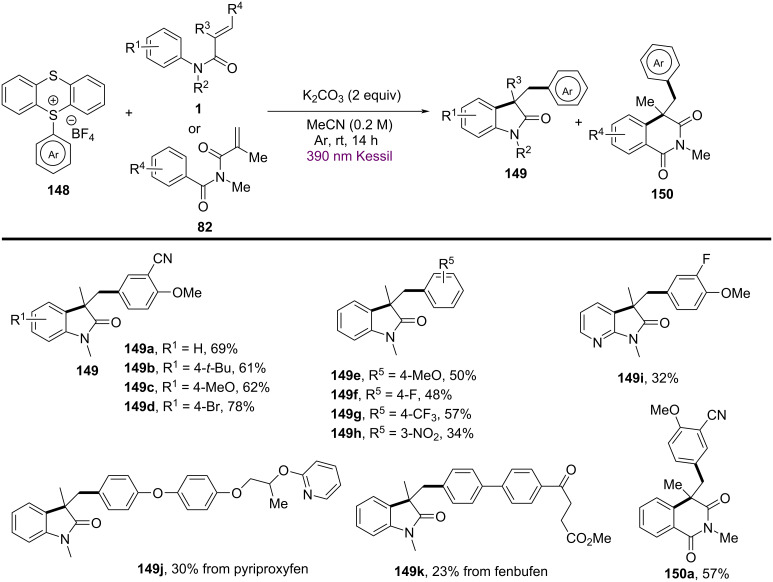
EDA-complex mediated C(sp^2^)–C(sp^3^) cross-coupling of TTs and *N*-methyl-*N*-phenylmethacrylamides.

In a recent study, Vallribera, Gimbert-Suriñach and Granados reported a photochemical synthesis of benzyloxindoles via electron donor–acceptor (EDA) complex photoactivation using thianthrenium salts (TTs) and potassium carbonate under exogenous photocatalyst-free conditions (Scheme 98) [54]. The reaction was conducted in acetonitrile under 390 nm purple light irradiation, with TTs as electron acceptors, K_2_CO_3_ as the electron donor, and *N*-arylacrylamides as radical traps. Optimization revealed that a mixed solvent system (MeCN) with K_2_CO_3_ (2.0 equiv) and excess acrylamide (2.5 equiv) delivered optimal yields (up to 72%), while control experiments confirmed the necessity of light and the EDA complex formation. Subsequently, substrate scope investigations demonstrated broad compatibility: TTs bearing electron-withdrawing (-NO_2_, -CN, -CF_3_) or electron-donating (-OMe, -Me) groups on the aryl ring, as well as *meta*- and *ortho*-substituted arenes, were tolerated, yielding oxindoles in 34–86% isolated yields. Notably, functional groups such as halides (-F, -Cl, -Br), aldehydes, esters, and pyridine moieties remained intact, and late-stage functionalization of biomolecules (pyriproxyfen, fenbufen) was achieved (23–67% yields). Acrylamides with diverse substituents (-OMe, -Me, -Br) on the aromatic ring and α-carbonyl groups also participated efficiently, affording substituted oxindoles and imides. However, *N*–H or *N*–Tos acrylamides failed to react, and regioselectivity was observed for *meta*-substituted substrates.

**Scheme 99:**
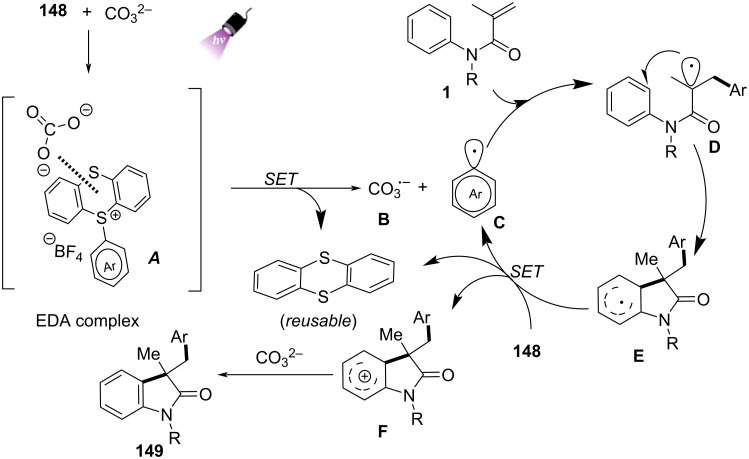
Proposed mechanism for the synthesis of oxindoles via EDA complex.

Mechanistic studies, including UV–vis spectroscopy, TEMPO trapping (reaction inhibition and adduct isolation), and quantum yield measurement (Φ = 6.1), supported a radical chain mechanism initiated by EDA complex formation between TTs and K_2_CO_3_. Photoexcitation induce a single-electron transfer (SET), generating an aryl radical **C** via C–S bond cleavage, which subsequently undergoes Giese addition to the acrylamide **D**. Intramolecular cyclization and rearomatization furnishes the oxindole **149**, while chain propagation was mediated by SET between intermediate radicals and unreacted TTs (Scheme 99).

## Conclusion

The difunctionalization of alkenes to incorporate two functional groups across a double bond has emerged as a powerful transformation that significantly enhances molecular complexity in organic synthesis, offering improved efficiency. Recent advancements in the selective two-component difunctionalization of *N*-arylacrylamides have provided a new and straightforward strategy for constructing complex organic compounds by simultaneously forming two consecutive chemical bonds in a single operation. This review primarily focused on intramolecular 1,2-difunctionalization of alkenes using *N*-arylalkene substrates, including alkyl C(sp^3^)-centered radical functionalization and carbonyl or aryl-C(sp^2^)-centered functionalization. All these processes proceed via oxidative radical-mediated mechanisms, utilizing a variety of carbon radical reagents such as nitriles, amines, alcohols, peroxides, acids, and mineral salts.

In summary, the oxidative radical difunctionalization of *N*-arylalkenes holds immense potential for constructing complex molecules from simple starting materials, initiated by light, thermodynamics, or electricity. With the growing demand for simple intramolecular difunctional compounds in materials science, medicinal chemistry, and biochemistry, it will remain a sustainable and highly relevant area of research in the future.

## Data Availability

Data sharing is not applicable as no new data was generated or analyzed in this study.
